# Artificial intelligence for sleep-disordered breathing: a taxonomy of methods, data modalities, and clinical applications

**DOI:** 10.3389/frsle.2026.1927453

**Published:** 2026-09-18

**Authors:** Tai Dinh, Daniil Lisik, Philippe Fournier-Viger, Dat Tran, Philip S. Yu, Huynh Van Hong, Ding Zou

**Affiliations:** 1 Kyoto College of Graduate Studies for Informatics (KCGI), Kyoto, Japan; 2 Department of Public Health and Clinical Medicine, The OLIN and Sunderby Research Unit, Umeå University, Umeå, Sweden; 3 Krefting Research Center, Institute of Medicine, Sahlgrenska Academy, University of Gothenburg, Gothenburg, Sweden; 4 College of Computer Science and Software Engineering, Shenzhen University, Nanshan, Shenzhen, Guangdong, China; 5 University of Canberra, Bruce, ACT, Australia; 6 Department of Computer Science, University of Illinois at Chicago, Chicago, IL, United States; 7 HCM University of Natural Resources and Environment, Tan Binh, Ho Chi Minh City, Vietnam; 8 Center for Sleep and Vigilance Disorders, Department of Internal Medicine and Clinical Nutrition, University of Gothenburg, Gothenburg, Sweden

**Keywords:** data mining, deep learning, large language models, machine learning, obstructive sleep apnea, precision sleep medicine, sleep-disordered breathing, wearable sensors

## Abstract

Sleep-disordered breathing (SDB), particularly obstructive sleep apnea (OSA), is a highly prevalent group of disorders associated with disrupted sleep, intermittent hypoxemia, excessive daytime sleepiness, neurocognitive impairment, and cardiometabolic morbidity. Although laboratory polysomnography remains central to diagnosis, conventional sleep testing can be costly, labor-intensive, and difficult to scale. Recent advances in data mining, machine learning, deep learning, wearable sensing, mobile health, multimodal analytics, and large language models are creating new opportunities for more accessible, personalized, and efficient SDB care. This review examines recent advances in artificial intelligence for SDB diagnosis and management, with emphasis on polysomnography and home sleep apnea testing, wearable and contactless screening, audio and physiological-signal analysis, imaging and anatomical risk prediction, phenotyping and endotyping, treatment monitoring, continuous positive airway pressure adherence, and clinically deployable explainable AI. We also discuss the emerging role of large language models in patient education, sleep-report summarization, clinical documentation, and decision-support workflows. Finally, we identify major challenges related to data heterogeneity, external validation, interpretability, privacy, fairness, reproducibility, regulation, and real-world integration into sleep medicine. This review aims to provide an evidence-based framework for researchers and clinicians to understand current AI applications and identify future directions for precision diagnosis and management of SDB.

## 1 Introduction

Sleep-disordered breathing (SDB) encompasses a broad spectrum of conditions ranging from habitual snoring to severe obstructive sleep apnea (OSA). These conditions are associated with fragmented sleep, intermittent hypoxemia, daytime symptoms, impaired quality of life, neurocognitive consequences, and increased cardiometabolic risk. Recent AI-oriented studies also emphasize that OSA/SDB is not only a respiratory-event-counting problem, but a heterogeneous condition involving hypoxemia burden, symptom variation, differential cardiovascular risk, and distinct patient phenotypes (Kuhn et al., 2026; Zhang et al., 2026; Blanchard et al., 2022). Despite their high clinical relevance, many individuals with SDB remain undiagnosed or are diagnosed only after substantial delay, which may allow clinically relevant complications to develop and motivates scalable screening tools using clinical variables, wearable sensing, and simplified home-based monitoring (Macey, 2012; Talukder et al., 2024; Zhou et al., 2023). Conventional diagnostic pathways, particularly laboratory polysomnography (PSG), provide rich physiological information but are resource-intensive, labor-demanding, and less scalable for population-level screening. Home sleep apnea testing (HSAT) and simplified monitoring approaches have improved accessibility, but they also create new needs for robust, automated, and interpretable technologies that can support accurate detection, severity estimation, risk stratification, and clinical decision-making (Retamales et al., 2024; Tirachaimongkol et al., 2025). Recent automated respiratory-event scoring and HSAT validation studies illustrate how AI can reduce manual scoring burden while preserving clinically meaningful AHI-related assessment (Nikkonen et al., 2021; Rohrscheib et al., 2026).

Data mining (DM), machine learning (ML), deep learning (DL), and more recently large language models (LLMs) offer a promising technological foundation for this transition. Unlike traditional rule-based approaches, these methods can learn complex patterns from multimodal data, including PSG signals, respiratory airflow, thoracoabdominal effort, oxygen saturation, electrocardiography (ECG), photoplethysmography (PPG), snoring audio, actigraphy, wearable sensor streams, craniofacial images, electronic health records, and patient-reported outcomes (Kara et al., 2025).

Current OSA/SDB models demonstrate this breadth across different sensing modalities and clinical scenarios. Airflow–oximetry fusion and single-lead ECG detection approaches have been investigated for automated OSA/SDB assessment (Peng et al., 2024; Jiao et al., 2024). Non-contact sleep-sound analysis and wearable multi-sensor approaches have also been explored for accessible screening and monitoring applications (Wang et al., 2022; Zhou et al., 2023). Imaging-based models have further been developed for anatomical risk prediction using craniofacial and related features (Chen et al., 2025; Wu et al., 2021).

Such data-driven approaches can support automated diagnosis, screening, and longitudinal monitoring, as demonstrated by recent wearable, contactless, and multimodal systems for OSA/SDB assessment (Kim D. et al., 2025; Lee K.-T. et al., 2025; Olsen et al., 2024). They can also support phenotype discovery and risk stratification (Pataka et al., 2024; Blanchard et al., 2022), as well as treatment-response prediction, adherence support, and remote management (Hanif et al., 2023; Midelet et al., 2023; Wu et al., 2026).

The relevance of AI-based technologies in sleep medicine extends beyond diagnostic automation. SDB is heterogeneous: patients with similar apnea–hypopnea index values may differ substantially in symptoms, hypoxic burden, arousal patterns, anatomical risk factors, comorbidities, treatment tolerance, and long-term outcomes. Therefore, the field is gradually moving from a one-size-fits-all model toward precision sleep medicine, where diagnostic and therapeutic decisions are informed by patient-specific risk profiles, multimodal biomarkers, and dynamic monitoring. ML-based phenotyping, endotyping, and risk-stratification models are especially important in this context because they may help identify clinically meaningful subgroups and support personalized management strategies, as shown by clinical severe-OSA prediction, interpretable risk modeling, and cardiovascular-risk estimation studies (Talukder et al., 2024; Shi et al., 2023; Blanchard et al., 2022). Related work on sleep-perception phenotypes, event-based oxygen-saturation metrics, and speech-based severity estimation further suggests that AI can characterize SDB beyond conventional AHI thresholds (Kuhn et al., 2026; Zhang et al., 2026; TaghiBeyglou et al., 2026).

At the same time, the adoption of AI in SDB care faces several barriers. Sleep datasets differ widely in acquisition protocols, scoring rules, sensors, patient populations, device types, and clinical labels. Many models are trained on single-center datasets and lack external validation, a concern highlighted by work showing that heart rate variability (HRV)-based apnea detectors may not generalize as well as internal cross-validation suggests (Padovano et al., 2022). Black-box predictions may be difficult to interpret in clinical workflows, motivating explainable or confidence-aware approaches that expose relevant signal regions, feature contributions, uncertainty, or decision reliability (Chu et al., 2024; Bark et al., 2023; Barroso-Garcìa et al., 2025). Privacy and deployment concerns are also amplified when data are collected through home-based devices, mobile applications, and cloud platforms, which makes lightweight, on-device, energy-efficient, or wearable-compatible AI particularly relevant (Chen et al., 2024; Ye et al., 2021; Zhao T et al., 2024). In addition, the rapid emergence of LLMs creates opportunities for patient education and clinical documentation, but also raises concerns regarding hallucination, guideline adherence, safety, accountability, and regulatory oversight.

These considerations motivate this review. Rather than treating AI for SDB as a narrow event-detection problem, we organize the literature around the full clinical pathway: screening, diagnosis, risk prediction, phenotyping, treatment selection, adherence monitoring, remote management, and clinical deployment. This broader organization is supported by the current corpus, which includes PSG/HSAT event-scoring models (Nikkonen et al., 2021; Liu Y. et al., 2026), wearable and contactless screening systems (Kwon et al., 2022; Olsen et al., 2024), anatomical and craniofacial risk-prediction models (Hanif et al., 2021; Yoo et al., 2026), and risk-stratification or treatment-planning studies (Blanchard et al., 2022; Hu et al., 2026). The major contributions of this paper are as follows:

– We provide a focused review of recent artificial intelligence, data mining, machine learning, deep learning, and large language model technologies for the diagnosis and management of sleep-disordered breathing, with particular emphasis on obstructive sleep apnea.
– We document a structured, scoped multi-publisher search and screening process covering ScienceDirect/Elsevier, SpringerLink, IEEE Xplore, Taylor & Francis Online, and Frontiers Online, resulting in a reviewed corpus of 218 core studies.
– We provide an evolutionary overview of AI for sleep-disordered breathing, using milestone-based research themes to contextualize the recent literature and summarize the field's transition from PSG-centered and reduced-channel diagnostic automation to deep learning, wearable and contactless sensing, trustworthy AI, and emerging LLM-enabled clinical workflows.
– We propose a multidimensional, sleep-medicine-oriented taxonomy that maps AI-for-SDB studies across AI methods, data modalities, acquisition platforms, and clinical applications. The six research domains are not mutually exclusive; rather, they integrate these complementary perspectives.
– We use taxonomy tables and visualization-based evidence maps to identify where current AI-for-SDB research is concentrated and where gaps remain, showing the dominance of diagnosis-, detection-, severity-, and AHI-oriented studies, the increasing use of multimodal and deep-learning methods, and the relative underrepresentation of treatment management, CPAP adherence support, remote monitoring, and clinically validated deployment studies.
– We discuss how AI-based methods can support the transition from conventional one-size-fits-all SDB care toward precision sleep medicine through automated diagnosis, severity estimation, phenotyping, endotyping, treatment planning, CPAP adherence monitoring, remote management, and clinical decision support.
– We examine the emerging role of LLMs in sleep medicine, including patient education, guideline-based question answering, sleep-report interpretation and summarization, clinical documentation, and decision-support workflows, while emphasizing the need for source grounding, clinical validation, and clinician oversight.
– We identify major challenges and future directions, including dataset heterogeneity, external validation, interpretability, fairness, privacy, reproducibility, regulatory oversight, and safe clinical deployment.
– We support transparency and reproducibility by making the review metadata, screening records, RIS files, visualization-support files, and code used to generate the figures available through the project repository: https://github.com/taiduydinh/ai-sdb.

To provide a structured overview of the review scope, Figure 1 summarizes the overall workflow and taxonomy used in this paper. The figure organizes AI for sleep-disordered breathing along four complementary dimensions: acquisition platforms, data modalities, AI and machine-learning methods, and clinical applications. The left panel of Figure 1 presents acquisition platforms together with the data modalities they generate to illustrate their operational relationships. PSG/HSAT systems, wearables and nearables, smartphones, and contactless sensing technologies represent acquisition platforms, whereas physiological signals, audio, imaging and anatomical data, and clinical or questionnaire-based information represent the resulting data modalities. These dimensions are conceptually distinct but are presented together because acquisition platforms determine which types of data can be collected and influence their measurement characteristics and clinical deployment settings. For example, PSG/HSAT systems may collect multiple physiological signals, whereas wearable and nearable devices may collect PPG, SpO_2_, heart-rate or HRV, actigraphy, and audio data. The methodological dimension covers traditional machine learning, ensemble learning and boosting, and neural-network and deep-learning approaches, including CNNs, RNNs, LSTMs, transformers, autoencoders, and LLM-based architectures, together with cross-cutting or advanced approaches such as explainable AI, transfer learning, and clinically deployable trustworthy AI. The clinical-application dimension highlights major use cases including screening, diagnosis, respiratory-event detection, severity or AHI estimation, phenotyping and risk stratification (including cardiovascular and other clinical-risk prediction), CPAP adherence, remote monitoring, and sleep staging. The lower part of the figure summarizes key challenges and opportunities, emphasizing data heterogeneity, external validation, interpretability, privacy and data governance, fairness and bias, safe deployment, scalable screening, precision sleep medicine, multimodal phenotyping, LLM-assisted workflows, remote monitoring, and trustworthy AI translation into clinical practice.

**Figure 1:**
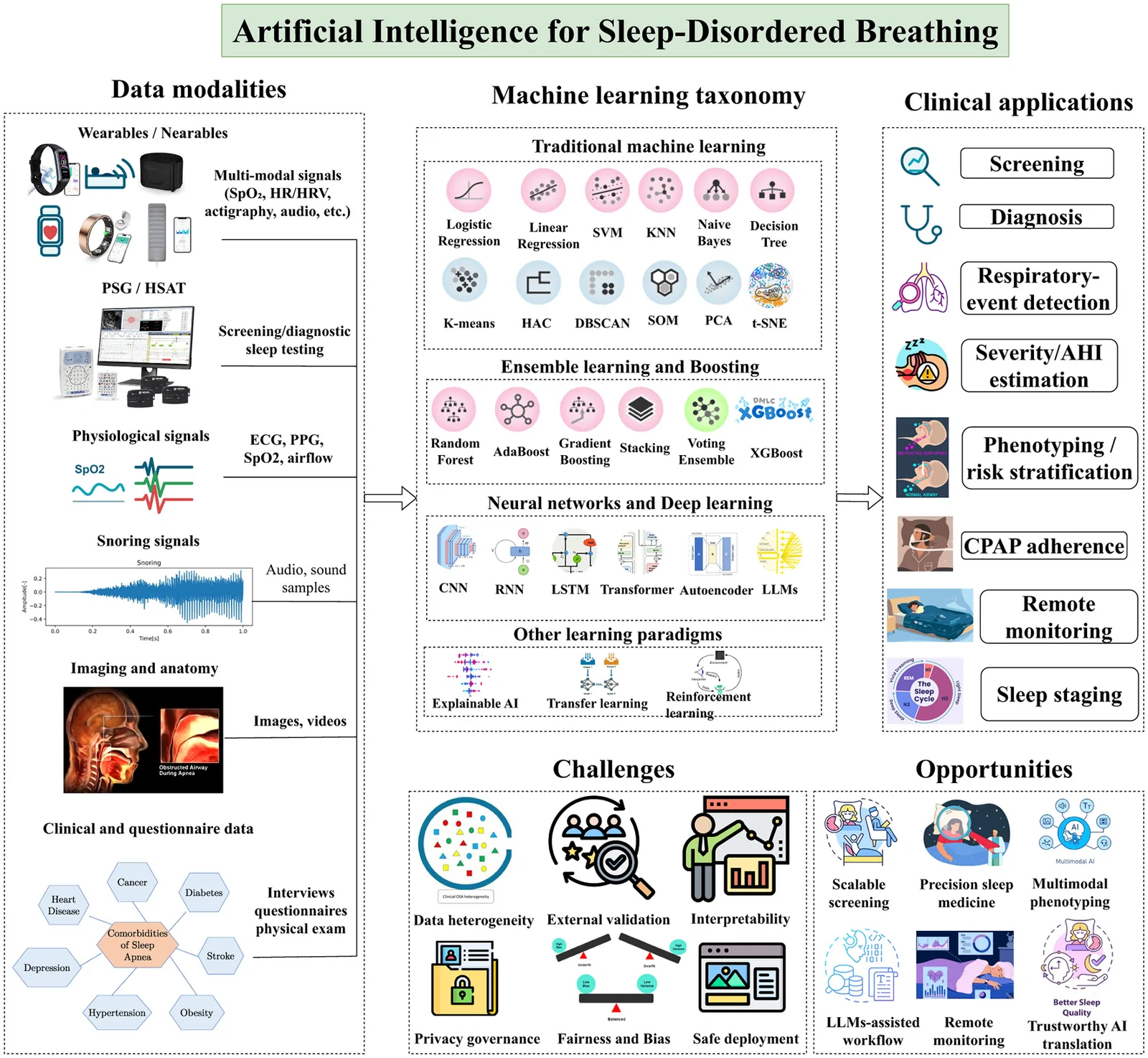
Overview of artificial intelligence for sleep-disordered breathing considered in this review. The workflow summarizes the relationship among major data modalities, acquisition platforms, machine learning taxonomies, and clinical applications, and highlights key challenges and opportunities for clinically deployable AI in sleep medicine.

The remainder of this paper is organized as follows. Section 2 describes the data sources and review strategy. Section 3 presents the historical development of AI for SDB. Section 4 presents the proposed taxonomy of DM and ML technologies for SDB. Section 5 provides a visualization-based synthesis of techniques, tasks, and data modalities in the reviewed corpus. Section 6 discusses key challenges and opportunities. Finally, Section 7 concludes the review and outlines future directions.

## 2 Data sources of the review

This review focuses on studies that apply artificial intelligence (AI), machine learning (ML), deep learning (DL), data mining, and related data-driven techniques to obstructive sleep apnea (OSA) and sleep-disordered breathing (SDB). The primary emphasis is on AI-based diagnosis, screening, detection, classification, prediction, severity estimation, apnea–hypopnea index (AHI) estimation, physiological signal analysis, wearable monitoring, and clinical decision-support systems. Studies on related sleep-respiratory conditions, habitual snoring, sleep-related hypoxemia, and technology-enabled respiratory monitoring were also considered when they directly contributed to OSA/SDB detection, assessment, or management.

Although artificial intelligence applications in sleep-disordered breathing have been investigated for several decades, the systematic literature search in this review focuses on studies published from January 2021 onward. This timeframe was selected to capture recent developments associated with modern AI paradigms, including deep learning architectures, multimodal learning approaches, wearable and nearable sensing technologies, and data-driven methods for clinical sleep monitoring. Earlier studies were considered only to describe the historical evolution of AI applications in SDB and were not included in the systematic evidence synthesis.

The literature search was conducted for studies published from January 2021 onward using a structured, scoped multi-publisher strategy covering ScienceDirect/Elsevier, SpringerLink, IEEE Xplore, Taylor & Francis Online, and Frontiers Online. Although multidisciplinary and biomedical indexing databases provide broad literature coverage, this review did not perform direct systematic searches in PubMed/MEDLINE, Embase, Scopus, or Web of Science. Wiley Online Library, the ACM Digital Library, and pre-print servers such as arXiv and medRxiv were also outside the pre-defined search scope. Individual studies retrieved through the selected publisher platforms may also be indexed in one or more bibliographic databases; however, such indexing overlap does not make publisher-platform searching equivalent to direct database searches. The selected platforms span AI, computer science, biomedical engineering, and clinical sleep research and were used to construct a technically oriented evidence map. Nevertheless, restricting retrieval to five publisher platforms may have omitted relevant work from other publishers, databases, conference collections, and pre-print servers. In addition, the open-access filters applied on SpringerLink and Taylor & Francis Online, as documented in Supplementary Table S1, may have excluded subscription-only articles and introduced access-related selection bias. The resulting 218-study corpus should therefore be interpreted as a scoped evidence map rather than an exhaustive census of AI-for-SDB research; the reported frequencies and trends describe this corpus and should not be assumed to represent prevalence across the entire literature.

The core search concept combined OSA/SDB-related terms with AI/ML/DL-related terms. Platform-specific syntax was retained to reflect the search functionality of each source. ScienceDirect/Elsevier used the task terms “diagnosis” and “screening,” whereas SpringerLink and IEEE Xplore used the truncated forms diagnos* and screen*. The Taylor & Francis Online and Frontiers Online searches used the core OSA and AI concepts without an additional task-oriented clause. The complete publisher-specific search equations were as follows:

ScienceDirect/Elsevier: (“obstructive sleep apnea” OR “sleep apnea” OR “sleep-disordered breathing” OR OSA) AND (“machine learning” OR “deep learning” OR “artificial intelligence”) AND (diagnosis OR screening)

SpringerLink: (“obstructive sleep apnea” OR “sleep apnea” OR “sleep disordered breathin”) AND (“machine learning” OR “deep learning” OR “artificial intelligence”) AND (diagnos* OR screen*)

IEEE Xplore: (“obstructive sleep apnea” OR “sleep apnea” OR “sleep disordered breathing”) AND (“machine learning” OR “deep learning” OR “artificial intelligence”) AND (diagnos* OR screen*)

Taylor & Francis Online: [All: “obstructive sleep apnea”] AND ([All: “machine learning”] OR [All: “deep learning”] OR [All: “artificial intelligence”])

Frontiers Online: “obstructive sleep apnea” AND (“machine learning” OR “deep learning” OR “artificial intelligence”).

Supplementary Table S1 additionally summarizes the corresponding platform-specific filters, journal or section restrictions, and numbers of records identified and assessed.

Studies were screened according to their relevance to OSA/SDB and their use of AI, ML, DL, data mining, or related computational methods. The inclusion criteria were as follows:

– The study addresses OSA, sleep apnea, SDB, snoring, sleep-related respiratory monitoring, or technology-supported management of sleep-respiratory disorders.
– The study applies AI, ML, DL, data mining, signal processing with learning-based models, multimodal AI, wearable-based analytics, or related data-driven methods.
– The study reports a diagnosis, screening, detection, classification, prediction, severity-estimation, AHI-estimation, monitoring, risk-stratification, treatment-support, or clinical decision-support task.
– The study provides sufficient methodological detail regarding data modality, model type, dataset or cohort, validation setting, evaluation metrics, and clinical or technical relevance.

The exclusion criteria were as follows:

– Studies focused only on general sleep quality, insomnia, circadian rhythm, or non-respiratory sleep disorders without clear relevance to OSA/SDB.
– Studies that mention AI, ML, DL, or digital health only conceptually without implementing or evaluating a computational method.
– Pure clinical association studies without AI/ML/DL or data-driven modeling.
– Genetics-only, molecular-biology-only, oncology, endocrinology, or unrelated comorbidity studies unless they directly address AI-based OSA/SDB diagnosis, screening, prediction, or monitoring.
– Editorials, opinions, perspectives, mini reviews, research-topic announcements, and other non-original research articles.
– Studies without sufficient methodological, validation, or performance information.

Across the five publishers, 1,044 records were identified as shown in the Figure 2. The ScienceDirect/Elsevier search returned 516 research articles published between 2021 and 2026. The SpringerLink search returned 153 records after filtering to research articles, open-access content, English language, and the publication period 2021–2026. The IEEE Xplore search returned 131 records; after removing one duplicate PDF, 130 records were assessed. The Taylor & Francis search returned 93 records after applying article-type, open-access, subject-area, and date filters. The Frontiers search returned 151 records after filtering to Health-domain original research articles published between 2021 and 2026 and excluding articles that were part of Research Topics.

**Figure 2:**
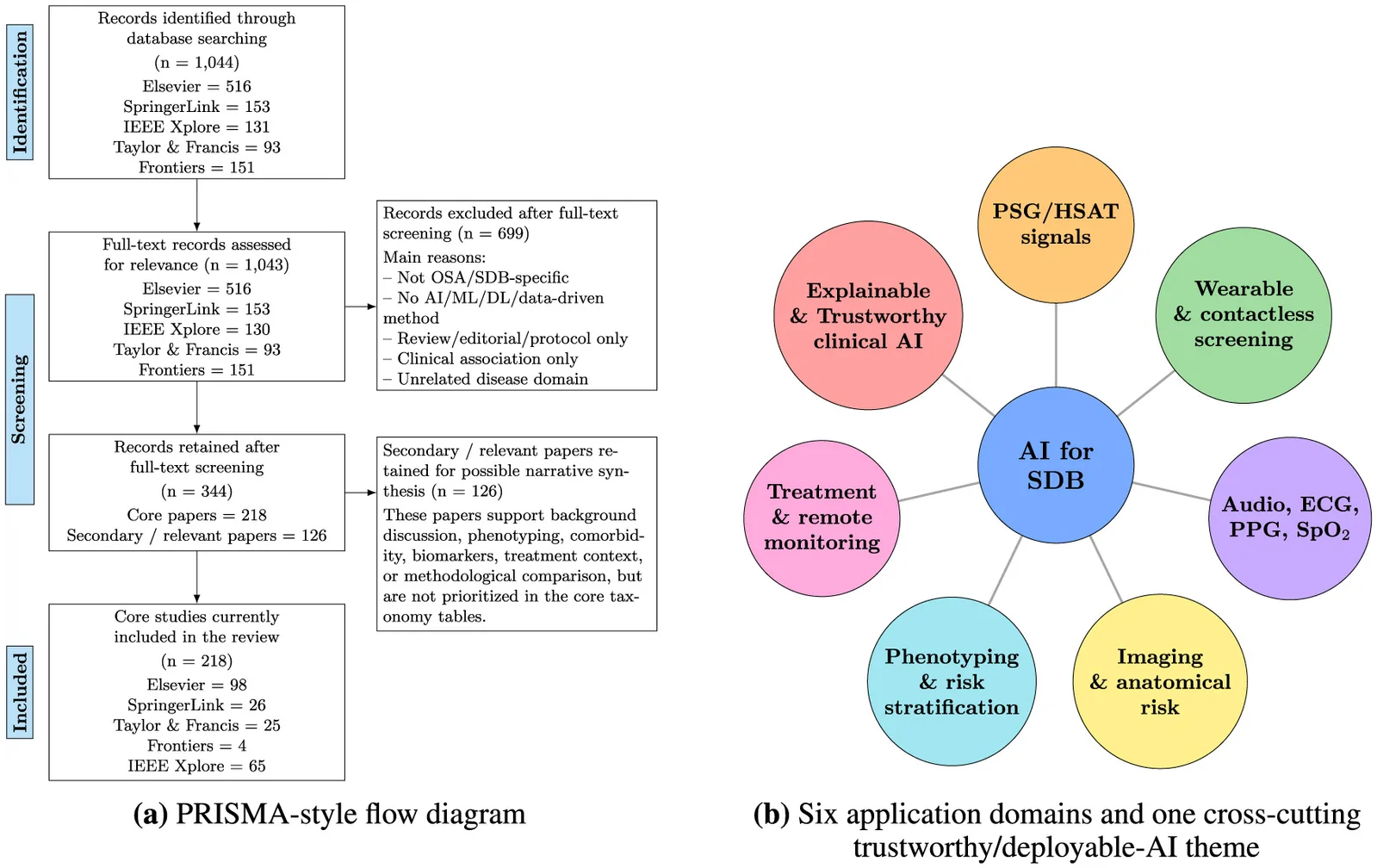
Overview of the review workflow and taxonomy. Panel **(a)** shows the PRISMA-style flow diagram for study identification, screening, and inclusion. Panel **(b)** shows the six primary AI-for-SDB research domains and the cross-cutting explainable, trustworthy, and clinically deployable AI theme considered in this review.

After removal of one duplicate, 1,043 records underwent full-text screening. Of these, 699 records were excluded according to the pre-defined inclusion and exclusion criteria, leaving 344 retained records. Among these, 218 studies met the scope of the core review corpus and directly informed the proposed taxonomy and supplementary evidence tables. The remaining 126 records were retained as contextual studies supporting the background discussion, phenotyping, comorbidity, biomarkers, treatment context, or methodological comparisons; however, they were not included in the core taxonomy tables. The Supplementary Tables summarize each core study by clinical task, data modality, AI or ML technique, dataset/cohort characteristics, reported validation evidence, performance-related findings, and clinical relevance.

## 3 Historical developments in AI for sleep-disordered breathing

The development of AI for SDB, particularly OSA, has progressed through several overlapping phases rather than through a single linear transition. A historical perspective (Figure 3) is useful because it highlights how the field evolved not only in methodological sophistication, but also in clinical scope, data modality, and intended deployment context.

**Figure 3:**
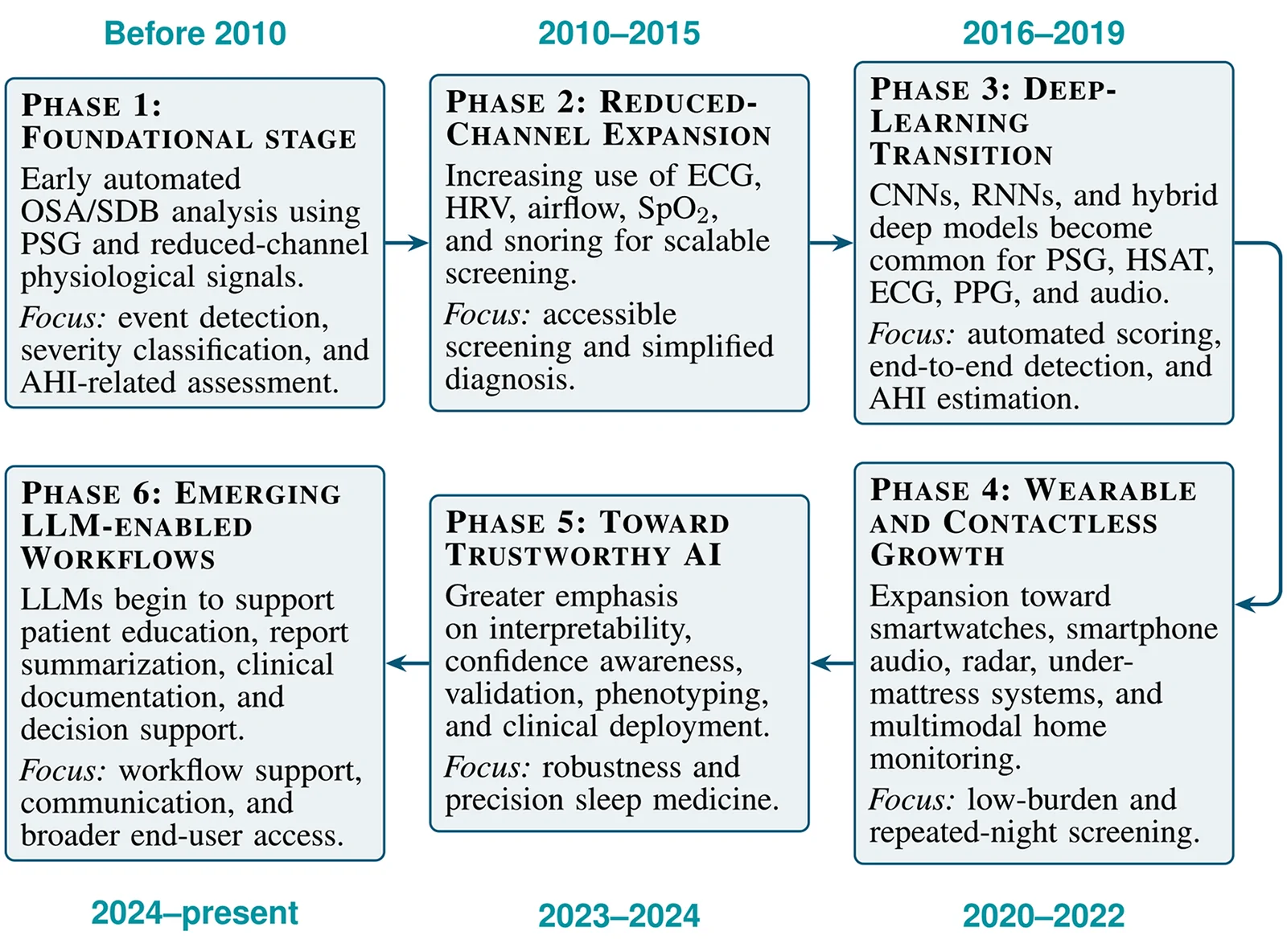
Phase-based evolution of artificial intelligence for sleep-disordered breathing. The timeline highlights representative and partially overlapping research phases, from PSG-centered and reduced-channel diagnostic automation to wearable and contactless screening, multimodal deep learning, trustworthy AI, and emerging LLM-enabled workflows for clinical communication, decision support, and broader end-user access.

The earliest phase focused primarily on *automating diagnosis from conventional physiological recordings*, especially PSG-derived and reduced-channel signals such as airflow, oximetry, ECG, respiratory effort, and snoring-related signals. During this phase, studies relied largely on handcrafted signal-processing features and conventional machine learning classifiers, with major goals including apnea or hypopnea detection, severity classification, and AHI-related assessment. More recent studies in the reviewed corpus still reflect this foundational line of work. Examples include pediatric OSA detection from airflow and oximetry (Jimenez-Garcia et al., 2022), large-scale analysis of respiratory and oximetry signals (Sharma et al., 2022), automated respiratory-event scoring and AHI estimation (Nikkonen et al., 2021), and apnea/hypopnea localization in PSG (Ji et al., 2024).

A second phase was characterized by the *expansion of reduced-channel and home-oriented screening*. As the limitations of in-laboratory PSG became increasingly recognized, more studies explored accessible and lower-burden alternatives based on ECG and HRV (Iwasaki et al., 2021) and wearable multimodal sensing (Zhou et al., 2023). In this phase, the field began to move beyond laboratory-only diagnosis toward scalable screening and triage, with increasing attention to automatic home-based estimation (Retamales et al., 2024) and practical comparison of home-oriented platforms against in-laboratory PSG (Tirachaimongkol et al., 2025).

The third phase marked the *transition toward deep learning*. End-to-end and hybrid deep-learning models, including convolutional, recurrent, transformer-style, and multimodal architectures, became increasingly common for PSG, HSAT, ECG, PPG, audio, and multimodal signals. Representative developments include multimodal hybrid self-supervised sleep analysis (Lee C.-H. et al., 2025), heterogeneity-aware multimodal cross-epoch detection (Tian et al., 2026), fine-grained respiratory-event-boundary detection (Liu Y. et al., 2026), and joint modeling of sleep staging and OSA detection (Wang et al., 2025).

The fourth phase involved a broader *expansion into wearable, contactless, and multimodal SDB assessment*. Research increasingly incorporated wrist-worn consumer technologies (Olsen et al., 2024), sleep-sound analysis (Wang et al., 2022), radar-based sensing (Kwon et al., 2022; Lee K.-T. et al., 2025), and bed-integrated tactile sensing (Zhao T et al., 2024). This period reflects a major conceptual shift: AI for SDB was no longer viewed only as a tool for retrospective diagnosis, but also as a means to support repeated-night monitoring, home-based screening, long-term management, and broader digital sleep-health applications (Olsen et al., 2024; Kwon et al., 2022; Lee K.-T. et al., 2025; Zhao T et al., 2024). Multimodal fusion also became more prominent, integrating physiological signals, wearable data, audio, imaging, and clinical variables (Zhou et al., 2023; Lee C.-H. et al., 2025; Tian et al., 2026).

Most recently, the field has moved toward *trustworthy, clinically deployable, and precision sleep-medicine AI*. In addition to predictive performance, studies have examined confidence-aware decision support (Bark et al., 2023), trustworthy decision-boundary formulations (Chu et al., 2024), and explainable respiratory-signal models (Barroso-Garcìa et al., 2025). At the same time, AI applications have broadened beyond detection and severity classification to include clinical risk prediction (Shi et al., 2023), cardiovascular-risk estimation (Blanchard et al., 2022), phenotyping and endotyping (Pataka et al., 2024; Kuhn et al., 2026), and treatment monitoring and CPAP-related support (Midelet et al., 2023; Wu et al., 2026).

A further *emerging direction* is the use of large language models (LLMs) and clinical natural language processing for patient education, report summarization, clinical documentation, guideline-based question answering, and decision-support workflows. In contrast to signal-based diagnostic models, these applications primarily support communication, workflow efficiency, and knowledge assistance. This phase may also broaden the end users of AI-for-SDB technologies: while earlier systems were mainly designed for sleep specialists, engineers, or trained scorers, LLM-enabled tools may increasingly be used by non-specialist clinicians, digitally less experienced healthcare providers, and patients seeking understandable explanations of sleep-study results or treatment options. Because this direction is still early in SDB research, it should be regarded as a promising workflow-support and access-broadening extension of AI-enabled sleep-medicine practice rather than a fully mature diagnostic application area.

Taken together, these historical shifts show that AI for SDB has evolved from a relatively narrow signal-classification problem into a broader ecosystem of diagnostic, screening, monitoring, and clinical-support technologies. This progression also mirrors the broader transition in sleep medicine from laboratory-centered testing toward scalable, home-based, multimodal, and personalized care.

## 4 A taxonomy of data mining and machine learning for sleep-disordered breathing

To organize the rapidly expanding literature on AI for SDB, we propose a multidimensional taxonomy covering AI methods, data modalities, acquisition platforms, and clinical applications (Figure 4). These dimensions represent different but interconnected aspects of AI-based SDB systems. In particular, data modalities describe the types of information analyzed by AI models, such as physiological signals, audio, imaging, and clinical variables, whereas acquisition platforms describe how these data are collected, including PSG, HSAT, wearable devices, smartphones, and contactless sensing systems. These perspectives are considered together because acquisition platforms influence the types of data that can be collected, measurement characteristics, and clinical deployment scenarios. For example, PSG systems may collect EEG, ECG, airflow, respiratory effort, and SpO_2_, whereas wearable or nearable devices may collect PPG, SpO_2_, actigraphy, or audio. Consequently, individual studies may be characterized across multiple dimensions and assigned to more than one research domain. The proposed taxonomy is therefore designed as an evidence-mapping framework to characterize how AI methods are applied across different sensing environments, data sources, and clinical objectives, rather than as a strictly hierarchical classification.

**Figure 4:**
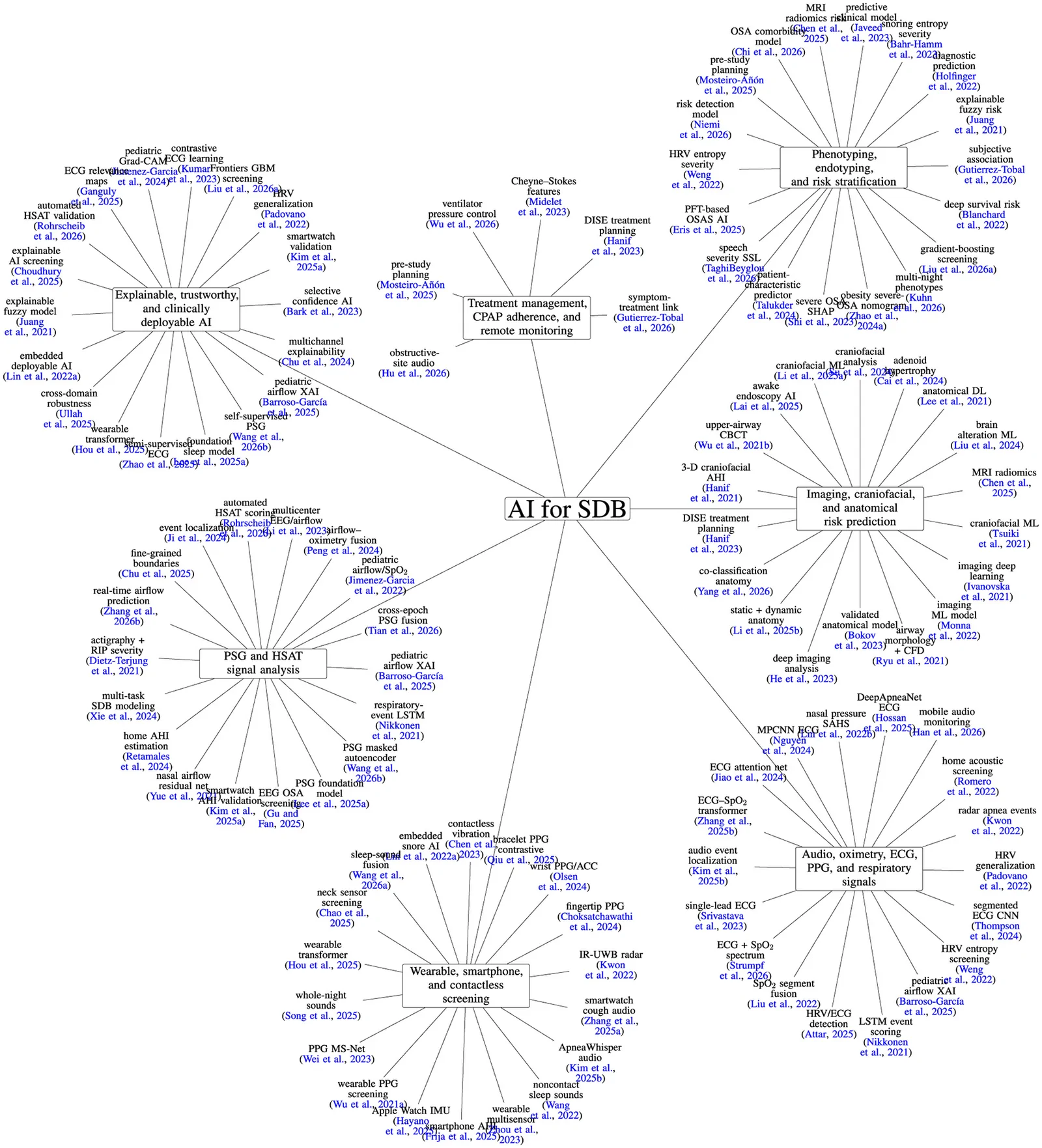
Radial tree-based taxonomy of artificial intelligence methods for the diagnosis and management of sleep-disordered breathing. Six branches correspond to primary AI-for-SDB research domains, integrating complementary perspectives on data modalities, acquisition platforms, and clinical applications, while the explainable, trustworthy, and clinically deployable AI branch is treated as a cross-cutting theme. Citation leaves indicate representative core Elsevier, SpringerLink, Taylor & Francis, Frontiers, and IEEE Xplore studies. Counts refer to multi-label assignments among the screened core papers.

The taxonomy includes six primary AI-for-SDB research domains: PSG/HSAT signal analysis, wearable and contactless screening, audio and physiological-signal-based diagnosis, imaging and anatomical risk prediction, phenotyping and risk stratification, and treatment management or remote monitoring. In addition, we include explainable, trustworthy, and clinically deployable AI as a cross-cutting theme, because interpretability, robustness, validation, and deployment feasibility are relevant across all application domains rather than belonging to only one modality or task.

The six domains were defined by the authors as a clinically oriented, study-level organizing framework and were applied through manual, non-mutually-exclusive coding of the included studies. They were not derived from unsupervised clustering and have not undergone independent expert-consensus assessment, inter-rater reliability testing, or external quantitative validation. Their purpose is therefore descriptive: to make the heterogeneous evidence easier to navigate across sensing environments, data types, methods, and clinical objectives. Accordingly, the number of taxonomy domains is not expected to match the number of clusters produced by a bibliometric term co-occurrence analysis.

The taxonomy is currently populated with the 98 core Elsevier studies, 26 core SpringerLink studies, 25 core Taylor & Francis studies, four core Frontiers studies, and 65 core IEEE Xplore studies identified during screening. Supplementary Tables S2, 3 provide the current multi-publisher evidence map of core AI-for-SDB studies.

Beyond these core taxonomy dimensions, emerging AI paradigms such as large language models (LLMs) represent additional cross-cutting capabilities for AI-enabled sleep medicine. Although most studies in the current core corpus focus on signal-, image-, wearable-, or clinical-variable-based prediction, LLMs represent an emerging cross-cutting direction for AI-enabled sleep medicine. Their role differs from conventional diagnostic models because they are not primarily used to classify respiratory events directly from physiological signals. Instead, they may support workflow-level tasks such as patient education, guideline-based question answering, sleep-study report summarization, clinical documentation, retrieval-augmented decision support, and communication of diagnostic or treatment information to patients and clinicians. In this review, LLM-related applications are therefore discussed as part of clinically deployable AI and decision-support workflows rather than as a separate data-modality group.

### 4.1 Polysomnography and home sleep apnea testing signal analysis

PSG and HSAT remain central data sources for SDB diagnosis because they provide direct or surrogate measurements of airflow, respiratory effort, oxygen saturation, sleep state, arousals, body position, ECG, EEG, EMG, and snoring. Representative PSG/HSAT-oriented studies in the current multi-publisher core corpus include pediatric OSA detection from airflow and oximetry (Jimenez-Garcia et al., 2022), apnea/hypopnea localization using multimodal PSG signals (Ji et al., 2024), and automated respiratory-event scoring and AHI estimation from respiratory and oximetry signals (Nikkonen et al., 2021). The corpus also includes studies that jointly model sleep staging with OSA screening or respiratory-event detection (Huttunen et al., 2023; Wang et al., 2025). Additional approaches in the corpus include multimodal nasal-airflow–SpO_2_ fusion, multicenter sleep-apnea detection from EEG and airflow, large-scale respiratory and oximetry analysis, chin-EMG-based OSA-event screening, fine-grained respiratory-event segmentation, and airflow-based event prediction. The G1 column of Supplementary Table S3 identifies the core studies assigned to this domain.

### 4.2 Wearable, smartphone, and contactless technologies for SDB screening

Wearable, smartphone-based, and contactless technologies are increasingly important for scalable SDB screening. These technologies include audio sensors, chest accelerometers, wearable PPG sensors, ring/bracelet devices, neck-worn sensors, under-mattress systems, breathing-vibration sensors, and smartphone or microphone-based HSAT systems. The current multi-publisher core corpus shows that this direction is moving from laboratory-only diagnosis toward low-burden home screening, repeated-night assessment, and deployable triage tools. Representative examples include contactless breathing-vibration monitoring (Chen et al., 2023), wearable multi-sensor screening (Zhou et al., 2023), and radar-based apnea/hypopnea detection (Kwon et al., 2022). The G2 column of Supplementary Table S3 identifies the core studies assigned to this domain.

### 4.3 Audio, oximetry, ECG, PPG, and respiratory-signal-based diagnosis

Reduced-channel physiological diagnosis is the largest group in the current multi-publisher core corpus evidence map. Included studies use ECG, RR intervals, HRV, ECG-derived respiration, SpO_2_, PPG, airflow, respiratory effort, snoring, tracheal breathing sounds, chin EMG, and multimodal combinations of these signals. The dominant tasks are apnea/hypopnea detection, OSA or OSAHS classification, AHI estimation, event-boundary detection, severity classification, and separation of obstructive, central, and mixed events. Representative examples include ECG attention networks (Jiao et al., 2024), multimodal transformer fusion (Zhang et al., 2025), and audio-transformer event localization (Kim Y. et al., 2025). The G3 column of Supplementary Table S3 identifies the core studies assigned to this domain, including the IEEE Xplore core records.

### 4.4 Imaging, craniofacial, and anatomical analysis for OSA risk prediction

Anatomical factors play an important role in OSA risk, including craniofacial morphology, upper-airway collapsibility, soft-tissue distribution, mandibular position, airway geometry, and endoscopic collapse patterns. Representative studies in the current core corpus include MRI radiomics combined with clinical data (Chen et al., 2025), awake endoscopic video analysis (Lai et al., 2025), and attention-based upper-airway segmentation (Wu et al., 2021). The corpus also includes drug-induced sleep endoscopy scoring, craniofacial photograph analysis, airway morphology and flow-characteristic prediction, 3D craniofacial scan analysis, cephalometric models, and functional-connectivity models for OSA identification and symptom prediction. The G4 column of Supplementary Table S3 identifies the core imaging and anatomical studies assigned to this domain.

### 4.5 Phenotyping, endotyping, and risk stratification for precision sleep medicine

SDB is clinically heterogeneous, and AHI alone cannot capture the full range of symptoms, hypoxic burden, comorbidity, sleep architecture, anatomical risk, and treatment response. AI and data-mining methods can support phenotype discovery, severity stratification, clinical-risk prediction, outcome modeling, and precision sleep medicine. Representative studies in the current core corpus include female-specific ESADA phenotyping (Pataka et al., 2024), clinical decision support using clinical and demographic data (Mosteiro-Añón et al., 2025), and cardiovascular-risk estimation in sleep-apnea patients (Blanchard et al., 2022). The corpus also includes pediatric OSA phenotype networks, clinical/tabular OSA prediction, fuzzy neural-network screening, awake EEG severity classification, EEG-network OSA classification, HRV complexity analysis, entropy-based severity classification, and neuroimaging-based symptom prediction. The G5 column of Supplementary Table S3 identifies the core studies assigned to this domain.

### 4.6 Treatment management, CPAP adherence, and remote monitoring

Compared with diagnostic AI, treatment management and remote monitoring are less represented in the current multi-publisher core corpus. Nevertheless, several papers indicate how AI can support endoscopy-based assessment for treatment planning (Hanif et al., 2023), neural pressure control (Wu et al., 2026), and extraction of clinically relevant features from CPAP airflow data (Midelet et al., 2023). The corpus also includes studies related to treatment-response prediction, residual respiratory-event characterization, and remote monitoring. The G6 column of Supplementary Table S3 identifies the core studies assigned to this domain.

### 4.7 Explainable, trustworthy, and clinically deployable AI for SDB

Explainability, robustness, external validation, limited-label learning, and deployment feasibility appear across many Elsevier core studies. Relevant papers use Grad-CAM, LIME, SHAP, relevance maps, decision-boundary visualization, attention mechanisms, interpretable fusion, semi-supervised learning, self-supervised representation learning, domain generalization, embedded systems, low-power hardware, and AI-driven HSAT validation. Representative studies address explainable ECG detection (Ganguly et al., 2025), selective confidence-aware decision support (Bark et al., 2023), and cross-dataset generalization (Padovano et al., 2022). The G7 column of Supplementary Table S3 identifies the core studies assigned to this cross-cutting domain.

## 5 Visualization-based synthesis of techniques, tasks, and data modalities

To complement the taxonomy tables and narrative synthesis, we further summarized the reviewed core corpus using visualization-based evidence mapping. These figures provide a compact overview of how concepts, AI techniques, clinical tasks, and data modalities are distributed across the included studies. Two complementary types of visualization were used. First, a VOSviewer term co-occurrence map was generated from the titles and abstracts of the 218 core papers to examine the conceptual structure of the corpus based on original bibliographic text. Second, tag-based visualizations were generated from normalized technique, task, and data-modality tags extracted from the screening workbook. These tag-based categories should be interpreted as review-coding categories rather than strictly hierarchical taxonomic levels: the first six categories mainly represent data-modality or application domains, whereas explainable, trustworthy, and clinically deployable AI is treated as a cross-cutting theme that can apply across the other domains. Because papers may contribute to more than one category, the counts shown in the tag-based figures should be interpreted as multi-label tag frequencies rather than mutually exclusive study counts.

For the VOSviewer term co-occurrence analysis, the input RIS file contained the titles and abstracts of all 218 core studies; it did not contain author-keyword fields, and the search equations were not supplied to VOSviewer as visualization terms. VOSviewer treated the titles and abstracts as English-language text data and identified noun phrases using its natural-language-processing pipeline. During automatic noun-phrase unification, most non-alphanumeric characters and accents were removed, uppercase characters were converted to lowercase, and plural noun phrases were converted to singular forms. Full counting was used, meaning that repeated occurrences of a term within the same document contributed separately to its occurrence weight. The initial VOSviewer selection contained 88 terms, each occurring at least 10 times, after the occurrence- and relevance-based term-selection procedure. A controlled thesaurus was then applied to consolidate synonymous abbreviations, full forms, and orthographic variants, yielding the 73 normalized term nodes shown in Figure 5.

**Figure 5:**
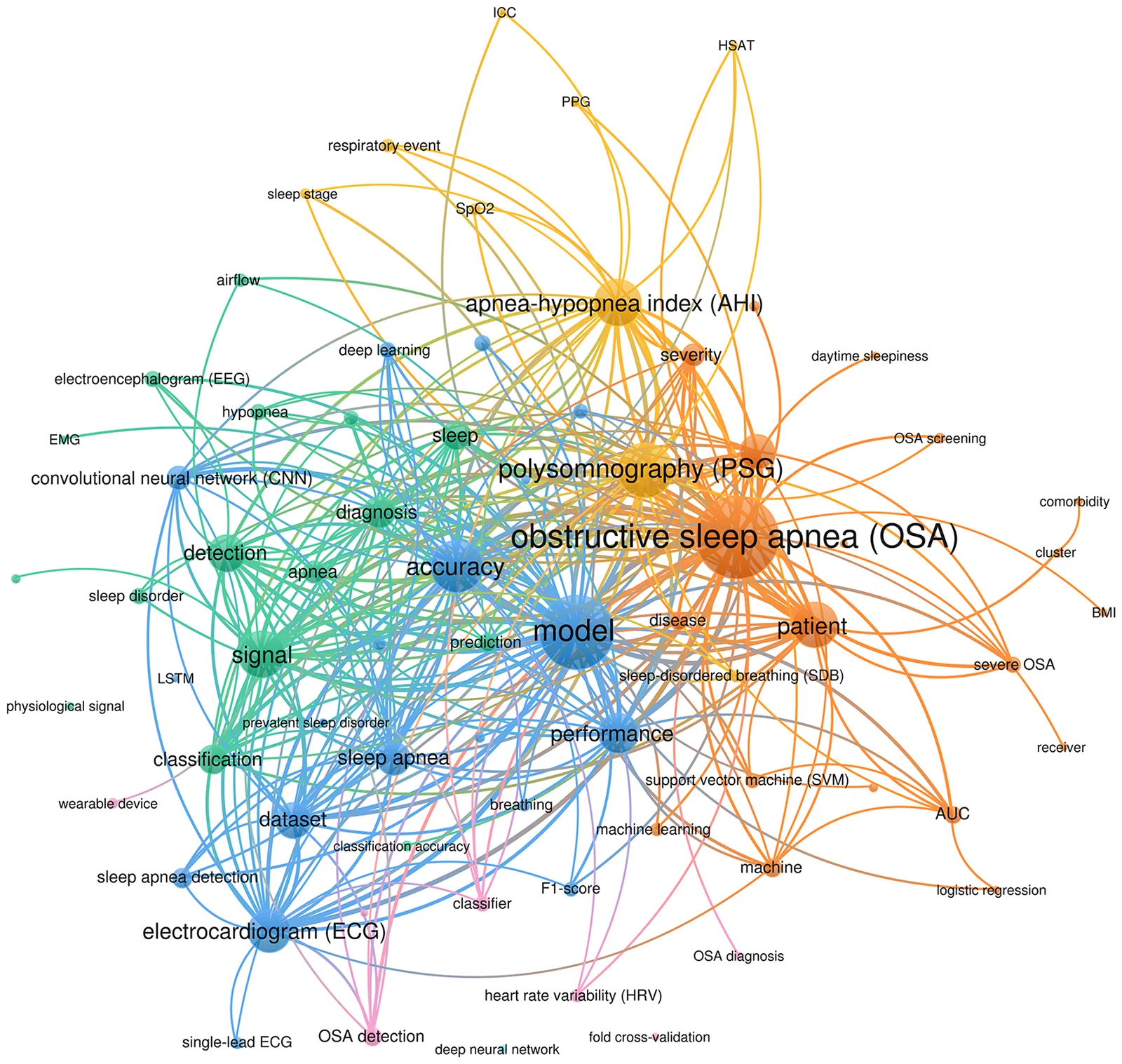
VOSviewer term co-occurrence network generated from the titles and abstracts of the 218 core papers. Synonymous abbreviations, full forms, and orthographic variants were consolidated using a controlled thesaurus-based normalization, yielding 73 normalized term nodes. Node size reflects term occurrence, link strength reflects co-occurrence, and colors indicate clusters of related terms. The four term-level clusters provide a complementary bibliometric view and are not a quantitative validation or one-to-one representation of the six study-level taxonomy domains.

Figure 6 was generated using a different procedure. It was not derived directly from title-and-abstract text and was not produced by VOSviewer. Instead, it was generated from 54 canonical technique tags recorded in the screening workbook. Each paper–technique combination was counted once, and the displayed word size represents the number of included studies assigned the corresponding technique tag.

**Figure 6:**
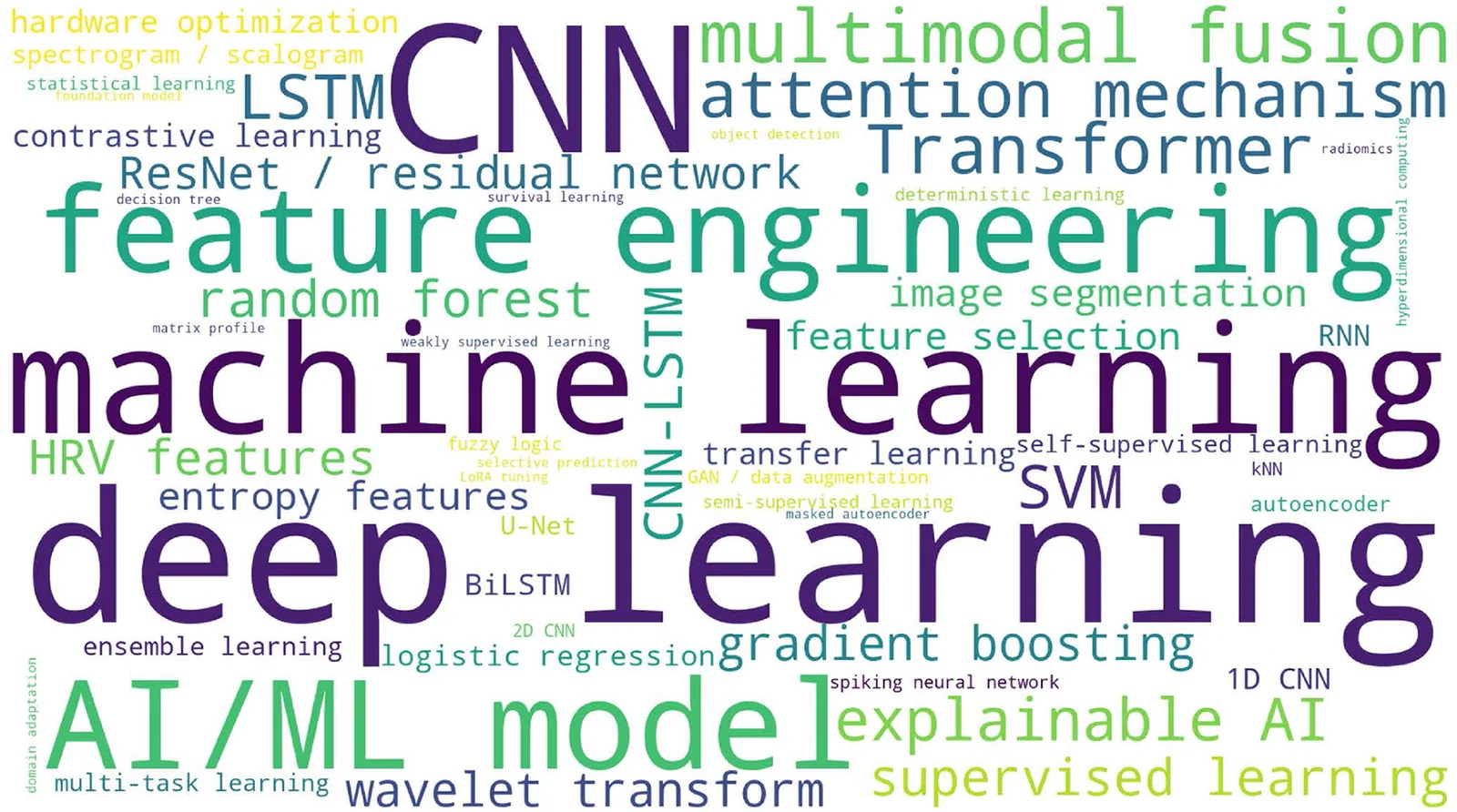
Word-cloud visualization of AI, ML, DL, and data-mining techniques used in the 218 core studies. Larger terms indicate higher frequency in the normalized technique tags.

Because the literature search required both an OSA/SDB-related concept and an AI/ML/DL-related concept, terms overlapping with the search strategy are expected to be prominent in Figures 5, 6. Although the search terms were not inserted directly into either visualization, the search and eligibility criteria condition the composition of the reviewed corpus. These figures should therefore be interpreted as descriptive evidence maps of the included studies and not as independent or unbiased estimates of term or method prevalence across the entire SDB literature.

Figure 5 presents the VOSviewer term co-occurrence network. In this map, each node represents a frequently occurring term, node size reflects term occurrence, links indicate co-occurrence relationships between terms, and colors indicate clusters of related terms detected by VOSviewer.

The largest and most central terms include *obstructive sleep apnea (OSA), model, accuracy, signal, patient, performance, detection, dataset, apnea–hypopnea index (AHI), polysomnography (PSG), diagnosis*, and *classification*. This confirms that the reviewed literature is strongly centered on AI-based detection, diagnostic classification, model evaluation, and PSG/AHI-related assessment. The network also shows a clear physiological-signal and signal-processing region, including terms such as *signal, electrocardiogram (ECG), electroencephalogram (EEG), airflow, hypopnea, deep learning model*, and *convolutional neural network (CNN)*. This reflects the large number of studies using physiological signals and deep-learning architectures for respiratory-event detection and severity estimation.

Another prominent region is organized around clinical and diagnostic concepts, including *obstructive sleep apnea (OSA), patient, severity, severe OSA, OSA screening, BMI, comorbidity*, and *AUC*. This indicates that many studies frame AI models not only as signal classifiers but also as tools for patient-level risk assessment, screening, and severity prediction. A PSG/AHI-centered region includes *apnea–hypopnea index (AHI), polysomnography (PSG), respiratory event, SpO*_2_, *PPG, HSAT*, and *sleep stage*, showing the continued importance of conventional sleep-study indices and physiological measurements in the evaluation of AI-based SDB methods.

The four VOSviewer regions and the six taxonomy domains should be interpreted as complementary rather than numerically corresponding classifications. The VOSviewer regions were generated algorithmically from co-occurring terms in titles and abstracts, whereas the taxonomy was manually assigned at the study level using data-source, acquisition-platform, methodological, and clinical-purpose information obtained from full-text review. A narrative crosswalk illustrates the overlap: the physiological-signal detection region spans PSG/HSAT analysis (G1), wearable or contactless screening (G2), and reduced-channel signal diagnosis (G3); the PSG/AHI-centered region primarily intersects G1 and G3; the model-performance region concerns methodological reporting and the cross-cutting trustworthy/deployable-AI theme (G7); and the patient screening and severity region intersects G2, imaging and anatomical risk prediction (G4), and phenotyping or risk stratification (G5). Treatment management and remote monitoring (G6) comprised only six multi-label assignments in Supplementary Table S2 and did not form a separate term cluster at the minimum occurrence threshold used for Figure 5. Thus, the four regions summarize dominant vocabulary, while the six domains preserve clinically meaningful but sometimes smaller application areas; no one-to-one numerical agreement is expected.

In addition to the title-and-abstract-based VOSviewer analysis, we generated tag-based visualizations from the screening workbook to summarize the techniques, tasks, and data modalities recorded during manual review. Figure 6 provides an overall view of the AI and data-mining techniques used in the core corpus. The most prominent terms include deep learning, convolutional neural networks, machine learning, feature engineering, multimodal fusion, transformer-based models, attention mechanisms, recurrent models, explainable AI, support vector machines, and random forests. This distribution reflects the methodological diversity of recent SDB research. Conventional ML and engineered physiological features remain widely used, particularly for reduced-channel ECG, HRV, oximetry, and clinical-variable models, while DL architectures are increasingly common for signal-based, audio-based, wearable, and multimodal analysis.

Figure 7 shows the temporal distribution of technique terms by publication year. Earlier studies in the reviewed period frequently relied on classical machine learning, CNNs, LSTMs, wavelet-based features, HRV features, and feature-engineering pipelines. More recent studies show greater use of transformer-style models, attention mechanisms, multimodal fusion, self-supervised or representation-learning approaches, and explainable AI. This pattern suggests a gradual transition from feature-engineered single-modality models toward more integrated and representation-driven methods, especially for PSG, ECG, audio, wearable, and multimodal SDB analysis.

**Figure 7:**
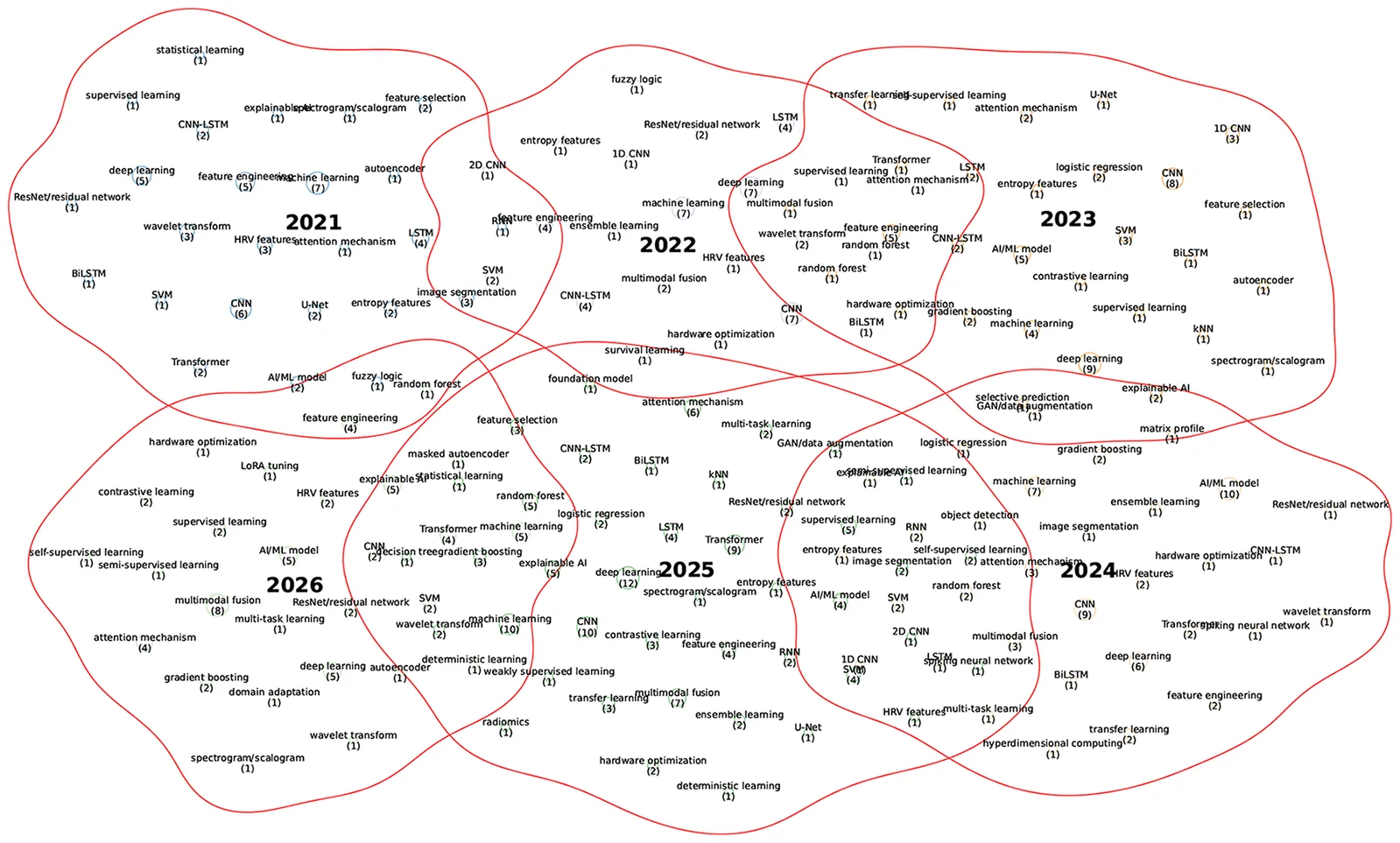
Cluster visualization of AI technique terms by publication year. Each normalized term is plotted at most once per year, and node size reflects its frequency within that year. Terms occurring in multiple years retain separate year-specific instances; because cluster contours can overlap, an instance from a neighboring year may appear within the same visible boundary.

In Figure 7, each normalized technique term is plotted at most once for each publication year in which it occurs. The same term may therefore appear in more than one year-specific cluster. Because the cluster contours can overlap, an instance belonging to a neighboring year may also fall within another year's visible contour, creating the appearance of repetition within a single bubble; this does not represent duplicate records within a year.

For Figures 8–10, each normalized term is represented once within every taxonomy group to which it was assigned, and the number in parentheses indicates its frequency within that group. Because the taxonomy groups are non-mutually exclusive, the same term may be represented separately in several groups. In addition, the cluster contours are allowed to overlap to illustrate shared methodological, task, and data relationships. Consequently, a term instance assigned to a neighboring group may fall within another group's visible contour, creating the appearance that the term is repeated within a single bubble. These apparent repetitions do not represent duplicate term records within the same group.

**Figure 8:**
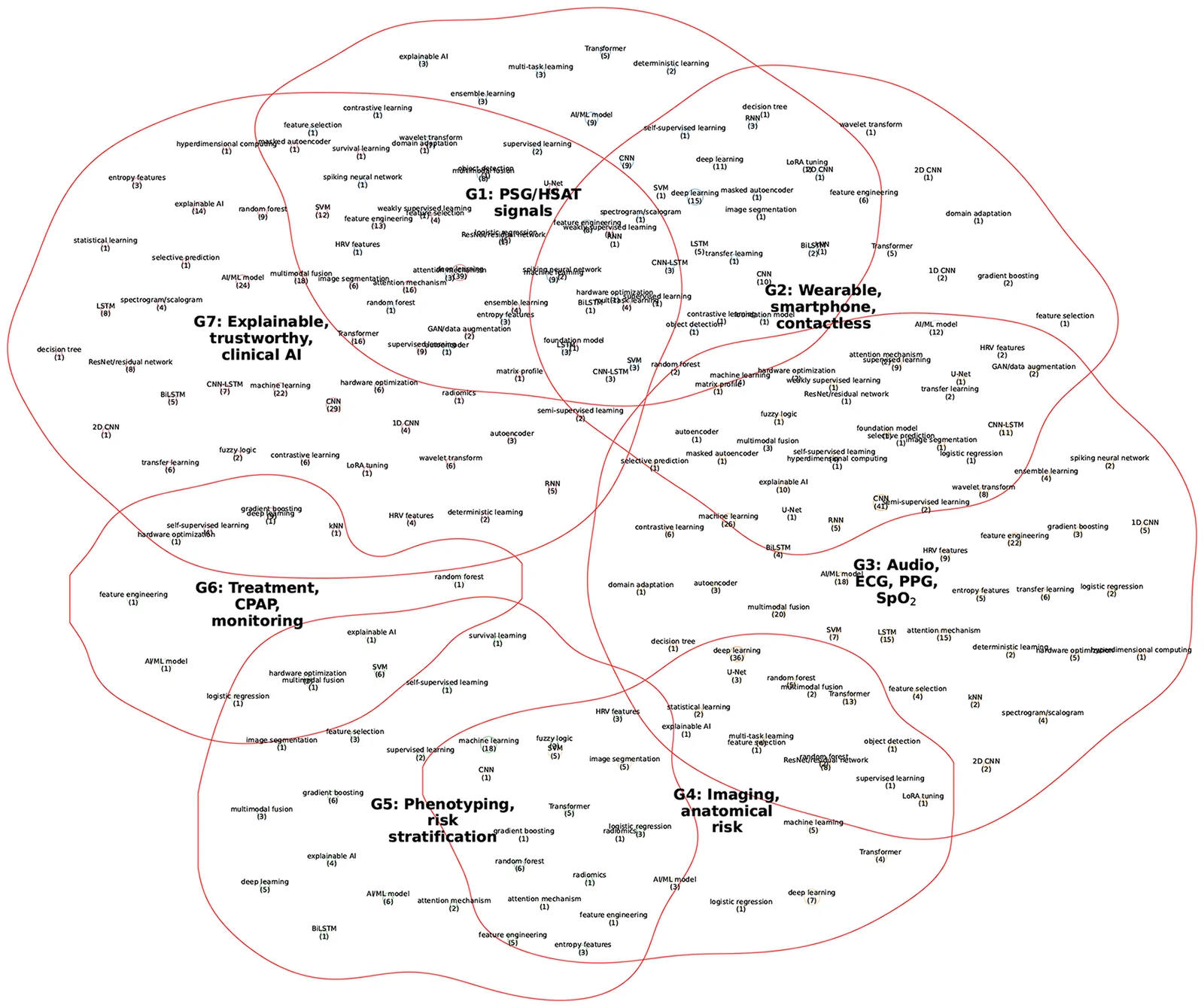
Cluster visualization of technique terms across the six AI-for-SDB application domains and the cross-cutting trustworthy/deployable-AI theme. Each normalized term is plotted at most once per group, with the number in parentheses indicating its within-group frequency. When a term occurs in multiple groups, separate group-specific instances are retained. Because the cluster contours overlap, instances belonging to neighboring groups may appear within the same visible boundary.

**Figure 9:**
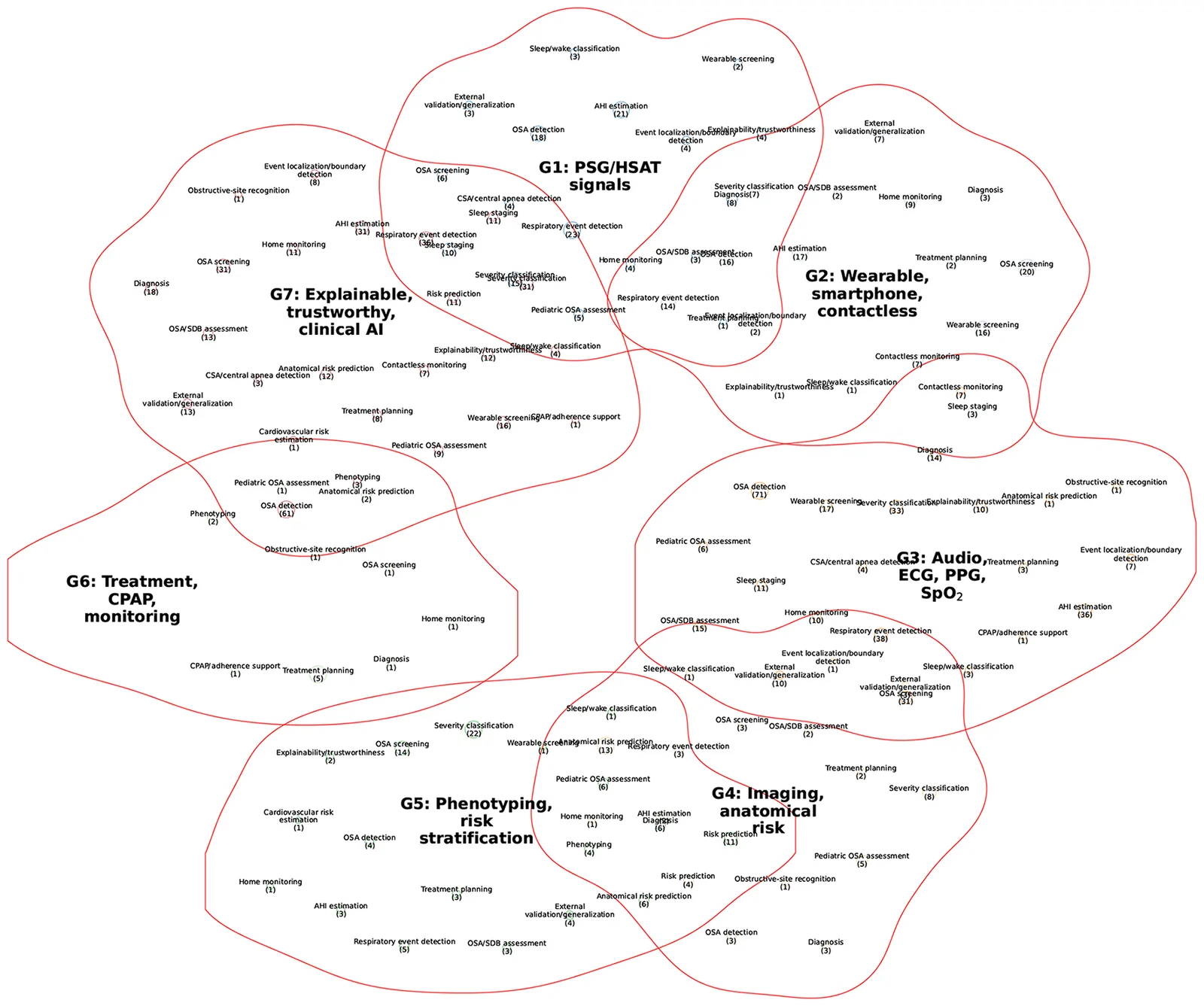
Cluster visualization of clinical and technical task terms across the six AI-for-SDB application domains and the cross-cutting trustworthy/deployable-AI theme. Each normalized task term is plotted at most once per group, with the number in parentheses indicating its within-group frequency. When a task occurs in multiple groups, separate group-specific instances are retained. Because the cluster contours overlap, instances belonging to neighboring groups may appear within the same visible boundary.

**Figure 10:**
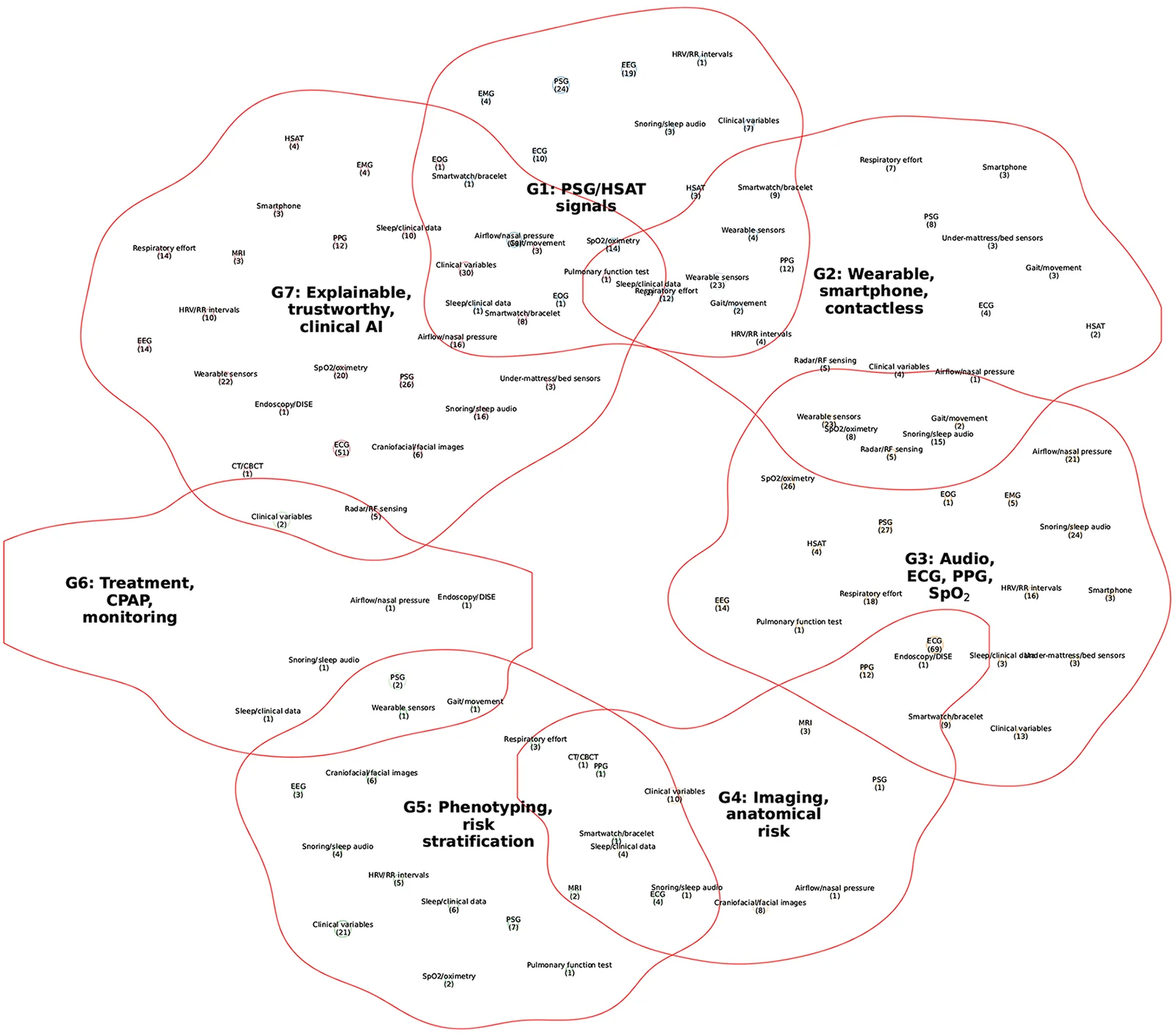
Cluster visualization of data modalities across the six AI-for-SDB application domains and the cross-cutting trustworthy/deployable-AI theme. Each normalized data-modality term is plotted at most once per group, with the number in parentheses indicating its within-group frequency. When a modality occurs in multiple groups, separate group-specific instances are retained. Because the cluster contours overlap, instances belonging to neighboring groups may appear within the same visible boundary.

Figure 8 maps technique terms onto the seven proposed taxonomy groups. The PSG/HSAT group combines deep learning, CNN-based modeling, feature engineering, and multimodal fusion for respiratory-event detection, sleep staging, and AHI estimation. The wearable, smartphone, and contactless group includes deep learning, CNNs, LSTMs, PPG-based modeling, audio analysis, and deployable AI methods. The audio, oximetry, ECG, PPG, and respiratory-signal group is the most methodologically dense, reflecting the large number of reduced-channel and physiological-signal studies. Imaging and anatomical-risk studies are more strongly associated with image segmentation, radiomics, deep learning, SVMs, and transformer-based approaches. Phenotyping and risk-stratification studies rely more heavily on clinical ML models, random forests, gradient boosting, SVMs, and interpretable feature-based methods. The treatment-management group remains comparatively small, indicating that AI for CPAP adherence, remote treatment monitoring, and treatment planning is still underdeveloped relative to diagnosis and screening.

Figure 9 summarizes the distribution of clinical and technical tasks across the seven taxonomy groups. Unlike the taxonomy figure, which presents the conceptual organization of AI-for-SDB research, this task-level map shows which clinical endpoints are repeatedly addressed across different data modalities and which endpoints remain comparatively underrepresented. Repeated task labels across groups indicate that the same task is studied in different technical contexts, for example PSG/HSAT analysis, wearable screening, physiological-signal diagnosis, or imaging-based risk prediction. Across the corpus, the dominant tasks are OSA detection, respiratory-event detection, severity classification, OSA screening, and AHI estimation. PSG/HSAT studies are concentrated around respiratory-event detection, AHI estimation, sleep staging, and severity classification. Wearable and contactless studies emphasize scalable screening, AHI estimation, and home monitoring, whereas the physiological-signal group is dominated by OSA detection, respiratory-event detection, severity classification, and reduced-channel diagnosis. Imaging and craniofacial studies focus mainly on anatomical risk prediction and severity assessment, while phenotyping and risk-stratification studies emphasize risk prediction, comorbidity-aware prediction, and clinically interpretable decision support. In contrast, treatment planning, CPAP adherence support, and remote treatment monitoring remain less frequent, suggesting an important opportunity for future work beyond diagnostic automation.

Figure 10 shows how data modalities are distributed across the taxonomy. ECG, PSG, oximetry, airflow or nasal pressure, respiratory effort, EEG, PPG, wearable sensors, sleep audio, and clinical variables represent the main data sources in the reviewed studies. PSG and HSAT studies are naturally centered on PSG, airflow, EEG, SpO_2_, and respiratory-effort signals. Wearable and contactless studies are characterized by wearable sensors, PPG, smartwatch or bracelet data, sleep audio, accelerometry, and radar or RF sensing. The physiological-signal group is strongly associated with ECG, SpO_2_, PSG, sleep audio, PPG, and HRV/RR intervals. Imaging and anatomical-risk studies rely on craniofacial or facial images, MRI, endoscopy or DISE, airway morphology, and clinical variables. Phenotyping and risk-stratification studies are more dependent on clinical variables, PSG-derived summaries, sleep/clinical data, and comorbidity-related features. These modality patterns reinforce the need for validation strategies that are appropriate to each data source, since signal quality, sensor placement, sampling protocol, labeling practice, and patient population can differ substantially across modalities.

Overall, the visualization-based synthesis supports three observations. First, the literature remains diagnosis-centered: OSA detection, respiratory-event detection, severity classification, screening, and AHI estimation dominate the current task landscape. Second, methodological development is moving from conventional feature-based ML toward multimodal, attention-based, transformer-style, self-supervised, and explainable models, although classical ML remains important for clinical-variable and risk-stratification studies. Third, the data landscape is increasingly heterogeneous, extending from PSG and HSAT signals to ECG, PPG, oximetry, audio, wearable sensors, radar, craniofacial imaging, endoscopy, and clinical records. This heterogeneity creates opportunities for scalable and personalized SDB care, but it also motivates the challenges discussed in the next section, including external validation, generalizability, interpretability, fairness, privacy, and deployment in real-world sleep-medicine workflows.

### 5.1 Methodological quality, validation practices, and performance reporting of AI-for-SDB studies

To complement the taxonomy, we extracted methodological information from all 218 core studies and organized it into a deduplicated evidence table (Supplementary Table S4). The extracted fields include the dataset and cohort size, unit of analysis, train–validation–test strategy, participant- vs. segment-level partitioning, cross-validation method, internal or external validation, reference standard, comparator methods, principal reported performance results, and methodological and reporting limitations identified during extraction, together with data or code availability where reported. This provides a structured descriptive appraisal of methodological and reporting characteristics rather than a formal risk-of-bias rating or quantitative meta-analysis. Formal tools have design-specific scopes: QUADAS-2 was developed for diagnostic-accuracy studies, whereas PROBAST addresses diagnostic and prognostic prediction-model studies (Whiting et al., 2011; Wolff et al., 2019). The present corpus additionally includes event detection and segmentation, sleep staging, unsupervised phenotyping, signal reconstruction, treatment-management, and workflow-oriented studies. Because no single instrument applies uniformly to all of these designs, we did not assign a common pooled risk-of-bias rating across the corpus.

The descriptive appraisal indicates substantial variation in evidentiary strength. Recurrent concerns include small or single-center retrospective cohorts, unclear participant-level separation between training and testing, possible leakage from segment-level partitioning, limited independent or prospective validation, heterogeneous reference standards, and incomplete reporting of calibration, confidence intervals, subgroup performance, and data or code availability. These limitations affect the strength of the conclusions: corpus-level statements about research concentration and methodological trends are more defensible than claims of algorithmic superiority, absolute performance, generalizability, or clinical readiness. High performance obtained only through internal or ambiguously partitioned evaluation should therefore be interpreted cautiously.

Validation strength varied substantially across the corpus. We identified 31 of 218 studies (14.2%) that evaluated a locked model or method in a strict external or cross-dataset test. We counted cross-dataset transfer followed by target-cohort fine-tuning (one study), replication involving retraining on the additional dataset (two studies), and temporal or later-enrolled validation (three studies) separately rather than treating them as equivalent to geographic or cross-dataset external validation. Four studies used a prospective validation cohort; these categories overlap because a prospective cohort may also constitute temporal or external validation. The label “external validation” also did not imply uniform evidentiary strength: external samples ranged from four independent participants in one imaging study to several thousand recordings in large clinical or community cohorts (Yoo et al., 2026; Retamales et al., 2024; Liu et al., 2022). Accordingly, validation design, cohort independence, sample size, and recruitment setting should be considered together.

The unit of data partitioning was another important source of variation. For signal-, audio-, and image-based studies, random allocation of epochs, windows, or events can place observations from the same participant in both training and test sets, leading to optimistic estimates if subject-level separation is not enforced. A direct illustration is provided by an audio-based study in which the same model achieved 88.32% accuracy and an F1-score of 86.28% with a subject-dependent segment split, but 68.55% accuracy and an F1-score of 60.29% with a subject-independent split (Song et al., 2025). Likewise, a cross-database analysis of HRV-based apnea detection found that external-validation accuracy, sensitivity, and specificity were 20–40% lower than epoch-wise cross-validation results (Padovano et al., 2022). These findings support participant- or recording-level partitioning, explicit reporting of the allocation unit, and external testing whenever the intended use involves previously unseen patients or devices.

Reported performance must also be interpreted in relation to the clinical endpoint. Event-level detection studies commonly reported sensitivity, specificity, F1-score, AUROC, and AUPRC, whereas AHI-estimation studies additionally used correlation, mean error, or agreement analyses; patient-level screening studies often reported threshold-specific AUC, sensitivity, and specificity. For example, a single-effort-belt model trained on MGH data yielded an external SHHS AUROC of 0.92 but an AUPRC of 0.56, illustrating how class prevalence and the selected metric affect interpretation (Nassi et al., 2022). A model trained and validated on SHHS and tested on unseen MESA and MrOS cohorts reported an event-level AUROC of 0.87, an AUPRC of 0.90, and an F1-score of approximately 0.72 for AHI-severity classification using abdominal movement and SpO_2_ (Retamales et al., 2024). Another SpO_2_ study trained on 500 individuals and evaluated 8,131 individuals across held-out and independent datasets, reporting 84.3% segment-classification accuracy and a mean accuracy of 88.95% for identifying SDB at AHI ≥15 (Liu et al., 2022). These values are informative within their respective tasks and evaluation settings, but they should not be combined into an overall ranking.

Within-study comparisons using the same data and split provide more interpretable evidence about relative approaches. In a large clinical-variable study, ANN and random-forest models achieved AUCs of 0.70 in a prospective SAGIC testing cohort, similar to STOP-BANG (0.71), while the ANN and STOP-BANG both achieved an AUC of 0.72 in the prospective SHHS cohort (Holfinger et al., 2022). In a two-site clinical and biochemical study, logistic regression outperformed the other evaluated algorithms and achieved an AUC of 0.732 and an F1-score of 0.788 in the site-separated test cohort (Huang et al., 2024). External performance also depended on population and task: a pediatric craniofacial model decreased from an AUC of 0.812 in development to 0.763 in an independent-institution cohort (Su et al., 2024), whereas a prospectively validated commercial smartwatch algorithm achieved an AUC of 0.931 for moderate-or-greater OSA against physician-scored PSG in 53 participants (Kim D. et al., 2025). A questionnaire-based model retained an AUC of 0.91 (95% CI: 0.88–0.94) in a later prospective temporal cohort of 189 participants (Liu T. et al., 2026). Collectively, these findings do not establish universal superiority of deep learning over conventional machine learning; they instead show that comparative conclusions are most reliable when models are evaluated on the same cohort, target, reference standard, and participant-separated test set.

Overall, the evidence supports a validation hierarchy that prioritizes transparent participant-level separation, locked-model testing in independent cohorts, calibration and agreement analyses where appropriate, and prospective evaluation in the intended clinical workflow. Future AI-for-SDB studies should report cohort flow, pre-processing and split procedures, hyperparameter-tuning boundaries, reference scoring, confidence intervals, subgroup performance, and data or code availability. Such reporting would make performance comparisons more reproducible and would improve assessment of generalizability, fairness, and readiness for clinical deployment.

## 6 Challenges and opportunities

### 6.1 Challenges

*Data heterogeneity* is a major challenge for AI-based SDB diagnosis and management. PSG, HSAT, oximetry, ECG, PPG, audio, wearable, imaging, and clinical-text data differ in sampling frequency, sensor quality, annotation protocol, patient population, and clinical context. Models trained on one device, sleep laboratory, or scoring protocol may not generalize to another. Studies comparing PSG signal combinations (Huttunen et al., 2023) and using heterogeneity-aware multimodal cross-epoch modeling (Tian et al., 2026) show the potential of integration, but they also illustrate why SDB-specific validation across heterogeneous devices and cohorts is essential.

*Clinical validation and generalizability* remain central barriers. Many AI models report strong internal performance but lack external validation across independent cohorts, device types, demographic groups, and clinical settings. For SDB technologies, validation should include not only standard classification metrics but also agreement with expert scoring, diagnostic calibration, robustness to artifacts, and clinical usefulness in screening or treatment workflows. Research on HRV-based model transfer shows that real-world generalization should be treated as a separate evaluation target rather than assumed from internal cross-validation (Padovano et al., 2022). Comparisons of AI-enabled home-testing platforms with in-laboratory PSG further illustrate the importance of independent clinical validation (Tirachaimongkol et al., 2025).

*Interpretability* is especially important because sleep clinicians must understand model outputs before using them for diagnosis or treatment decisions. Black-box models may achieve high accuracy but fail to show which respiratory events, oxygen desaturation patterns, sleep stages, anatomical features, or behavioral factors influenced the prediction. Explainable ECG models (Ganguly et al., 2025) and airflow-based models (Barroso-Garcìa et al., 2025) demonstrate how relevance maps, attention mechanisms, or explanation layers can support clinical trust, error analysis, and regulatory evaluation.

*Privacy and data governance* are important because sleep data may be collected in laboratories, homes, mobile applications, wearable devices, and cloud platforms. These data can contain sensitive physiological, behavioral, and clinical information. Home-based AHI estimation (Retamales et al., 2024) and AI-enabled HSAT platforms (Tirachaimongkol et al., 2025) therefore require secure data handling, transparent device validation, careful model updating, and clear boundaries between screening support and definitive clinical diagnosis.

*LLM safety and reliability* are emerging concerns. LLMs may help explain sleep-study reports, answer patient questions, or support documentation, but they may also generate inaccurate or unsupported recommendations. Their use in SDB care requires source grounding, guideline alignment, uncertainty communication, clinician oversight, and careful evaluation in realistic clinical scenarios. Because most core studies in the present taxonomy focus on diagnostic AI rather than LLM-based clinical deployment, LLM applications should be treated as an emerging and separately validated layer of sleep-medicine decision support.

### 6.2 Opportunities

*Scalable screening* is one of the most immediate opportunities. Reduced-channel signals, wearables, smartphones, and contactless sensors may help identify individuals at risk of SDB before formal sleep testing. AI can support triage by estimating the probability of clinically significant OSA and prioritizing patients for PSG, HSAT, or specialist evaluation. Wearable multi-sensor systems (Zhou et al., 2023), smartwatch-based PPG screening (Chen et al., 2021), and radar-based apnea/hypopnea detection (Kwon et al., 2022) illustrate the range of low-burden screening pathways now being evaluated.

*Precision sleep medicine* is a major opportunity. Multimodal AI can integrate physiological signals, symptoms, comorbidities, demographics, imaging, and treatment data to identify phenotypes and predict patient-specific outcomes. Such approaches may help move beyond AHI alone and support personalized treatment selection, risk stratification, and follow-up planning based on treatable traits. Current examples include interpretable severe-OSA prediction (Shi et al., 2023), obesity-related severe-OSA nomograms (Zhao A et al., 2024), and deep survival modeling of cardiovascular risk in sleep-apnea patients (Blanchard et al., 2022).

*Remote monitoring and adaptive management* can improve long-term SDB care. AI models can analyze CPAP device logs, residual events, usage patterns, wearable data, and patient feedback to identify adherence problems, treatment failure, or the need for clinical intervention. This may reduce the burden on sleep clinics while improving continuity of care. Early evidence from CPAP-flow feature extraction (Midelet et al., 2023) and neural pressure-control modeling (Wu et al., 2026) suggests how treatment-device data may be transformed into adaptive management signals.

*Large language models and clinical NLP* may support parts of the SDB care pathway that are not primarily signal-classification tasks. As introduced in the taxonomy discussion, potential use cases include patient education, guideline-based question answering, sleep-study report summarization, clinical documentation, retrieval-augmented decision support, and explanation of diagnostic or treatment information. These applications may help clinicians manage increasing volumes of sleep-study data and improve communication with patients. However, LLMs should be treated cautiously in sleep medicine because they can generate unsupported or inaccurate statements, may fail to follow clinical guidelines, and require source grounding, validation, privacy protection, and clinician oversight before clinical deployment.

*Clinically deployable and trustworthy AI* represents the long-term goal. Future systems should be transparent, externally validated, privacy-preserving, fair across demographic groups, robust to real-world artifacts, and integrated into sleep-medicine workflows. Stronger reporting standards, shared benchmarks, and open validation datasets will be essential for translating AI-based SDB technologies from research prototypes to clinical practice. Multimodal self-supervised sleep modeling (Lee C.-H. et al., 2025) and multichannel PSG reconstruction frameworks (Wang et al., 2026) may provide useful foundations for more robust sleep-event monitoring, but they still require careful clinical validation before deployment.

### 6.3 Limitations of this review

This review has several limitations. First, the pre-defined search covered five publisher platforms and did not include direct searches of PubMed/MEDLINE, Embase, Scopus, Web of Science, Wiley Online Library, the ACM Digital Library, arXiv, or medRxiv. Open-access filtering on two platforms may also have introduced access-related selection bias. Consequently, relevant studies may have been missed, and the observed distributions should be interpreted as properties of the 218-study corpus rather than exhaustive estimates for the field.

Second, the heterogeneous corpus could not be assessed uniformly with a single formal risk-of-bias instrument. The structured appraisal in Supplementary Table S4 documents validation design, partitioning, reference standards, comparators, performance reporting, and identifiable methodological limitations, but it does not replace design-specific QUADAS-2, PROBAST, or other risk-of-bias assessments. Variable cohort quality, possible leakage, limited external validation, and incomplete reporting mean that strong internally reported performance cannot by itself establish comparative superiority or clinical readiness.

Third, the six-domain taxonomy is an author-developed, non-mutually-exclusive evidence-mapping framework rather than an independently validated classification. The four VOSviewer clusters operate at the term level and provide a complementary bibliometric perspective, not quantitative confirmation of the six study-level domains. Future work could assess the framework through independent expert consensus, inter-rater agreement, alternative clustering and sensitivity analyses, and application to an expanded external corpus.

## 7 Conclusion

This review synthesized recent advances in data mining, machine learning, deep learning, and large language models for the diagnosis and management of sleep-disordered breathing, with particular emphasis on obstructive sleep apnea. We organized the literature using a sleep-medicine-oriented taxonomy covering PSG and HSAT signal analysis, wearable and contactless screening, audio and physiological-signal-based diagnosis, imaging and anatomical risk prediction, phenotyping and endotyping, treatment management, CPAP adherence, remote monitoring, and clinically deployable explainable AI.

The reviewed studies show that AI-based methods are expanding SDB care beyond conventional laboratory-based diagnosis. This development aligns with the broader shift away from one-size-fits-all management toward treatable-trait and precision approaches (Lisik and Zou, 2025). Deep learning, multimodal fusion, wearable sensing, contactless monitoring, imaging analytics, and interpretable machine learning are enabling new approaches for screening, event detection, severity estimation, risk stratification, and personalized management.

Future progress will depend on rigorous external validation, transparent reporting, reproducible benchmarking, clinically meaningful evaluation metrics, privacy-preserving data governance, fairness across demographic groups, and safe integration into sleep-medicine workflows. As SDB care increasingly moves toward home-based monitoring, multimodal digital phenotyping, and personalized management, trustworthy AI and carefully validated LLM-based tools may play an important role in improving accessibility, scalability, and continuity of care.

Accordingly, the findings of this review should be interpreted as a scoped evidence map rather than an exhaustive field census, formal comparative-effectiveness assessment, or validated ranking of AI approaches.

## References

[B1] Bark B. Nam B. Kim I. Y. (2023). SelANet: decision-assisting selective sleep apnea detection based on confidence score. BMC Med. Info. Decision Making 23:190. doi: 10.1186/s12911-023-02292-3 PMC10514955 37735681

[B2] Barroso-Garcìa V. Vaquerizo-Villar F. Gutiérrez-Tobal G. C. Dayyat E. Gozal D. Leppänen T. . (2025). An explainable deep-learning approach to detect pediatric sleep apnea from single-channel airflow. IEEE J. Transl. Eng. Health Med. 13, 517–531. doi: 10.1109/JTEHM.2025.3625388 41502786 PMC12772987

[B3] Blanchard M. Feuilloy M. Sabil A. Gervès-Pinquié C. Gagnadoux F. Girault J.-M. (2022). A deep survival learning approach for cardiovascular risk estimation in patients with sleep apnea. IEEE Access 10, 133468–133478. doi: 10.1109/ACCESS.2022.3231743

[B4] Chen T. Zhang J. Xu Z. Redmond S. J. Lovell N. H. Liu G. Wang C. (2024). Energy-efficient sleep apnea detection using a hyperdimensional computing framework based on wearable bracelet photoplethysmography. IEEE Trans. Biomed. Eng. 71, 2483–2494. doi: 10.1109/TBME.2024.3377270 38483799

[B5] Chen Y. Ma G. Zhang M. Yang S. Yan J. Zhang Z. . (2023). Contactless screening for sleep apnea with breathing vibration signals based on modified u-net. Sleep Med. 107, 187–195. doi: 10.1016/j.sleep.2023.04.030 37201224

[B6] Chen Y. Wang W. Guo Y. Zhang H. Chen Y. Xie L. (2021). A single-center validation of the accuracy of a photoplethysmography-based smartwatch for screening obstructive sleep apnea. Nat. Sci. Sleep 13, 1533–1544. doi: 10.2147/NSS.S323286 34557047 PMC8453177

[B7] Chen Y. Xiao H. Huang M. Zheng Y. Dong X. Chen G. (2025). Predictive modeling of obstructive sleep apnea using pharyngeal magnetic resonance imaging radiomics and clinical data. J. Clin. Sleep Med. 21, 1363–1369. doi: 10.5664/jcsm.11706 40190111 PMC12320681

[B8] Chu R. Wei J. Lu W. Dong C. Chen Y. (2024). Mfs-dbf: a trustworthy multichannel feature sieve and decision boundary formulation system for obstructive sleep apnea detection. Comput. Biol. Med. 179:108842. doi: 10.1016/j.compbiomed.2024.108842 38996552

[B9] Ganguly B. Dasgupta R. Dey D. (2025). A residual deep learning framework for sleep apnea diagnosis from single lead electrocardiogram signals: an explainable artificial intelligence approach. Eng. Appl. Artificial Intelligence. 148:110481. doi: 10.1016/j.engappai.2025.110481

[B10] Hanif U. Kiaer E. K. Capasso R. Liu S. Y. Mignot E. J. M. Sorensen H. B. D. . (2023). Automatic scoring of drug-induced sleep endoscopy for obstructive sleep apnea using deep learning. Sleep Med. 102, 19–29. doi: 10.1016/j.sleep.2022.12.015 36587544

[B11] Hanif U. Leary E. Schneider L. Paulsen R. Morse A. M. Blackman A. . (2021). Estimation of apnea-hypopnea index using deep learning on 3-d craniofacial scans. IEEE J. Biomed. Health Info. 25, 4185–4194. doi: 10.1109/JBHI.2021.3078127 33961569

[B12] Holfinger S. J. Lyons M. M. Keenan B. T. Mazzotti D. R. Mindel J. Maislin G. . (2022). Diagnostic performance of machine learning-derived osa prediction tools in large clinical and community-based samples. Chest 161, 807–817. doi: 10.1016/j.chest.2021.10.023 PMC8941600 34717928

[B13] Hu X. Fang R. Luo H. Luo J. Chen C. Chen W. (2026). A 1D snoring waveform and 2d composite acoustic feature graph-based multi-modal fusion network for obstructive sites recognition. IEEE J. Biomed. Health Informat. 1–12. doi: 10.1109/JBHI.2026.3670208 41790812

[B14] Huang J. Zhuang J. Zheng H. Yao L. Chen Q. Wang J. Fan C. (2024). A machine learning prediction model of adult obstructive sleep apnea based on systematically evaluated common clinical biochemical indicators. Nat. Sci. Sleep 16, 413–428. doi: 10.2147/NSS.S453794 PMC11063111 38699466

[B15] Huttunen R. Leppänen T. Duce B. Arnardottir E. S. Nikkonen S. Myllymaa S. . (2023). A comparison of signal combinations for deep learning-based simultaneous sleep staging and respiratory event detection. IEEE Trans. Biomed. Eng. 70, 1704–1714. doi: 10.1109/TBME.2022.3225268 36441886

[B16] Iwasaki A. Nakayama C. Fujiwara K. Sumi Y. Matsuo M. Kano M. . (2021). Screening of sleep apnea based on heart rate variability and long short-term memory. Sleep Breathing 25, 1821–1829. doi: 10.1007/s11325-020-02249-0 PMC8590683 33423183

[B17] Ji Y. Chen D. Zuo Y. Gao T. Tang Y. (2024). Accurate apnea and hypopnea localization in psg with multi-scale object detection via dual-modal feature learning. Biomed. Signal Proc. Control 89:105717. doi: 10.1016/j.bspc.2023.105717

[B18] Jiao M. Song C. Xian X. Yang S. Liu F. (2024). Deep attention networks with multi-temporal information fusion for sleep apnea detection. IEEE Open J. Eng. Med. Biol. 5, 792–802. doi: 10.1109/OJEMB.2024.3405666 PMC11505982 39464487

[B19] Jimenez-Garcia J. Garcia M. Gutierrez-Tobal G. C. Kheirandish-Gozal L. Vaquerizo-Villar F. Alvarez D. . (2022). A 2D convolutional neural network to detect sleep apnea in children using airflow and oximetry. Comput. Biol. Med. 147:105784. doi: 10.1016/j.compbiomed.2022.105784 35797888

[B20] Kara M. Lakner Z. Tamás L. Molnár V. (2025). Artificial intelligence in the diagnosis of obstructive sleep apnea: a scoping review. Eur. Archiv. Oto-Rhino-Laryngol. 282, 4967–4978. doi: 10.1007/s00405-025-09377-x PMC12518446 40220178

[B21] Kim D. Han J. Y. Jung H. Song D. Y. Lee C. Ryu G. . (2025). AI-enhanced smartwatch AHI estimation and AI-scored polysomnography for obstructive sleep apnea: real-world validation. Nat. Sci. Sleep 17, 2297–2307. doi: 10.2147/NSS.S540460 41018852 PMC12474664

[B22] Kim Y. Kim M. Shin J. Ko M. (2025). Apneawhisper: transformer-based audio segmentation for fine-grained non-invasive sleep apnea detection. Nat. Sci. Sleep 17, 2455–2468. doi: 10.2147/NSS.S553774 PMC12506788 41069504

[B23] Kuhn J. Schiphorst L. R. B. Wulterkens B. M. Asin J. Overeem S. van Gilst M. M. . (2026). Sleep apnea phenotypes based on multi-night assessment of sleep perception. Behav. Sleep Med. 24, 430–442. doi: 10.1080/15402002.2026.2630936 41689504

[B24] Kwon H. B. Son D. Lee D. Yoon H. Lee M. H. Lee Y. J. . (2022). Hybrid cnn-lstm network for real-time apnea-hypopnea event detection based on ir-uwb radar. IEEE Access 10, 17556–17564. doi: 10.1109/ACCESS.2021.3081747

[B25] Lai W.-S. Li T.-W. Kuo C.-F. J. Liu S.-C. (2025). AI-assisted awake endoscopic video analysis for obstructive sleep apnea detection. Nat. Sci. Sleep 17, 2807–2818. doi: 10.2147/NSS.S559788 41200474 PMC12587850

[B26] Lee C.-H. Kim H. Yoon B. C. Kim D.-J. (2025). Toward foundational model for sleep analysis using a multimodal hybrid-self-supervised learning framework. IEEE Transac. Cybernetics 55, 5619–5632. doi: 10.1109/TCYB.2025.3603608 40924530

[B27] Lee K.-T. Liu W.-T. Lin Y.-C. Chen Z. Ho Y.-H. Huang Y.-W. . (2025). Utilizing a wireless radar framework in combination with deep learning approaches to evaluate obstructive sleep apnea severity in home-setting environments. J. Multidiscipl. Healthcare 18, 381–393. doi: 10.2147/JMDH.S486261 PMC11771517 39872870

[B28] Lisik D. Zou D. (2025). Treatable traits-based pharmacologic treatment of sleep apnea. Sleep Med. Clinics 20, 103–114. doi: 10.1016/j.jsmc.2024.10.002 39894591

[B29] Liu R. Li C. Xu H. Wu K. Li X. Liu Y. . (2022). Fusion of whole night features and desaturation segments combined with feature extraction for event-level screening of sleep-disordered breathing. Nat. Sci. Sleep 14, 927–940. doi: 10.2147/NSS.S355369 PMC9123935 35607445

[B30] Liu T. Que L. Bai W. Yao H. (2026). Machine learning optimization of obstructive sleep apnea screening: development and validation of a gradient boosting prediction model with a clinical implementation framework. Front. Med. 13:1775766. doi: 10.3389/fmed.2026.1775766 PMC13106432 42040608

[B31] Liu Y. Li Y. Zhao M. Xiong N. Guan M. Li H.-C. . (2026). Dfcnet: a precise detection approach for obstructive sleep apnea-hypopnea events using airflow and respiratory effort signals. IEEE J. Biomed. Health Informat. 1–11. doi: 10.1109/JBHI.2026.3688540 42048212

[B32] Macey P. M. (2012). Is brain injury in obstructive sleep apnea reversible? Sleep 35, 9–10. doi: 10.5665/sleep.1572 22215912 PMC3242693

[B33] Midelet A. Prigent A. Schaeffer M.-C. Hy R. L. Pepin J.-L. Bailly S. (2023). Features of cheyne-stokes respiration automatically extracted from cpap airflow signal raw data: Identification of discriminating features to detect heart failure. Biomed. Signal Proc. Control 86:105227. doi: 10.1016/j.bspc.2023.105227

[B34] Mosteiro-Añón M. Casal-Guisande M. Fernández-Villar A. Torres-Durán M. (2025). AI-driven clinical decision support for early diagnosis and treatment planning in patients with suspected sleep apnea using clinical and demographic data before sleep studies. NPJ Primary Care Respir. Med. 35:51. doi: 10.1038/s41533-025-00455-5 41266418 PMC12635113

[B35] Nassi T. E. Ganglberger W. Sun H. Bucklin A. A. Biswal S. Putten M. J. A. M. V. . (2022). Automated scoring of respiratory events in sleep with a single effort belt and deep neural networks. IEEE Transact. Biomed. Eng. 69, 2094–2104. doi: 10.1109/TBME.2021.3136753 PMC9119908 34928786

[B36] Nikkonen S. Korkalainen H. Leino A. Myllymaa S. Duce B. Leppänen T. . (2021). Automatic respiratory event scoring in obstructive sleep apnea using a long short-term memory neural network. IEEE J. Biomed. Health Informat. 25, 2917–2927. doi: 10.1109/JBHI.2021.3064694 33687851

[B37] Olsen M. Zeitzer J. M. Nakase-Richardson R. Musgrave V. H. Sorensen H. B. D. Mignot E. . (2024). A deep transfer learning approach for sleep stage classification and sleep apnea detection using wrist-worn consumer sleep technologies. IEEE Transact. Biomed. Eng. 71, 2506–2517. doi: 10.1109/TBME.2024.3378480 38498753

[B38] Padovano D. Martinez-Rodrigo A. Pastor J. M. Rieta J. J. Alcaraz R. (2022). On the generalization of sleep apnea detection methods based on heart rate variability and machine learning. IEEE Access 10, 92710–92725. doi: 10.1109/ACCESS.2022.3201911

[B39] Pataka A. Pepin J. Bonsignore M. Schiza S. Saaresranta T. Bouloukaki I. . (2024). Sleep apnoea phenotypes in women: a cluster analysis from the esada cohort. Sleep Med. 124, 494–501. doi: 10.1016/j.sleep.2024.10.015 39437459

[B40] Peng D. Yue H. Tan W. Lei W. Chen G. Shi W. Zhang Y. (2024). A bimodal feature fusion convolutional neural network for detecting obstructive sleep apnea/hypopnea from nasal airflow and oximetry signals. Artificial Intel. Med. 150:102808. doi: 10.1016/j.artmed.2024.102808 38553148

[B41] Retamales G. Gavidia M. E. Bausch B. Montanari A. N. Husch A. Goncalves J. (2024). Towards automatic home-based sleep apnea estimation using deep learning. NPJ Dig. Med. 7:144. doi: 10.1038/s41746-024-01139-z 38824175 PMC11144223

[B42] Rohrscheib C. Moura A. A. Raphelson J. Orr J. E. Patel R. P. Malhotra A. (2026). Evaluation of an automated sleep apnea scoring algorithm via the wesper lab home sleep apnea test. Sleep Med. 141:108828. doi: 10.1016/j.sleep.2026.108828 41671846 PMC12945445

[B43] Sharma M. Kumbhani D. Tiwari J. Kumar T. S. Acharya U. R. (2022). Automated detection of obstructive sleep apnea in more than 8000 subjects using frequency optimized orthogonal wavelet filter bank with respiratory and oximetry signals. Comput. Biol. Med. 144:105364. doi: 10.1016/j.compbiomed.2022.105364 35299046

[B44] Shi Y. Zhang Y. Cao Z. Ma L. Yuan Y. Niu X. . (2023). Application and interpretation of machine learning models in predicting the risk of severe obstructive sleep apnea in adults. BMC Med. Informat. Decision Making 23:230. doi: 10.1186/s12911-023-02331-z 37858225 PMC10585776

[B45] Song Y. Ding L. Peng J. Song L. Zhang X. (2025). Screening for obstructive sleep apnea hypopnea using sleep breathing sounds based on the psg-audio dataset. Biomed. Signal Proc. Control 103:107472. doi: 10.1016/j.bspc.2024.107472

[B46] Su Y. Wang Z. Chang H. Zhu S. Zhou Y. Cao Z. . (2024). Craniofacial development characteristics in children with obstructive sleep apnea for establishment and external validation of the prediction model. Nat. Sci. Sleep 16, 2151–2170. doi: 10.2147/NSS.S492714 39723200 PMC11669283

[B47] TaghiBeyglou B. Geng J. McDaulid D. Gnaneswaran P. Yasokaran O. Chow A. . (2026). Self-supervised speech representations for sleep apnea severity prediction. IEEE transactions on biomedical engineering. 1–11. doi: 10.1109/TBME.2026.3656326 41557573

[B48] Talukder A. Li Y. Yeung D. Shi M. Umbach D. M. Fan Z. . (2024). Osapredictor: A tool for prediction of moderate to severe obstructive sleep apnea-hypopnea using readily available patient characteristics. Comput. Biol. Med. 178:108777. doi: 10.1016/j.compbiomed.2024.108777 38901189 PMC11265974

[B49] Tian S. Xia Y. Zhang Y. (2026). Hamce-net: Heterogeneity-aware multimodal cross-epoch network for sleep apnea detection. IEEE Access 14, 62075–62086. doi: 10.1109/ACCESS.2026.3686169

[B50] Tirachaimongkol V. Banhiran W. Chotinaiwattarakul W. Pimolsri C. Srikajon J. Kasemsuk N. . (2025). Belun sleep platform versus in-lab polysomnography for obstructive sleep apnea diagnosis. Sleep and Breathing 29:266. doi: 10.1007/s11325-025-03433-w PMC12331828 40773145

[B51] Wang B. Tang X. Ai H. Li Y. Xu W. Wang X. . (2022). Obstructive sleep apnea detection based on sleep sounds via deep learning. Nat. Sci. Sleep 14, 2033–2045. doi: 10.2147/NSS.S373367 36394068 PMC9653035

[B52] Wang Y. Liu Q. Min F. Wang H. (2026). Psg-mae: robust multitask sleep event monitoring using multichannel psg reconstruction and inter-channel contrastive learning. IEEE Transact. Neural Syst. Rehabil. Eng. 34, 274–286. doi: 10.1109/TNSRE.2025.3645353 41406297

[B53] Wang Z. Yang T. Zhu Y. Liu J. Cistulli P. A. Chen W. (2025). Multi-task learning for OSA detection and sleep staging via multi-scale modeling. IEEE J. Biomed. Health Informat. doi: 10.1109/JBHI.2025.3647317 41433180

[B54] Whiting P. F. Rutjes A. W. S. Westwood M. E. Mallett S. Deeks J. J. Reitsma J. B. . (2011). Quadas-2: a revised tool for the quality assessment of diagnostic accuracy studies. Ann. Int. Med. 155, 529–536. doi: 10.7326/0003-4819-155-8-201110180-00009 22007046

[B55] Wolff R. F. Moons K. G. M. Riley R. D. Whiting P. F. Westwood M. Collins G. S. . (2019). Probast: a tool to assess the risk of bias and applicability of prediction model studies. Ann. Int. Med. 170, 51–58. doi: 10.7326/M18-1376 30596875

[B56] Wu W. Yu Y. Wang Q. Liu D. Yuan X. (2021). Upper airway segmentation based on the attention mechanism of weak feature regions. IEEE Access 9, 95372–95381. doi: 10.1109/ACCESS.2021.3094032

[B57] Wu Z. Tan J. Wu X. Li Y. Huang S. Tan S. . (2026). A unified neural network framework for synchronized pressure control in home ventilators. Eng. Appl. Artificial Int. 174:114541. doi: 10.1016/j.engappai.2026.114541

[B58] Ye G. Yin H. Chen T. Chen H. Cui L. Zhang X. (2021). Fenet: A frequency extraction network for obstructive sleep apnea detection. IEEE J. Biomed. Health Informat. 25, 2848–2856. doi: 10.1109/JBHI.2021.3050113 33434137

[B59] Yoo H. Kim G. Kim T. Kim H. Y. Jung Y. G. Hong S. D. . (2026). Development of a multimodal obstructive sleep apnea diagnostic prediction model using two -dimensional facial images and clinical data. IEEE J. Biomed. Health Informat. doi: 10.1109/JBHI.2026.3678664 41911139

[B60] Zhang X. Li F. Fang Y. Zhong L. Ding K. Chen H. . (2026). Novel event-based peripheral oxygen saturation metrics provide complementary information for OSA classification. IEEE Transact. Biomed. Eng. 1–9. doi: 10.1109/TBME.2026.3665589 41701586

[B61] Zhang Y. Zhou L. Zhu S. Zhou Y. Wang Z. Ma L. . (2025). Deep learning for obstructive sleep apnea detection and severity assessment: a multimodal signals fusion multiscale transformer model. Nat. Sci. Sleep 17, 1–15. doi: 10.2147/NSS.S492806 39801628 PMC11720996

[B62] Zhao A. Hao B. Liu S. Qiu X. Ming X. Yang X. . (2024). A prediction nomogram of severe obstructive sleep apnea in patients with obesity based on the liver stiffness and abdominal visceral adipose tissue quantification. Nat. Sci. Sleep 16, 1515–1527. doi: 10.2147/NSS.S475534 39364433 PMC11448031

[B63] Zhao T. Li Z. Liu W. Fu X. Zhou Y. Zhou L. (2024). Unconstrained respiratory event detection using a flexible tactile sensor. IEEE Sensors J. 24, 30095–30103. doi: 10.1109/JSEN.2024.3442240

[B64] Zhou S.-J. Yang R. Wang L.-L. Qi M. Yuan X.-F. Wang T.-T. . (2023). Measuring sleep stages and screening for obstructive sleep apnea with a wearable multi-sensor system in comparison to polysomnography. Nat. Sci. Sleep 15, 353–362. doi: 10.2147/NSS.S406359 37193215 PMC10182819

