# Supplementary material for: Artificial intelligence for sleep-disordered breathing: a taxonomy of methods, data modalities, and clinical applications
Source: Front Sleep. 2026 Sep 18;5:1927453. doi: 10.3389/frsle.2026.1927453 (PMC13631472; doi:10.3389/frsle.2026.1927453)
Supplement: Supplementary file 1 [file Data_Sheet_1.pdf]

Supplementary Material

Table S1: Publisher-specific search strategy, filtering conditions, and screening records used in this review.

Publisher	Search query	Main filters	Relevant journals, domains considered
ScienceDirect / Elsevier	`("obstructive sleep apnea" OR "sleep apnea" OR "sleep-disordered breathing" OR OSA) AND ("machine learning" OR "deep learning" OR "artificial intelligence") AND (diagnosis OR screening)`	Article type: research articles; publication years 2021–2026; subject areas mainly Computer Science, Medicine and Dentistry, Engineering, Neuroscience. Records: 516 identified / 516 assessed.	Relevant publication journals included *Biomedical Signal Processing and Control*, *Sleep Medicine*, *Computers in Biology and Medicine*, *Journal of Medical Internet Research*, *Computer Methods and Programs in Biomedicine*, *Artificial Intelligence in Medicine*, *Respiratory Medicine*, *CHEST*, *Neurocomputing*, *Information Fusion*, and *Knowledge-Based Systems*.
SpringerLink	`("obstructive sleep apnea" OR "sleep apnea" OR "sleep disordered breathing") AND ("machine learning" OR "deep learning" OR "artificial intelligence") AND (diagnos* OR screen*)`	Content type: research article; open access; English; publication years 2021–2026. Records: 153 identified / 153 assessed.	Disciplines and subdisciplines included *Medicine & Public Health*, *Computer Science*, *Biomedicine*, *Engineering*, *Internal Medicine*, *Neurology*, *Pneumology/Respiratory System*, *Otorhinolaryngology*, *Artificial Intelligence*, *Health Informatics*, *Biomedical Engineering*, *Neurosciences*, *Machine Learning*, *Sleep Disorders*, *Predictive Medicine*, *Wearable Technology*, *Electroencephalography*, and *OSA Diagnosis and Management*.
IEEE Xplore	`("obstructive sleep apnea" OR "sleep apnea" OR "sleep disordered breathing") AND ("machine learning" OR "deep learning" OR "artificial intelligence") AND (diagnos* OR screen*)`	Search field: All Metadata; filters included journals, early access articles, and publication years 2021–2026. One duplicate PDF was removed before final screening. Records: 131 identified / 130 assessed.	IEEE Xplore engineering and biomedical journals were considered when studies addressed OSA/SDB diagnosis, screening, event detection, severity estimation, wearable sensing, contactless monitoring, or deployable AI.
Taylor & Francis Online	`[All: "obstructive sleep apnea"] AND ([All: "machine learning"] OR [All: "deep learning"] OR [All: "artificial intelligence"])`	Article type: article; open access; subject category Medicine, Nursing & Allied Health, Dentistry; publication date 01/01/2021–12/31/2026. Records: 93 identified / 93 assessed.	Highly relevant journals included *Nature and Science of Sleep* and *International Journal of Chronic Obstructive Pulmonary Disease*, *Annals of Medicine* and *Diabetes, Metabolic Syndrome and Obesity: Targets and Therapy*.
Frontiers Online	`"obstructive sleep apnea" AND ("machine learning" OR "deep learning" OR "artificial intelligence")`	Article type: Original Research; Health domain; publication years 2021–2026; not part of a Research Topic. Records: 151 identified / 151 assessed.	Journal-level filters focused on *Frontiers in Digital Health*, *Frontiers in Medicine*, *Frontiers in Neurology*, *Frontiers in Medical Technology*, and *Frontiers in Radiology*. Relevant sections included Health Informatics, Connected Health, Personalized Medicine, Pulmonary Medicine, Precision Medicine, Sleep Disorders.

Search-scope note: The predefined search covered the five publisher platforms listed above. PubMed/MEDLINE, Embase, Scopus, Web of Science, Wiley Online Library, the ACM Digital Library, arXiv, and medRxiv were not systematically searched. The open-access filters on SpringerLink and Taylor & Francis Online may have excluded subscription-only articles and introduced access-related selection bias; the resulting corpus is therefore a scoped evidence map rather than an exhaustive census of the literature.

Table S2: Distribution of core Elsevier, SpringerLink, Taylor & Francis, Frontiers, and IEEE Xplore studies across the proposed AI-for-SDB taxonomy. Counts are multi-label assignments because one paper may contribute to more than one subsection.

Group	Subsection	Elsevier	Springer	T&F	Frontiers	IEEE	Total
G1	PSG and HSAT signal analysis	31	5	4	1	21	62
G2	Wearable, smartphone, and contactless screening	15	10	12	1	17	55
G3	Audio, oximetry, ECG, PPG, and respiratory-signal diagnosis	81	17	14	3	60	175
G4	Imaging, craniofacial, and anatomical risk prediction	7	3	8	0	4	22
G5	Phenotyping, endotyping, and risk stratification	15	9	10	2	8	44
G6	Treatment management, CPAP adherence, and remote monitoring	4	1	0	0	1	6
G7	Explainable, trustworthy, and clinically deployable AI	59	22	25	4	65	175

Table S3: Deduplicated evidence map of the 218 core AI-for-SDB studies. Each study appears once. An **X** indicates assignment to a taxonomy domain: G1, PSG/HSAT signal analysis; G2, wearable, smartphone, and contactless screening; G3, audio, oximetry, ECG, PPG, and respiratory-signal diagnosis; G4, imaging, craniofacial, and anatomical risk prediction; G5, phenotyping, endotyping, and risk stratification; G6, treatment management, CPAP adherence, and remote monitoring; and G7, explainable, trustworthy, and clinically deployable AI. Domain assignments are non-mutually-exclusive.

Ref.	Publisher	Data modality	Clinical / technical task	AI method	G1: PSG/HSAT	G2: wearable/contactless	G3: physiological signals	G4: imaging/anatomy	G5: phenotyping/risk	G6: treatment/monitoring	G7: trustworthy/deployable	Main finding / review relevance
(Akter et al., 2026b)	Elsevier	Single-lead ECG scalograms	Sleep-apnea detection	Deep residual ECG apnea model; Grad-CAM			X				X	Adds interpretability or transparency to sleep-apnea prediction, improving clinical inspectability of model outputs.
(Akter et al., 2026a)	Elsevier	ECG; multi-view ECG representations	Sleep-apnea detection	Multi-view interactive CNN; explainable AI			X				X	Adds interpretability or transparency to sleep-apnea prediction, improving clinical inspectability of model outputs.
(Almutairi and Hassan, 2021)	Elsevier	Single-lead ECG	OSA classification	CNN-LSTM			X					Shows that ECG/HRV-derived patterns can detect sleep-apnea events or classify OSA severity using reduced-channel data.
(Balci et al., 2022)	Elsevier	PSG pressure flow; ECG; snore; SpO_2; pulse; thorax signals	Classify normal, hypopnea, obstructive apnea, mixed apnea, central apnea	Time/time-frequency features; ML classifiers	X		X					Supports oximetry-based detection or severity estimation using desaturation and overnight oxygen-pattern information.
(Bernardini et al., 2021)	Elsevier	Physiological monitoring signals	OSA event identification	Deep learning	X		X				X	Supports OSA event identification using physiological monitoring signals with deep learning.
(Bhalerao and Pachori, 2022)	Elsevier	EEG from MIT-BIH polysomnographic database	Sleep-apnea classification	Sparse-spectrum swarm decomposition; classifier	X							Shows that PSG-derived neurophysiological signals can contribute to event detection, staging, or severity classification.
(Bhongade et al., 2025)	Elsevier	Single-lead ECG; multimodal ECG-derived features	OSA identification	Time-domain, time-frequency, entropy features; ML classifiers			X					Shows that ECG/HRV-derived patterns can detect sleep-apnea events or classify OSA severity using reduced-channel data.
(Bokov et al., 2023)	Elsevier	Clinical exam; acoustic rhinometry; pharyngometry; questionnaire	Moderate-to-severe pediatric OSAS screening	Machine-learning screening test				X	X			Provides a screening model based on readily available clinical or physiological variables for OSA risk stratification.
(Bricout et al., 2021)	Elsevier	Dual chest accelerometry; airflow estimation	AHI estimation and sleep-apnea screening	Machine learning; accelerometry-derived respiratory index		X	X					Supports respiratory-signal-based event detection, AHI estimation, or advance prediction of sleep-apnea episodes.
(Cai et al., 2025)	Elsevier	Single-lead ECG	OSA detection	Temporal proximity contrastive learning			X				X	Addresses limited-label or cross-domain robustness, supporting more generalizable sleep-apnea detection.
(Chen et al., 2022)	Elsevier	Single-lead ECG	Sleep-apnea detection	Lightweight multi-scale CNN; channel-wise attention			X				X	Shows that ECG/HRV-derived patterns can detect sleep-apnea events or classify OSA severity using reduced-channel data.

Continued on next page

Ref.	Publisher	Data modality	Clinical / technical task	AI method	G1: PSG/HSAT	G2: wearable/contactless	G3: physiological signals	G4: imaging/anatomy	G5: phenotyping/risk	G6: treatment/monitoring	G7: trustworthy/deployable	Main finding / review relevance
(Chen et al., 2023b)	Elsevier	Contactless breathing vibration signals	Respiratory-event detection and AHI estimation	Modified U-Net		X	X				X	Supports respiratory-event detection and AHI estimation using contactless breathing vibration signals with modified U-Net.
(Chen et al., 2023a)	Elsevier	Overnight unsegmented SpO_2	OSA severity classification	Deep neural network			X					Supports oximetry-based detection or severity estimation using desaturation and overnight oxygen-pattern information.
(Cheng et al., 2022)	Elsevier	Snoring signal/audio	OSAHS detection and severity classification	LSTM neural network		X	X					Demonstrates audio-based or speech-based screening for OSA/SDB, supporting low-burden contactless assessment.
(Cheng et al., 2023)	Elsevier	EEG	OSA event detection under fragment-label ambiguity	Multiple-instance learning	X		X				X	Shows that PSG-derived neurophysiological signals can contribute to event detection, staging, or severity classification.
(Choudhury et al., 2025)	Elsevier	Single-lead ECG scalograms	Sleep-apnea detection	Optimized transfer learning; LIME			X				X	Adds interpretability or transparency to sleep-apnea prediction, improving clinical inspectability of model outputs.
(Chu et al., 2024)	Elsevier	SpO_2; nasal airflow	OSA event detection with visualization/interpretability	Feature sieve; decision-boundary formulation	X		X				X	Adds interpretability or transparency to sleep-apnea prediction, improving clinical inspectability of model outputs.
(Chu et al., 2025)	Elsevier	PSG nasal pressure; SpO_2	Apnea-hypopnea event-boundary detection, AHI estimation, SAHS severity	Deep event-detection model	X		X				X	Supports oximetry-based detection or severity estimation using desaturation and overnight oxygen-pattern information.
(Dong et al., 2025)	Elsevier	Single-channel ECG	Normal/hypopnea/apnea event classification	CNN			X					Shows that ECG/HRV-derived patterns can detect sleep-apnea events or classify OSA severity using reduced-channel data.
(Fang et al., 2025)	Elsevier	Simulated snoring sounds	OSA diagnosis at AHI thresholds	ML/DL classifiers			X					Demonstrates audio-based or speech-based screening for OSA/SDB, supporting low-burden contactless assessment.
(Faust et al., 2021)	Elsevier	RR intervals from ECG	Sleep-apnea detection	LSTM			X					Shows that ECG/HRV-derived patterns can detect sleep-apnea events or classify OSA severity using reduced-channel data.
(Ganguly et al., 2025)	Elsevier	Single-lead ECG	Sleep apnea diagnosis	Residual deep learning; relevance maps/XAI			X				X	Adds interpretability or transparency to sleep-apnea prediction, improving clinical inspectability of model outputs.
(Jimenez-Garcia et al., 2022)	Elsevier	Airflow; SpO_2; pediatric PSG	Pediatric OSA detection, AHI/severity estimation	2D CNN	X		X					Shows that pediatric OSA can be detected or stratified from overnight physiological signals, supporting child-focused screening.
(Jimenez-Garcia et al., 2024)	Elsevier	Overnight airflow; SpO_2; pediatric PSG	Pediatric OSA identification, AHI/severity classification	CNN/RNN; Grad-CAM	X		X				X	Shows that pediatric OSA can be detected or stratified from overnight physiological signals, supporting child-focused screening.

Continued on next page

Ref.	Publisher	Data modality	Clinical / technical task	AI method	G1: PSG/HSAT	G2: wearable/contactless	G3: physiological signals	G4: imaging/anatomy	G5: phenotyping/risk	G6: treatment/monitoring	G7: trustworthy/deployable	Main finding / review relevance
(Gayen et al., 2025)	Elsevier	Single-lead ECG	OSA classification with limited annotations	Semi-supervised learning; adaptive pseudo-labeling			X				X	Addresses limited-label or cross-domain robustness, supporting more generalizable sleep-apnea detection.
(Gayen et al., 2026)	Elsevier	Single-lead ECG	Sleep-apnea event classification	Semi-supervised prototype-aware contrastive learning			X				X	Addresses limited-label or cross-domain robustness, supporting more generalizable sleep-apnea detection.
(Bahr-Hamm et al., 2023)	Elsevier	Snoring; respiratory effort; ECG-VLF	OSA detection and severity classification	Entropy features; SVM	X		X		X			Demonstrates audio-based or speech-based screening for OSA/SDB, supporting low-burden contactless assessment.
(Hanif et al., 2023)	Elsevier	Drug-induced sleep endoscopy video	Airway-obstruction scoring for OSA treatment planning	Deep learning				X		X	X	Supports automated airway-obstruction scoring from endoscopy video for OSA treatment planning.
(He et al., 2023)	Elsevier	Craniofacial photographs	OSA detection and facial-region localization	Explainable deep learning				X			X	Links anatomical imaging or craniofacial morphology with OSA risk prediction and airway assessment.
(Holfinger et al., 2022)	Elsevier	Readily available clinical variables	OSA prediction / screening compared with STOP-BANG	ANN; random forest; kernel SVM					X		X	Provides a screening model based on readily available clinical or physiological variables for OSA risk stratification.
(Hou et al., 2025b)	Elsevier	Wearable PPG / multi-source representations	OSAS screening	Transformer-based representation fusion		X	X				X	Shows that PPG-derived features can support wearable sleep-apnea screening or severity-related assessment.
(Hou et al., 2025a)	Elsevier	Single-lead ECG	OSA detection	Dilated convolution; LSTM; multi-head attention; voting			X				X	Shows that ECG/HRV-derived patterns can detect sleep-apnea events or classify OSA severity using reduced-channel data.
(Hu et al., 2025)	Elsevier	Multimodal PSG/ECG sleep datasets	Sleep-apnea detection and sleep staging	Interpretable multimodal fusion; information bottleneck	X		X				X	Adds interpretability or transparency to sleep-apnea prediction, improving clinical inspectability of model outputs.
(Ji et al., 2024)	Elsevier	PSG respiratory signals	Apnea/hypopnea localization with onset and duration	Multi-scale object detection; dual-modal feature learning	X		X				X	Supports respiratory-signal-based event detection, AHI estimation, or advance prediction of sleep-apnea episodes.
(Jiang et al., 2021)	Elsevier	Snoring sounds	OSAHS evaluation / simple snorer vs OSAHS classification	Acoustic features; feature selection; ML		X	X					Demonstrates audio-based or speech-based screening for OSA/SDB, supporting low-burden contactless assessment.
(Jiang et al., 2025a)	Elsevier	SpO_2 fluctuation patterns; pediatric PSG	Apnea-hypopnea event detection and AHI agreement	Enhanced genetic algorithm	X		X				X	Shows that pediatric OSA can be detected or stratified from overnight physiological signals, supporting child-focused screening.
(Jiang et al., 2025b)	Elsevier	ECG; SpO_2	Sleep-apnea syndrome detection	CNN/RNN multimodal fusion; MMTM-SE			X				X	Supports oximetry-based detection or severity estimation using desaturation and overnight oxygen-pattern information.
(Juang et al., 2021)	Elsevier	Easy-to-obtain physiological features	Moderate-to-severe OSAHS screening	Explainable fuzzy neural network					X		X	Adds interpretability or transparency to sleep-apnea prediction, improving clinical inspectability of model outputs.
(Kim et al., 2024)	Elsevier	19-channel sleep EEG with PSG labels	AHI-defined OSA classification	Spectral/network/microstate features; ML	X				X			Shows that PSG-derived neurophysiological signals can contribute to event detection, staging, or severity classification.

Continued on next page

Ref.	Publisher	Data modality	Clinical / technical task	AI method	G1: PSG/HSAT	G2: wearable/contactless	G3: physiological signals	G4: imaging/anatomy	G5: phenotyping/risk	G6: treatment/monitoring	G7: trustworthy/deployable	Main finding / review relevance
(Kim et al., 2025b)	Elsevier	Wearable-compatible HRV and SpO_2 signals	Normal/apnea/hypopnea event detection and AHI computation	2D CNNs		X	X				X	Supports oximetry-based detection or severity estimation using desaturation and overnight oxygen-pattern information.
(Leino et al., 2021)	Elsevier	Nocturnal SpO_2	Sleep-apnea severity screening in stroke/TIA	CNN / neural network			X				X	Supports oximetry-based detection or severity estimation using desaturation and overnight oxygen-pattern information.
(Li et al., 2023b)	Elsevier	Snoring sounds; non-contact audio	OSAHS screening	Visibility-graph encoding; CNN		X	X					Demonstrates audio-based or speech-based screening for OSA/SDB, supporting low-burden contactless assessment.
(Li et al., 2023a)	Elsevier	EEG; oral-nasal airflow; multicenter PSG	Sleep apnea/respiratory episode detection; AHI agreement	Deep learning	X		X				X	Supports respiratory-signal-based event detection, AHI estimation, or advance prediction of sleep-apnea episodes.
(Li et al., 2025b)	Elsevier	Resting-state fMRI connectivity	OSA identification and symptom prediction	Static/dynamic FC; ML				X	X		X	Identifies neuroimaging or EEG-network alterations associated with OSA/SAHS and symptom or severity profiles.
(Li et al., 2026)	Elsevier	Snoring sounds	Sleep-apnea detection with cross-subject generalization	CNN; Transformer encoder; adversarial domain adaptation		X	X				X	Demonstrates audio-based or speech-based screening for OSA/SDB, supporting low-burden contactless assessment.
(Lin et al., 2022a)	Elsevier	Snoring signals; embedded hardware	Contactless sleep-apnea detection	Hybrid DNN; pruning/quantization		X	X				X	Demonstrates a deployable or resource-efficient route for real-time sleep-apnea or respiratory-event monitoring.
(Liu et al., 2023)	Elsevier	Single-channel ECG	OSA detection	CNN-transformer			X					Shows that ECG/HRV-derived patterns can detect sleep-apnea events or classify OSA severity using reduced-channel data.
(Mashrur et al., 2021)	Elsevier	Single-lead ECG scalograms	OSA detection	Scalogram-based CNN; CWT/EMD			X				X	Shows that ECG/HRV-derived patterns can detect sleep-apnea events or classify OSA severity using reduced-channel data.
(Midelet et al., 2023)	Elsevier	CPAP airflow raw data	CSR feature extraction and heart-failure-related monitoring	Change-point detection; feature extraction; logistic regression			X			X	X	Shows how treatment-device or CPAP-derived signals can support adaptive control or residual respiratory monitoring.
(Monna et al., 2022)	Elsevier	3D craniofacial scans	OSA screening/prediction	Geometric morphometrics; supervised ML				X	X			Links anatomical imaging or craniofacial morphology with OSA risk prediction and airway assessment.
(Moradhasel et al., 2023)	Elsevier	Chin EMG spectrograms	OSA diagnosis	Pretrained deep-learning models	X		X				X	Shows that PSG-derived neurophysiological signals can contribute to event detection, staging, or severity classification.
(Nassehi et al., 2024)	Elsevier	Awake resting-state EEG	OSA severity classification	Machine learning	X				X			Shows that PSG-derived neurophysiological signals can contribute to event detection, staging, or severity classification.

Continued on next page

Ref.	Publisher	Data modality	Clinical / technical task	AI method	G1: PSG/HSAT	G2: wearable/contactless	G3: physiological signals	G4: imaging/anatomy	G5: phenotyping/risk	G6: treatment/monitoring	G7: trustworthy/deployable	Main finding / review relevance
(Pan et al., 2021)	Elsevier	PSG EEG / cortical connectivity	SAHS diagnosis and severity classification	Symbolic transfer entropy; connectivity classification	X				X		X	Identifies neuroimaging or EEG-network alterations associated with OSA/SAHS and symptom or severity profiles.
(Parbat and Chakraborty, 2024)	Elsevier	Single-lead ECG; instantaneous HR; ECG-derived respiration	Sleep-apnea event prediction/classification	Multiscale entropy; ML			X					Shows that ECG/HRV-derived patterns can detect sleep-apnea events or classify OSA severity using reduced-channel data.
(Pataka et al., 2024)	Elsevier	ESADA cohort; clinical/PSG variables	Female-specific OSA phenotype discovery	Latent class / cluster analysis					X			Uses data-driven grouping to reveal clinically meaningful OSA/SDB phenotypes and outcome differences.
(Peng et al., 2024)	Elsevier	Nasal airflow; SpO_2	OSA/hypopnea detection and severity assessment	Bimodal feature-fusion CNN	X		X					Supports oximetry-based detection or severity estimation using desaturation and overnight oxygen-pattern information.
(Pham and Moucek, 2025)	Elsevier	Single-lead ECG; RR/R-peak signals	Sleep-apnea detection	CNN-Transformer-LSTM			X					Shows that ECG/HRV-derived patterns can detect sleep-apnea events or classify OSA severity using reduced-channel data.
(Qi et al., 2026)	Elsevier	ECG time-domain and time-frequency features	Sleep staging and sleep-apnea detection	Cross-modal deep-learning fusion	X		X				X	Shows that ECG/HRV-derived patterns can detect sleep-apnea events or classify OSA severity using reduced-channel data.
(Qin and Liu, 2022)	Elsevier	Single-lead ECG / RR intervals	Sleep-apnea detection	1D-CNN representation learning; BiGRU; ADASYN			X					Shows that ECG/HRV-derived patterns can detect sleep-apnea events or classify OSA severity using reduced-channel data.
(Rajesh et al., 2021)	Elsevier	Single-lead ECG	OSA detection	DWT statistical features; PSO feature selection; ML			X					Shows that ECG/HRV-derived patterns can detect sleep-apnea events or classify OSA severity using reduced-channel data.
(Rehman et al., 2025)	Elsevier	Chin EMG from PSG	OSA event screening/differentiation	Motor-unit decomposition; ML classifiers	X		X					Shows that PSG-derived neurophysiological signals can contribute to event detection, staging, or severity classification.
(Rehman and Saleh, 2025)	Elsevier	Chin EMG; low-power hardware	Central apnea and hypopnea screening	FNN/SVM; ultra-low-power SoC	X		X				X	Demonstrates a deployable or resource-efficient route for real-time sleep-apnea or respiratory-event monitoring.
(Rohrscheib et al., 2026)	Elsevier	HSAT signals	Automated scoring of AHI, CAI, obstructive apnea, central apnea, hypopnea	AI-based automated scoring algorithm	X		X				X	Validates automated HSAT scoring for respiratory events and AHI-related indices against clinical sleep-testing workflows.
(Ryu et al., 2021)	Elsevier	Medical airway images; CFD airflow features	OSA diagnosis and airflow-characteristics prediction	DL segmentation; Gaussian process regression; SVM				X			X	Links anatomical imaging or craniofacial morphology with OSA risk prediction and airway assessment.
(Saha et al., 2025)	Elsevier	Tracheal breathing sounds	Distinguish obstructive vs central respiratory events	Transfer learning; pretrained CNNs			X				X	Demonstrates audio-based or speech-based screening for OSA/SDB, supporting low-burden contactless assessment.

Continued on next page

Ref.	Publisher	Data modality	Clinical / technical task	AI method	G1: PSG/HSAT	G2: wearable/contactless	G3: physiological signals	G4: imaging/anatomy	G5: phenotyping/risk	G6: treatment/monitoring	G7: trustworthy/deployable	Main finding / review relevance
(Chao et al., 2025)	Elsevier	Neck-wearable piezoelectric sensor	Severe sleep-apnea vs habitual snoring classification	Hybrid CNN-GRU		X	X				X	Demonstrates audio-based or speech-based screening for OSA/SDB, supporting low-burden contactless assessment.
(Sharma et al., 2022)	Elsevier	SpO_2; airflow; thoracic/abdominal respiratory signals; SHHS	Automated OSA detection	Wavelet filter bank; ML classifiers	X		X					Supports oximetry-based detection or severity estimation using desaturation and overnight oxygen-pattern information.
(Shen et al., 2025)	Elsevier	Single-lead ECG	Sleep apnea syndrome detection with limited labels	Self-supervised contrastive restoration; GAN/CNN transfer			X				X	Addresses limited-label or cross-domain robustness, supporting more generalizable sleep-apnea detection.
(Song et al., 2025)	Elsevier	Whole-night breathing sounds; PSG-Audio dataset	OSAHS screening and AHI estimation	ConvNeXt + LSTM		X	X				X	Demonstrates audio-based or speech-based screening for OSA/SDB, supporting low-burden contactless assessment.
(Srivastava et al., 2023)	Elsevier	Digitized ECG	OSA detection	1D CNN-LSTM			X				X	Shows that ECG/HRV-derived patterns can detect sleep-apnea events or classify OSA severity using reduced-channel data.
(Taghizadegan et al., 2021)	Elsevier	PSG EEG; ECG; respiration signals	OSA event prediction before occurrence	Recurrence plots; CNN	X		X					Shows that ECG/HRV-derived patterns can detect sleep-apnea events or classify OSA severity using reduced-channel data.
(Talukder et al., 2024)	Elsevier	Readily available patient characteristics	Moderate-to-severe OSAHS prediction/screening	XGBoost					X		X	Provides a screening model based on readily available clinical or physiological variables for OSA risk stratification.
(Tang and Liu, 2021)	Elsevier	ECG/HRV	OSA screening and severity-related analysis	Temporal-dependency complexity; ML classifiers			X		X			Shows that ECG/HRV-derived patterns can detect sleep-apnea events or classify OSA severity using reduced-channel data.
(Tani et al., 2026)	Elsevier	Microphone respiratory audio; jaw movement; HSAT	Respiratory-event detection and AHI estimation	SoundSleepNet; ViT; DNDF	X	X	X				X	Validates automated HSAT scoring for respiratory events and AHI-related indices against clinical sleep-testing workflows.
(Gutierrez-Tobal et al., 2026)	Elsevier	Pediatric clinical/PSG/treatment data	OSA phenotyping and treatment-response/recovery prediction	Subject-based association network; explainable AI					X	X	X	Uses data-driven grouping to reveal clinically meaningful OSA/SDB phenotypes and outcome differences.
(Tyagi and Agrawal, 2023)	Elsevier	Single-lead ECG	Sleep-apnea detection	Enhanced deep belief network			X				X	Shows that ECG/HRV-derived patterns can detect sleep-apnea events or classify OSA severity using reduced-channel data.
(Tyagi and Agrawal, 2024)	Elsevier	Single-lead ECG	Sleep-apnea detection	Spiking neural network			X				X	Shows that ECG/HRV-derived patterns can detect sleep-apnea events or classify OSA severity using reduced-channel data.
(Ullah et al., 2023)	Elsevier	Single-lead ECG	OSA diagnosis	Dual-convolutional dual-attention network; SVM fusion			X				X	Shows that ECG/HRV-derived patterns can detect sleep-apnea events or classify OSA severity using reduced-channel data.

Continued on next page

Ref.	Publisher	Data modality	Clinical / technical task	AI method	G1: PSG/HSAT	G2: wearable/contactless	G3: physiological signals	G4: imaging/anatomy	G5: phenotyping/risk	G6: treatment/monitoring	G7: trustworthy/deployable	Main finding / review relevance
(Ullah et al., 2025)	Elsevier	ECG	OSA detection under limited data and cross-dataset testing	CSAC-Net; self-attention			X				X	Addresses limited-label and cross-domain robustness, supporting more generalizable sleep-apnea detection.
(Garcia-Vicente et al., 2023)	Elsevier	Raw ECG from pediatric PSG/CHAT	Pediatric OSA diagnosis and AHI/severity estimation	CNN	X		X					Shows that pediatric OSA can be detected or stratified from overnight physiological signals, supporting child-focused screening.
(Wang et al., 2026a)	Elsevier	Sleep respiratory sounds	Apnea/hypopnea detection, AHI estimation, severity classification	Representation learning; cross-attention fusion		X	X					Demonstrates audio-based or speech-based screening for OSA/SDB, supporting low-burden contactless assessment.
(Wei et al., 2023)	Elsevier	PPG	Sleep-apnea detection	Multi-scale 1D-CNN / MS-Net		X	X				X	Shows that PPG-derived features can support wearable sleep-apnea screening or severity-related assessment.
(Wu et al., 2021d)	Elsevier	Nasal airflow	Sleep apnea-hypopnea event classification	Enhanced frequency extraction deep network	X		X					Supports respiratory-signal-based event detection, AHI estimation, or advance prediction of sleep-apnea episodes.
(Wu et al., 2021b)	Elsevier	ECG-derived heart-rate fluctuations	OSA / severe OSA detection and severity-related analysis	Information-based similarity; ML			X		X			Shows that ECG/HRV-derived patterns can detect sleep-apnea events or classify OSA severity using reduced-channel data.
(Wu et al., 2021a)	Elsevier	Wearable bracelet PPG / heart-rate fluctuations	Sleep-apnea screening and AHI correlation	Information-based similarity		X	X					Shows that PPG-derived features can support wearable sleep-apnea screening or severity-related assessment.
(Wu et al., 2026)	Elsevier	Home ventilator/APAP pressure-control data	Neural-network-enhanced APAP pressure control and patient–ventilator synchronization	Unified neural network framework						X	X	Shows how treatment-device or CPAP-derived signals can support adaptive control or residual respiratory monitoring.
(Xie et al., 2023)	Elsevier	Snoring audio	AHI estimation and OSA severity classification	Audio features; ML			X					Demonstrates audio-based or speech-based screening for OSA/SDB, supporting low-burden contactless assessment.
(Yang et al., 2022)	Elsevier	Single-lead ECG; HRV; ECG-derived respiration	OSA detection	1D squeeze-and-excitation residual group network			X				X	Shows that ECG/HRV-derived patterns can detect sleep-apnea events or classify OSA severity using reduced-channel data.
(Yang et al., 2026)	Elsevier	MRI brain-network features	OSAHS characterization and diagnostic utility	Co-classification network analysis; random forest				X	X		X	Supports osahs characterization and diagnostic utility using mri brain-network features with co-classification network analysis; random forest.
(Ye et al., 2026)	Elsevier	Respiratory signals	Sleep-apnea detection/evaluation	Spectrally enhanced hierarchical patch network			X				X	Supports respiratory-signal-based event detection, AHI estimation, or advance prediction of sleep-apnea episodes.
(Ylmaz et al., 2023)	Elsevier	Awake speech recordings	OSA screening	Nonlinear speech features; KNN/SVM			X					Demonstrates audio-based or speech-based screening for OSA/SDB, supporting low-burden contactless assessment.
(Yook et al., 2024)	Elsevier	PSG nasal flow; SpO_2; ECG; demographics	Apnea/hypopnea event detection and OSA severity classification	Xception deep network	X		X					Supports oximetry-based detection or severity estimation using desaturation and overnight oxygen-pattern information.

Continued on next page

Ref.	Publisher	Data modality	Clinical / technical task	AI method	G1: PSG/HSAT	G2: wearable/contactless	G3: physiological signals	G4: imaging/anatomy	G5: phenotyping/risk	G6: treatment/monitoring	G7: trustworthy/deployable	Main finding / review relevance
(Yu et al., 2022)	Elsevier	EEG; oronasal flow; abdominal displacement	SAHS event/subtype classification	LSTM-CNN	X		X					Supports respiratory-signal-based event detection, AHI estimation, or advance prediction of sleep-apnea episodes.
(Zarei et al., 2022)	Elsevier	Single-lead ECG	Sleep-apnea detection and AHI-based subject classification	CNN-LSTM / deep neural networks			X					Shows that ECG/HRV-derived patterns can detect sleep-apnea events or classify OSA severity using reduced-channel data.
(Zhang et al., 2025b)	Elsevier	EEG; EMG; sleep-stage labels	Apnea/hypopnea event detection	Random forest; explainability-oriented analysis	X		X				X	Shows that PSG-derived neurophysiological signals can contribute to event detection, staging, or severity classification.
(Zhang et al., 2026a)	Elsevier	Single-lead ECG	Sleep-apnea detection	Lightweight interpretable Cos-ECGNet			X				X	Adds interpretability or transparency to sleep-apnea prediction, improving clinical inspectability of model outputs.
(Zhang et al., 2026c)	Elsevier	Single-channel airflow	Real-time sleep-apnea event advance prediction	Deterministic learning; dynamical features; neural-network fusion	X		X				X	Demonstrates a deployable or resource-efficient route for real-time sleep-apnea or respiratory-event monitoring.
(Zhao et al., 2025)	Elsevier	Single-lead ECG RR intervals and R-peak amplitudes	Sleep-apnea detection with domain generalization	SE multi-scale ResNet			X				X	Addresses limited-label or cross-domain robustness, supporting more generalizable sleep-apnea detection.
(Bark et al., 2023)	Springer	ECG-derived respiratory signals; SpO_2	Confidence-aware sleep-apnea detection	Autoencoder features; CNN–LSTM; selective prediction		X	X				X	Introduces confidence-based rejection to improve reliability of reduced-channel sleep-apnea detection.
(Chen et al., 2025)	Springer	Pharyngeal MRI radiomics; clinical data	Severe vs. nonsevere OSA prediction	Radiomics with LR, SVM, RF, and GBM				X	X		X	Combines pharyngeal MRI radiomics with clinical variables to improve severe-OSA prediction.
(Chi et al., 2026)	Springer	Large clinical OSA dataset	OSA prediction and comparative benchmarking	Comparative ML/DL models					X		X	Provides a comparative ML/DL benchmark for OSA prediction and complements the review with model evaluation.
(Trang et al., 2025)	Springer	EEG and ECG from sleep testing	OSA/CSA/hypopnea event identification	Wavelet features; ensemble ML; deep learning	X		X				X	Uses EEG and ECG signals to identify sleep-related breathing events, including OSA, CSA, and hypopnea.
(Dietz-Terjung et al., 2021)	Springer	Actigraphy; respiratory inductance plethysmography	Sleep-stage estimation, AHI estimation, and SDB severity assessment	Nox BodySleep algorithm / recurrent neural network	X	X	X				X	Validates an out-of-center algorithm for estimating sleep stages and SDB severity from actigraphy and respiratory effort.
(Frija et al., 2025)	Springer	Smartphone microphone, accelerometer, and gyroscope	AHI estimation and sleep-apnea severity prediction	Sequential deep-learning respiratory-event scoring		X	X				X	Evaluates a smartphone-based system for estimating AHI and predicting sleep-apnea severity.
(Ge et al., 2024)	Springer	Clinical and laboratory variables	Adult OSAHS risk prediction	LR, SVM, boosting, RF, MLP					X		X	Compares common ML models for adult OSAHS risk prediction from routinely available clinical data.
(Hayano et al., 2025)	Springer	Apple Watch IMU; seismocardiographic and respiratory surrogates	Apnea/hypopnea epoch detection and AHI-related screening	Feature engineering; RF, GBM, kNN, LR, MLP		X	X				X	Demonstrates feasibility of Apple Watch IMU signals for respiratory-event detection and AHI-related screening.
(Ivanovska et al., 2021)	Springer	Sagittal spine MRI	Segmentation of OSA-relevant upper-airway organs	Deep cascaded segmentation / U-Net-like models				X			X	Automates segmentation of pharynx, tongue, and soft-palate structures relevant to anatomical OSA risk analysis.

Continued on next page

Ref.	Publisher	Data modality	Clinical / technical task	AI method	G1: PSG/HSAT	G2: wearable/contactless	G3: physiological signals	G4: imaging/anatomy	G5: phenotyping/risk	G6: treatment/monitoring	G7: trustworthy/deployable	Main finding / review relevance
(Iwasaki et al., 2021)	Springer	ECG R–R intervals; HRV	Sleep-apnea / SAS screening	LSTM		X	X					Shows that HRV and R–R interval dynamics can support accessible ECG-based sleep-apnea screening.
(Javeed et al., 2023)	Springer	Structured electronic health data	Sleep-apnea risk prediction	XGBoost feature selection; BiLSTM classification					X		X	Shows that electronic health data can support sleep-apnea prediction through hybrid feature-selection and sequence modeling.
(Tirachaimongkol et al., 2025)	Springer	SpO_2; heart rate; accelerometry from Belun Sleep Platform	AI-driven HSAT/device validation for OSA diagnosis	AI-driven home sleep testing algorithm	X	X	X				X	Compares an AI-driven home sleep testing platform with in-lab PSG for OSA diagnosis.
(Liu et al., 2024a)	Springer	Clinical single-lead ECG	Apnea-segment detection and patient-level OSA screening	EfficientNet; XGBoost			X				X	Shows strong ECG-based segment detection and patient-level screening using a clinical single-lead ECG dataset.
(Ma et al., 2026)	Springer	Wearable inertial-sensor gait biomarkers	OSA screening in Parkinson's disease	XGBoost		X			X		X	Shows that wearable gait biomarkers can support OSA screening in a comorbidity-specific population.
(Mandeville et al., 2024)	Springer	Transmembranous EMG spectrograms	Sleep-apnea diagnosis	Transformer-based deep learning; transfer learning			X				X	Introduces transmembranous EMG as an alternative physiological signal for DL-based sleep-apnea diagnosis.
(Yamane et al., 2025)	Springer	Single-lead ECG	Sleep-apnea / SAS detection with temporal-context windows	Machine learning with optimized time windows			X				X	Shows that selecting appropriate ECG time windows improves clinical-level SAS screening from single-lead ECG.
(Mosteiro-Añón et al., 2025)	Springer	Clinical and demographic pre-sleep-study data	Pre-study patient clustering, diagnostic triage, and treatment planning support	k-prototypes clustering; Random Forest ICDSS					X	X	X	Supports early clinical decision support for suspected sleep apnea before formal sleep testing.
(Niemi et al., 2026)	Springer	HRV from Sleep Heart Health Study	Sleep-apnea detection and severity-related physiological analysis	Nonlinear HRV / scale-dependent DFA features; ML analysis			X		X			Supports scalable sleep-apnea detection and severity profiling using nonlinear HRV measures.
(Retamales et al., 2024)	Springer	Abdominal movement; thoracic movement; SpO_2	Home-based AHI estimation and apnea/hypopnea/wake detection	DRIVEN deep-learning framework	X	X	X				X	Supports automatic home-based sleep-apnea estimation using combinations of respiratory movement and oximetry signals.
(Shi et al., 2023)	Springer	Clinical characteristics; questionnaires	Severe OSA risk prediction	LR, GBM, XGBoost, AdaBoost, bagging, MLP; SHAP					X		X	Combines clinical variables and interpretable ML to build a severe-OSA risk calculator.
(Strumpf et al., 2026)	Springer	ECG R–R intervals; SpO_2 variability	SDB/OSA classification from routinely collected waveforms	Spectral features; SVM			X				X	Shows that spectral ECG and oximetry features can classify SDB using routinely collected physiological waveforms.
(Thuptimdang et al., 2026)	Springer	PPG-derived PWA, PPI, and SpO_2	Home OSA segment detection and AHI-related screening	SVM classifiers		X	X				X	Shows that PPG-derived pulse amplitude and interval features add value to SpO_2 for home OSA screening.
(Tsuiki et al., 2021)	Springer	Lateral cephalometric radiographs	Image-based severe OSA detection	Deep CNN / image-based ML				X				Shows that cephalometric imaging can contribute to anatomical screening for severe OSA.
(Xie et al., 2024)	Springer	RR intervals; respiratory effort	Sleep–wake classification, SDB event detection, AHI estimation, and severity classification	Multi-task CNN/RNN model	X		X				X	Demonstrates multi-task reduced-channel modeling for event detection and severity assessment in SDB.

Continued on next page

Ref.	Publisher	Data modality	Clinical / technical task	AI method	G1: PSG/HSAT	G2: wearable/contactless	G3: physiological signals	G4: imaging/anatomy	G5: phenotyping/risk	G6: treatment/monitoring	G7: trustworthy/deployable	Main finding / review relevance
(Xu et al., 2023)	Springer	Wearable intelligent sleep monitor; oximetry-derived information	Wearable OSA diagnostic validation against PSG	AI-assisted wearable sleep-monitor algorithm		X	X				X	Evaluates a wearable intelligent sleep monitor against PSG for OSA diagnosis, supporting scalable pre-screening.
(Zhang et al., 2024)	Springer	HRV features; clinical and demographic variables	Moderate-to-severe OSA screening	Random forest			X		X			Uses HRV with clinical variables to screen patients at risk of moderate-to-severe OSA.
(Benedetti et al., 2022)	T&F	Wrist-worn smartband signals	OSAS screening	Machine-learning classifiers		X	X				X	Supports smartband-based OSAS screening using wearable physiological and activity features.
(Cai et al., 2024)	T&F	Nasopharyngoscopy images	Adenoid hypertrophy quantification and pediatric OSA severity association	Deep-learning image segmentation/quantification				X	X		X	Quantifies pediatric anatomical obstruction and links adenoid hypertrophy measurements with AHI.
(Chen et al., 2021)	T&F	PPG-based smartwatch signals	OSA screening against PSG	Smartwatch PPG screening algorithm		X	X				X	Provides validation evidence for PPG-smartwatch OSA screening in comparison with PSG.
(Hsu et al., 2026)	T&F	Patch-type ECG/HRV monitoring	Low-interference OSA detection against HSAT	AI-based HRV analysis		X	X				X	Shows feasibility of patch-type ECG/HRV analysis for low-burden home OSA screening.
(Huang et al., 2024)	T&F	Clinical and biochemical indicators	Adult OSA prediction / screening	Machine-learning prediction model					X		X	Supports clinical-biochemical OSA screening using routinely available predictors.
(Kim et al., 2025a)	T&F	Smartwatch signals; AI-scored PSG reference	AHI estimation and OSA assessment	AI-enhanced smartwatch scoring	X	X	X				X	Validates smartwatch-derived AHI estimation against AI-scored and expert-scored PSG.
(Kim et al., 2025c)	T&F	Sleep audio	Apnea event localization, duration estimation, subtype classification, and AHI-related assessment	Whisper-based Transformer audio segmentation		X	X				X	Provides fine-grained audio-based apnea detection with event localization and subtype classification.
(Kuhn et al., 2026)	T&F	Multi-night wrist-worn PPG; sleep diaries	OSA phenotype discovery based on sleep perception	K-means clustering					X		X	Identifies sleep-perception phenotypes in OSA using multi-night wearable and diary data.
(Lai et al., 2025)	T&F	Awake nasopharyngoscopic video	Anatomical OSA detection and airway-feature quantification	Xception; U-Net segmentation; support vector regression				X			X	Supports anatomical OSA detection from awake endoscopic video with quantitative airway-feature extraction.
(Lee et al., 2021)	T&F	Structural/brain connectivity networks	OSA severity and daytime-sleepiness assessment	Deep-learning brain-connectivity model				X	X		X	Uses brain-network learning to relate OSA severity and daytime sleepiness, supporting neuroimaging phenotyping.
(Lee et al., 2025b)	T&F	24-GHz wireless radar respiratory signals	Respiratory-event identification and OSA severity estimation	Deep neural decision trees		X	X				X	Demonstrates contactless radar-based respiratory-event detection and OSA severity screening in home settings.
(Leger and Elbaz, 2024)	T&F	Actigraphy-derived sleep time; RIP belts	Home-based OSA and insomnia assessment; AHI estimation	Automated sleep-state and respiratory analysis	X	X	X				X	Shows that limited home signals can support automated sleep-state estimation and OSA-related AHI assessment.
(Li et al., 2025a)	T&F	Resting-state fMRI; whole-brain functional connectivity	OSA classification and OSA-related cognitive-impairment classification	Feature selection; SVM				X	X		X	Uses individual functional connectivity to classify OSA and cognitive-impairment subgroups.
(Liu et al., 2022)	T&F	SpO_2 desaturation segments; whole-night oximetry features	Event-level SDB screening and severity grading	BiLSTM-CNN feature fusion		X	X				X	Combines desaturation segments and whole-night features to screen and grade SDB from oximetry.

Continued on next page

Ref.	Publisher	Data modality	Clinical / technical task	AI method	G1: PSG/HSAT	G2: wearable/contactless	G3: physiological signals	G4: imaging/anatomy	G5: phenotyping/risk	G6: treatment/monitoring	G7: trustworthy/deployable	Main finding / review relevance
(Liu et al., 2024c)	T&F	Resting-state functional connectivity	OSA classification and cognitive-risk analysis	Connectivity features; SVM				X	X		X	Links altered brain connectivity with moderate-to-severe OSA and supports neuroimaging-based risk stratification.
(Liu et al., 2024b)	T&F	Pediatric clinical and cephalometric variables	Pediatric OSA severity prediction	LASSO regression and nomogram modeling				X	X		X	Develops and validates a pediatric OSA severity prediction nomogram from clinical/anatomical variables.
(Liu et al., 2026a)	T&F	Cephalometric variables; PSG features; clinical indicators	Pediatric OSA screening and anatomical risk assessment	PCA, ROC analysis, and decision-tree modeling				X	X		X	Integrates craniofacial and PSG variables to support pediatric OSA screening and risk modeling.
(Su et al., 2024)	T&F	Cephalometric and craniofacial measurements	Pediatric OSA prediction with external validation	LASSO-based prediction model				X	X		X	Supports externally validated pediatric OSA prediction from craniofacial developmental characteristics.
(Tan et al., 2025)	T&F	Type-IV wearable sleep-monitoring device	Outpatient OSA screening against PSG	AI screening algorithm		X	X				X	Supports AI-assisted outpatient screening using a low-burden wearable type-IV monitoring device.
(Wang et al., 2022)	T&F	Noncontact sleep sounds	Apneic-event detection and AHI estimation	Deep learning audio model		X	X				X	Supports noncontact audio-based OSA event detection and AHI-related assessment from sleep-sound recordings.
(Yue et al., 2021)	T&F	Single-channel nasal pressure airflow	OSA event detection, AHI estimation, and severity classification	Multi-resolution residual network / OSASS	X		X				X	Demonstrates airflow-only deep learning for event detection, AHI estimation, and severity classification.
(Zhang et al., 2025c)	T&F	ECG; SpO_2	OSA detection, AHI consistency, and severity assessment	Multimodal multiscale Transformer			X				X	Shows that ECG–SpO_2 Transformer fusion can support OSA detection and severity assessment across datasets.
(Zhao et al., 2024a)	T&F	Clinical/metabolic variables; liver stiffness; visceral adipose tissue	Severe OSA prediction in patients with obesity	Prediction nomogram / regression-based risk model					X		X	Provides a non-invasive severe-OSA risk model for patients with obesity using metabolic and adiposity indicators.
(Zhou et al., 2023)	T&F	Wearable multi-sensor system; PSG reference	Sleep staging and OSA screening against PSG	AI-assisted wearable sleep analysis	X	X	X				X	Shows that a wearable multi-sensor system can support OSA screening and sleep-stage estimation in comparison with PSG.
(Zhou et al., 2024)	T&F	Smartwatch PPG / wearable sleep signals	OSA screening validation against PSG	Wearable sleep-analyzer algorithm		X	X				X	Validates smartwatch-based OSA screening against PSG and supports consumer-wearable triage applications.
(Attar, 2025)	Frontiers	Single-lead ECG; HRV features	Apnea vs non-apnea epoch classification	Random Forest; XGBoost; statistical HRV analysis			X				X	Uses clinically interpretable time-, frequency-, and nonlinear-domain HRV features to distinguish apnea and non-apnea epochs.
(Gu and Fan, 2025)	Frontiers	EEG signals	AI-enabled OSA screening	Auditory-Linguistic Hierarchical Transformer; contextual/adaptive attention	X		X				X	Uses EEG feature analysis and transformer-style modeling for OSA screening; the unusual auditory-linguistic framing warrants careful methodological appraisal.
(Liu et al., 2026b)	Frontiers	Clinical/questionnaire variables; PSG-labeled cohort	OSA screening and clinical risk prediction	Gradient boosting; visual clinical implementation framework					X		X	Develops and validates a questionnaire-based OSA screening model using retrospective and prospective cohorts, supporting deployable clinical triage.

Continued on next page

Ref.	Publisher	Data modality	Clinical / technical task	AI method	G1: PSG/HSAT	G2: wearable/contactless	G3: physiological signals	G4: imaging/anatomy	G5: phenotyping/risk	G6: treatment/monitoring	G7: trustworthy/deployable	Main finding / review relevance
(Zhang et al., 2025a)	Frontiers	Audio-enabled smartwatch; cough and forced-exhalation sounds; SpO_2; HR/HRV/RR; COPD outpatient clinical data	OSA/overlap-syndrome screening and risk stratification in COPD	Audio feature extraction; predictive modeling		X	X		X		X	Combines smartwatch audio, physiological, and clinical variables for deployable OSA screening and risk stratification in a high-risk COPD population.
(Aswath et al., 2023)	IEEE	Single-lead ECG features	Sleep-apnea detection	Cascaded atrous deep learning and hybrid optimization			X				X	ECG-based OSA detection with optimized feature fusion and deep learning.
(Barroso-García et al., 2025)	IEEE	Single-channel airflow; pediatric recordings	Pediatric sleep-apnea detection	Explainable deep learning	X		X				X	Pediatric airflow-based sleep-apnea detection with explainability.
(Blanchard et al., 2022)	IEEE	Sleep signals and clinical variables	Cardiovascular-risk estimation in sleep-apnea patients	Deep survival learning					X		X	Deep survival cardiovascular-risk estimation in sleep-apnea patients.
(Chen et al., 2024)	IEEE	Wearable bracelet PPG	Energy-efficient sleep-apnea detection	Hyperdimensional computing framework		X	X				X	Wearable bracelet PPG sleep-apnea detection with energy-efficient hyperdimensional computing.
(Choksatchawathi et al., 2024)	IEEE	Fingertip PPG	Sleep-apnea event-onset recognition	Data-driven PPG feature learning		X	X				X	Fingertip PPG/deep-learning OSA event-onset recognition.
(Eris et al., 2025)	IEEE	Pulmonary-function-test flow-volume curves	OSAS diagnosis	AI classifiers and PFT feature selection			X		X		X	PFT flow-volume AI classifier for OSAS diagnosis.
(Faridsoltani et al., 2026)	IEEE	Radar-derived respiratory signals	Noncontact SAHS detection	VMD features and deep neural networks		X	X				X	Radar respiratory-signal noncontact SAHS detection.
(Feng et al., 2021)	IEEE	Single-lead ECG	Sleep-apnea detection	Unsupervised feature learning; sparse autoencoder; cost-sensitive classification			X				X	ECG sleep-apnea detection using unsupervised feature learning.
(Garcia-Vicente et al., 2025)	IEEE	Pediatric overnight ECG	Pediatric OSA diagnosis and severity estimation	Explainable CNN–RNN / Grad-CAM			X				X	Pediatric ECG/XAI model for pediatric OSA diagnosis/severity estimation.
(Gupta et al., 2024)	IEEE	ECG sensor data	Sleep-apnea detection	Multiresolution assessment and wide neural network			X				X	ECG sleep-apnea detection using multiresolution decomposition and wide neural network.
(Han et al., 2026)	IEEE	Tracheal and ambient mobile audio	Apnea/hypopnea event detection and OSA screening	Deep audio learning		X	X				X	Ambient/tracheal audio apnea/hypopnea event detection and OSA screening.
(Hanif et al., 2021)	IEEE	3-D craniofacial scans	AHI estimation and anatomical risk prediction	Deep learning on 3-D facial geometry				X	X		X	3-D craniofacial deep learning for AHI estimation and anatomical risk prediction.
(Hassan et al., 2023)	IEEE	Open-source apnea ECG / respiratory features	Sleep-apnea detection with hardware inference	Shift-accumulate binarized neural network			X				X	Deployable hardware-optimized binarized neural network for sleep-apnea detection.
(Hossan et al., 2025)	IEEE	Single-lead ECG	Sleep-apnea detection	Multistage CNN-BiLSTM / DeepApneaNet			X				X	Cascaded CNN-BiLSTM OSA detection from ECG.
(Hu et al., 2026)	IEEE	Snoring waveform and composite acoustic features	Upper-airway obstructive-site recognition	Multimodal acoustic feature-fusion network			X	X		X	X	Snoring-acoustic upper-airway obstructive-site recognition for treatment/anatomical assessment.
(Huttunen et al., 2023)	IEEE	PSG signal combinations	Joint sleep staging, respiratory-event detection, and AHI assessment	Deep learning with signal-combination comparison	X		X				X	PSG DL for joint sleep staging, respiratory-event detection, and AHI assessment.

Continued on next page

Ref.	Publisher	Data modality	Clinical / technical task	AI method	G1: PSG/HSAT	G2: wearable/contactless	G3: physiological signals	G4: imaging/anatomy	G5: phenotyping/risk	G6: treatment/monitoring	G7: trustworthy/deployable	Main finding / review relevance
(Jiao et al., 2024)	IEEE	Single-lead ECG; R–R intervals; R-peak amplitudes	Sleep-apnea event detection	CNN blocks; multi-head attention; multi-temporal fusion			X				X	ECG-based sleep-apnea event detection using multi-temporal CNN and attention.
(Jirakittayakorn et al., 2024)	IEEE	Type III PSG signals: airflow, effort, SpO_2	AHI estimation and OSA severity classification	Dual CNN/Bi-LSTM networks	X		X				X	Type III PSG event detection, AHI estimation, and OSA severity classification.
(Kamal et al., 2026)	IEEE	EEG and ECG	Sleep staging and OSA screening	Attention-driven multimodal fusion	X		X				X	EEG/ECG multimodal framework for sleep staging and OSA screening.
(Khan et al., 2024)	IEEE	Single-lead EEG	OSA detection	Ensemble learning with DWT features	X		X				X	Single-lead EEG OSA detection using DWT, feature selection, and ensemble learning.
(Khan et al., 2025)	IEEE	Single-lead EEG	Sleep-apnea detection	DWT features and ensemble learning	X		X				X	Single-lead EEG sleep-apnea detection with advanced ML.
(Koda et al., 2024)	IEEE	Noncontact radar with PSG reference	Apnea/hypopnea detection and event-rate estimation	Radar respiratory analysis; expectation–maximization		X	X				X	Radar-based noncontact apnea/hypopnea detection with PSG comparison.
(Kumar et al., 2023)	IEEE	Single-channel ECG	OSA detection	Self-supervised contrastive representation learning			X				X	Single-channel ECG OSA detection using self-supervised/contrastive learning.
(Kwon et al., 2022)	IEEE	IR-UWB radar with PSG reference	Real-time apnea/hypopnea event detection	Hybrid CNN–LSTM		X	X				X	Noncontact IR-UWB radar apnea/hypopnea detection and severity estimation.
(Kwon et al., 2026)	IEEE	Under-mattress PVDF sensor	Personalized unconstrained sleep-apnea detection	Conv-ViT with LoRA tuning		X	X				X	Under-mattress PVDF Conv-ViT + LoRA personalized apnea detection.
(Lee et al., 2025a)	IEEE	Multimodal PSG signals	Sleep staging, apnea detection, and hypopnea detection	SynthSleepNet; hybrid self-supervised learning	X		X				X	Multimodal PSG foundation model for sleep staging, apnea detection, and hypopnea detection.
(Lin et al., 2022b)	IEEE	Single-channel nasal pressure	SAHS assessment	Multi-stage SVM-based classification	X		X				X	Single-channel nasal pressure SAHS assessment.
(Lin et al., 2023)	IEEE	EEG-derived sleep-stage sequences	OSA detection and AHI-related rarity scoring	Rarity-based sleep-stage transition analysis	X						X	EEG/sleep-stage sequence rarity scoring for OSA detection and AHI relation.
(Liu et al., 2026c)	IEEE	Airflow and respiratory-effort signals	OSA/hypopnea event-boundary detection	DFCNet deep-learning architecture	X		X				X	Airflow/respiratory-effort deep learning for OSA/hypopnea event-boundary detection.
(Mahmud et al., 2021)	IEEE	EEG	Sleep-apnea frame detection	VMD, CNN, and BiLSTM	X		X				X	EEG VMD + CNN-BiLSTM sleep-apnea detection.
(Nassi et al., 2022)	IEEE	Single respiratory-effort belt	Respiratory-event scoring and AHI stratification	Deep neural networks	X		X				X	Effort-belt DNN scoring of obstructive apnea, central apnea, hypopnea, and RERAs.
(Nguyen et al., 2024)	IEEE	Single-lead ECG	Sleep-apnea classification	Matrix-profile features and CNN			X				X	Single-lead ECG matrix-profile and CNN sleep-apnea classification.
(Nikkonen et al., 2021)	IEEE	SpO_2, thermistor airflow, nasal pressure, thoracic effort	Respiratory-event scoring and AHI estimation	LSTM neural network	X		X				X	Automatic respiratory-event scoring and AHI estimation from PSG respiratory signals.
(Olsen et al., 2024)	IEEE	Wrist-worn accelerometry and PPG	Sleep staging and sleep-apnea detection	Deep transfer learning		X	X				X	Wrist-worn accelerometry/PPG sleep-apnea detection with external validation.
(Padovano et al., 2022)	IEEE	ECG-derived HRV	External generalization assessment of OSA detectors	ML classifiers and external validation analysis			X				X	Generalization/external-validation study for HRV-based OSA detectors.

Continued on next page

Ref.	Publisher	Data modality	Clinical / technical task	AI method	G1: PSG/HSAT	G2: wearable/contactless	G3: physiological signals	G4: imaging/anatomy	G5: phenotyping/risk	G6: treatment/monitoring	G7: trustworthy/deployable	Main finding / review relevance
(Qiu et al., 2025)	IEEE	Wearable PPG bracelet	Sleep-apnea detection	Ordinal-pattern supervised prototype contrastive learning		X	X				X	Bracelet PPG sleep-apnea detection with supervised prototype contrastive learning.
(Romero et al., 2022)	IEEE	Smartphone whole-night breathing sounds	Home OSA screening and AHI estimation	Deep neural networks for acoustic screening		X	X				X	Smartphone/whole-night breathing audio OSA screening and AHI-related estimation.
(Seo et al., 2023)	IEEE	Single-lead ECG	AHI and OSA severity estimation	WASO-aware deep neural network			X				X	ECG-based apnea/hypopnea, sleep-state, AHI, and severity estimation.
(Shao et al., 2022)	IEEE	ECG/HRV features and time-frequency representations	OSA detection for IoMT workflows	Parallel heterogeneous deep-learning model			X				X	HRV/ECG time-frequency IoMT-supported OSA detection.
(Sillaparaya et al., 2025)	IEEE	Snore sounds; Mel-spectrograms; MFCCs	OSA classification	Attention-based transfer learning		X	X				X	Audio/snore-sound OSA classification with transfer learning and attention.
(Sinha and Das, 2022)	IEEE	Single-lead ECG	Minute-level OSA detection	NMF features in eigen-spectrum domain; ensemble learning			X				X	ECG singular-spectrum/NMF and ensemble OSA detection.
(TaghiBeyglou et al., 2026)	IEEE	Wakefulness speech/vowel recordings	OSA severity prediction	Self-supervised speech representations and model fusion			X		X		X	Wakefulness speech/vowel self-supervised representations for OSA severity prediction.
(Tang et al., 2026)	IEEE	ECG scalograms	OSA detection	Hybrid residual neural network			X				X	ECG scalogram residual neural-network OSA detection.
(Thompson et al., 2024)	IEEE	Single-lead ECG time-series segments	OSA detection	1D CNN			X				X	ECG-based OSA detection with 1D-CNN.
(Tian et al., 2026)	IEEE	Airflow, thoracoabdominal effort, SpO_2	Apnea/hypopnea detection	Heterogeneity-aware multimodal cross-epoch network	X		X				X	Airflow/effort/SpO2 multimodal cross-epoch apnea/hypopnea detection.
(Wan et al., 2025)	IEEE	EEG	Sleep-stage classification and OSA prediction	Transformer-based sequential EEG model	X		X				X	EEG transformer-style model for sleep-stage classification and OSA prediction.
(Wang et al., 2021)	IEEE	EEG	Hypopnea/apnea event detection	Wavelet and entropy features; ML classifiers	X		X				X	EEG-based apnea/hypopnea event detection using wavelet/entropy features.
(Wang et al., 2024)	IEEE	ECG	OSA detection under limited/imbalanced data	ECGAN-assisted ResT-Net with fuzziness handling			X				X	ECG-based OSA detection with ECGAN sample generation and fuzziness-aware ResT-Net.
(Wang et al., 2025)	IEEE	EEG, ECG, airflow, and EOG-guided PSG signals	Joint OSA detection and sleep staging	Multi-task triple atrous spatial pyramid pooling network	X		X				X	Joint OSA detection and sleep-staging model.
(Wang et al., 2026b)	IEEE	Multichannel PSG	Multitask sleep-event monitoring and apnea detection	PSG masked autoencoder; inter-channel contrastive learning	X		X				X	Self-supervised multichannel PSG model for sleep-event monitoring and sleep-apnea detection.
(Weng et al., 2022)	IEEE	ECG-derived HRV	OSA and severe-OSA screening	Fuzzy approximate entropy and ML screening			X		X		X	ECG/HRV entropy-based OSA and severe-OSA screening.

Continued on next page

Ref.	Publisher	Data modality	Clinical / technical task	AI method	G1: PSG/HSAT	G2: wearable/contactless	G3: physiological signals	G4: imaging/anatomy	G5: phenotyping/risk	G6: treatment/monitoring	G7: trustworthy/deployable	Main finding / review relevance
(Werthen-Brabants et al., 2025)	IEEE	Sleep-study respiratory events	AHI estimation by event counting	Recursive spiking neural networks	X		X				X	AHI estimation paper using event counting and recursive spiking neural networks.
(Wu et al., 2021c)	IEEE	CBCT upper-airway images	Upper-airway segmentation for OSA analysis	3D U-Net with weak-feature-region attention				X			X	CBCT upper-airway segmentation/modeling for OSA analysis.
(Xue et al., 2025)	IEEE	Wakefulness speech; articulatory and spectral features	OSA severity estimation	Knowledge-guided articulatory/spectrum fusion network			X		X		X	Wakefulness speech/articulatory and spectral fusion for OSA severity estimation.
(Ye et al., 2021)	IEEE	RR-interval / PPG-compatible wearable signals	OSA detection	Frequency extraction network		X	X				X	Wearable RR-interval/PPG-compatible energy-efficient OSA detection.
(Yeo et al., 2022)	IEEE	Single-lead ECG; open and patch-wearable datasets	Sleep-apnea screening	Deep residual network		X	X				X	Single-lead ECG screening validated with patch wearable data.
(Yoo et al., 2026)	IEEE	2-D facial images, cephalometry, and clinical data	Moderate-to-severe OSA prediction	Multimodal deep-learning prediction model				X	X		X	Facial/cephalometric/clinical deep-learning model for moderate-to-severe OSA screening.
(Zan et al., 2025)	IEEE	PSG-derived respiratory and physiological signals	Sleep-apnea severity monitoring from coarse labels	Weakly supervised deep learning	X		X				X	Weakly supervised PSG/physiological severity monitoring from coarse labels.
(Zhang et al., 2023b)	IEEE	Bed-integrated RF respiratory sensing	Sleep-disorder event detection and prediction	Random forest and RF sensing features		X	X				X	Bed-integrated RF sensing for respiratory-event detection and prediction.
(Zhang et al., 2023a)	IEEE	Dual-channel flexible nasal respiratory sensors	Long-term sleep respiratory monitoring	1D-CNN aided signal analysis		X	X				X	Wearable nasal respiratory sensors and 1D-CNN for long-term OSAHS-oriented respiratory monitoring.
(Zhang et al., 2025d)	IEEE	Single-channel airflow	Real-time sleep-apnea detection and prediction	Deterministic learning and dynamical feature modeling	X		X				X	Single-channel airflow real-time sleep-apnea detection and prediction.
(Zhang et al., 2026b)	IEEE	SpO_2 desaturation and reoxygenation events	OSA classification and severity stratification	Event-based oximetry metrics; ML and SHAP			X		X		X	Interpretable SpO2 desaturation/reoxygenation metrics with ML and SHAP.
(Zhao et al., 2024c)	IEEE	Single-lead ECG	Automated sleep-apnea detection	Dual-multiscale interactive attention CNN			X				X	ECG sleep-apnea detection using multiscale interactive attention CNN.
(Zhao et al., 2024b)	IEEE	Flexible tactile sensor / bed-integrated signals	Unconstrained respiratory-event detection	Deep learning for tactile respiratory signals		X	X				X	Bed-embedded tactile sensor and temporal convolutional DL for respiratory-event detection.
(Zou and Liu, 2024)	IEEE	Wearable PPG bracelet	Sleep-apnea detection	Multiscale bidirectional temporal convolutional network		X	X				X	Wearable PPG bracelet sleep-apnea detection using multiscale bidirectional temporal convolutional modeling.

Table S4: Study-level methodological characteristics, validation strategies, performance reporting, and descriptive quality-appraisal indicators across the 218 core AI-for-SDB studies. This evidence map is not a formal QUADAS-2 or PROBAST risk-of-bias assessment.

**Study**	**Dataset/cohort**	**Unit**	**Split/partition**	**CV**	**Validation**	**Reference standard**	**Comparator**	**Principal reported performance**	**Main methodological limitation; data/code**
(Almutairi and Hassan, 2021)	PhysioNet Apnea-ECG; 35 recordings; 32 subjects	Segment or epoch, Participant	Allocation unit NR; internal holdout	10-fold cross validation	Internal hold-out; partition unit unclear	PSG/AHI	SVM	While for the testing dataset, the achieved accuracy, sensitivity, specificity and F1 score for the proposed model are 90.92%, 91.24%, 90.36% and 92.76% respectively.	Allocation unit NR; No strict external test identified; Data/code: NR
(Bernardini et al., 2021)	PhysioNet Apnea-ECG; 25 patients; 77 patients; 18 patients	Event, Segment or epoch, Participant	Subject or recording separated; internal holdout	10-fold cross-validation	Internal subject-separated hold-out	Expert or manual scoring	Logistic regression; LSTM; Neural network	For per-patient OSA classification, multimodal CNN–LSTM analysis of ECG and SpO_2 reported sensitivity 1.00, specificity 0.714, accuracy 0.933, and F1 0.958.	No strict external test identified; Data/code: NR
(Bricout et al., 2021)	PhysioNet Apnea-ECG; 13 subjects; 28 subjects; 15 subjects	Event, Segment or epoch, Participant	Allocation unit NR	NR	Validation strategy not clearly reported or not applicable	Expert or manual scoring	NR	At a 300-s epoch, screening accuracy was 100% using the PSG configuration and 89% using the accelerometry-derived respiratory configuration.	Allocation unit NR; No strict external test identified; Data/code: NR
(Chen et al., 2021)	102 participants; 20 participants; 82 participants; 83 participants	Event, Recording or night, Participant	Allocation unit NR	NR	Validation strategy not clearly reported or not applicable	PSG/AHI; Expert or manual scoring	NR	Reported metrics: Accuracy; Specificity; AUC/AUROC; Kappa; Correlation/R2. See source study for task-specific values.	Allocation unit NR; No strict external test identified; Data/code: NR
(Dietz-Terjung et al., 2021)	MASS; 128 patients	Segment or epoch, Participant	Study/recording-level development data plus independent clinical cohort; internal holdout	5-fold cross validations	External clinical validation of a previously developed model	PSG/AHI; Expert or manual scoring	Neural network	Sleep/wake agreement had Cohen's kappa 0.62, and estimated versus manually scored AHI had correlation $r = 0.91$.	Data/code: NR
(Faust et al., 2021)	PhysioNet Apnea-ECG; 35 recordings	Segment or epoch	Allocation unit NR; internal holdout	cross-validation	Internal hold-out; partition unit unclear	PSG/AHI	LSTM	LSTM hold-out accuracy, sensitivity, and specificity were 81.30%, 59.90%, and 91.75%; corresponding 10-fold-CV values were 99.80%, 99.85%, and 99.73%.	Allocation unit NR; No strict external test identified; Data/code: NR

Continued on next page

Table S4 continued

Study	Dataset/cohort	Unit	Split/partition	CV	Validation	Reference standard	Comparator	Principal reported performance	Main methodological limitation; data/code
(Feng et al., 2021)	MESA Sleep; 14 subjects; 21 subjects	Event, Segment or epoch, Recording or night	Allocation unit NR; internal holdout	NR	Internal hold-out; partition unit unclear	PSG/AHI	NR	Reported metrics: Accuracy; Sensitivity; Recall; MAE. See source study for task-specific values.	Allocation unit NR; No strict external test identified; Data/code: NR
(Hanif et al., 2021)	100 subjects; 69 subjects; 1,366 subjects	Segment or epoch, Participant, Image or region	Allocation unit NR; internal holdout	10-fold cross-validation	Internal hold-out; partition unit unclear	PSG/AHI; Expert or manual scoring	NR	Reported metrics: Accuracy; Sensitivity; AUC/AUROC; Kappa; MAE; Bland-Altman agreement. See source study for task-specific values.	Allocation unit NR; No strict external test identified; Data/code: NR
(Ivanovska et al., 2021)	181 subjects; 18 subjects	Segment or epoch, Image or region	Allocation unit NR; internal holdout	cross-validation	Internal hold-out; partition unit unclear	Expert or manual scoring	NR	Reported metrics: Accuracy. See source study for task-specific values.	Allocation unit NR; No strict external test identified; Data/code: NR
(Iwasaki et al., 2021)	PhysioNet Apnea-ECG; N = 35	Segment or epoch, Participant	Allocation unit NR; internal holdout	5-fold cross-validation	Internal hold-out; partition unit unclear	PSG/AHI	NR	Reported metrics: Sensitivity; Specificity; Correlation/R2. See source study for task-specific values.	Allocation unit NR; No strict external test identified; Code available
(Jiang et al., 2021)	6 subjects	Segment or epoch, Participant	Allocation unit NR	cross validation	Cross-validation; partition unit unclear	Expert or manual scoring	Logistic regression; SVM; KNN	Across five classifiers, overall accuracy exceeded 90% and sensitivity was 100% when using the selected six acoustic features.	Allocation unit NR; No strict external test identified; Data/code: NR
(Juang et al., 2021)	Cohort size NR	Segment or epoch, Participant	Segment/epoch split; subject separation NR	NR	Validation strategy not clearly reported or not applicable	PSG/AHI	NR	Reported metrics: Accuracy; Sensitivity. See source study for task-specific values.	Participant separation not established; No strict external test identified; Data/code: NR
(Lee et al., 2021)	Cohort details NR	NR	Allocation unit NR; internal holdout	NR	Internal hold-out; partition unit unclear	Expert or manual scoring	NR	Reported metrics: Correlation/R2. See source study for task-specific values.	Allocation unit NR; No strict external test identified; Data/code: NR
(Leino et al., 2021)	Sleep Heart Health Study (SHHS); cohort size NR	Event, Segment or epoch, Participant	Allocation unit NR; internal holdout	NR	Internal hold-out; partition unit unclear	PSG/AHI; Expert or manual scoring	CNN	Reported metrics: Sensitivity; Correlation/R2. See source study for task-specific values.	Allocation unit NR; No strict external test identified; Data/code: NR
(Mahmud et al., 2021)	UCDDB; CAP Sleep Database; Cohort size NR	Segment or epoch	Subject-dependent/overlap	cross-validation	Internal segment/subject-overlap evaluation	Expert or manual scoring	NR	Reported metrics: Sensitivity. See source study for task-specific values.	Subject overlap/dependence; No strict external test identified; Data/code: NR

Continued on next page

Table S4 continued

Study	Dataset/cohort	Unit	Split/partition	CV	Validation	Reference standard	Comparator	Principal reported performance	Main methodological limitation; data/code
(Mashrur et al., 2021)	PhysioNet Apnea-ECG; 35 subjects; 31 subjects; 70 subjects	Segment or epoch, Recording or night, Participant	Segment/epoch split; subject separation NR; internal holdout	NR	Internal segment/subject-overlap evaluation	PSG/AHI	CNN	Test accuracy, sensitivity, and specificity were 94.38%, 94.30%, and 94.51%, respectively.	Participant separation not established; No strict external test identified; Data/code: NR
(Nikkonen et al., 2021)	787 patients; 100 patients	Event, Segment or epoch	Segment/epoch split; subject separation NR; internal holdout	NR	Internal segment/subject-overlap evaluation	Expert or manual scoring	NR	Agreement between neural-network and manual AHI had ICC 0.985 (95% CI 0.978–0.990).	Participant separation not established; No strict external test identified; Data/code: NR
(Pan et al., 2021)	15 patients; 6 patients; 14 patients; 21 patients	Event, Segment or epoch, Recording or night	Allocation unit NR; internal holdout	Leave-one-out	Internal hold-out; partition unit unclear	PSG/AHI	NR	The best binary classifier reported accuracy 98.8%, recall 100%, specificity 97.6%, precision 97.7%, and AUC 0.997.	Allocation unit NR; No strict external test identified; Data/code: NR
(Rajesh et al., 2021)	PhysioNet Apnea-ECG; cohort size NR	Event, Segment or epoch	Allocation unit NR; internal holdout	10-fold cross-validation	Internal hold-out; partition unit unclear	PSG/AHI	SVM; Random forest	Accuracy was 89% with 50:50 hold-out validation and 90% with 10-fold cross-validation.	Allocation unit NR; No strict external test identified; Data/code: NR
(Ryu et al., 2021)	15 patients	Segment or epoch, Image or region	Allocation unit NR; internal holdout	cross-validation	Internal hold-out; partition unit unclear	Expert or manual scoring	SVM	Classification accuracy, sensitivity, specificity, and F1 were 81.5%, 89.3%, 86.2%, and 87.6%, respectively.	Allocation unit NR; No strict external test identified; Data/code: NR
(Taghizadegan et al., 2021)	St. Vincent's University Hospital dataset; 10 participants	Segment or epoch	Subject-dependent/overlap; internal holdout	leave-one-subject-out; 10-fold cross-validation	Internal segment/subject-overlap evaluation	PSG/AHI	NR	Reported metrics: Accuracy; Sensitivity; Specificity; Precision; AUC/AUROC. See source study for task-specific values.	Subject overlap/dependence; No strict external test identified; Data/code: NR
(Tang and Liu, 2021)	PhysioNet Apnea-ECG; 32 subjects	Segment or epoch, Participant	Allocation unit NR; internal holdout	five-fold cross-validation	Internal hold-out; partition unit unclear	Expert or manual scoring	NR	Reported metrics: Correlation/R2. See source study for task-specific values.	Allocation unit NR; No strict external test identified; Data/code: NR
(Tsuiki et al., 2021)	MASS; n = 1101; n = 1151; n = 1158; n = 66; n = 867; n = 522	Event, Segment or epoch, Image or region	Allocation unit NR	NR	Validation strategy not clearly reported or not applicable	PSG/AHI	NR	Reported metrics: Sensitivity; Specificity; AUC/AUROC. See source study for task-specific values.	Allocation unit NR; No strict external test identified; Data on request
(Wang et al., 2021)	30 subjects	Event, Segment or epoch, Participant	Segment/epoch split; subject separation NR; internal holdout	NR	Internal segment/subject-overlap evaluation	NR	SVM	Accuracy, sensitivity, and specificity were 94.33%, 93.10%, and 95.07%, respectively.	Participant separation not established; No strict external test identified; Data/code: NR

Continued on next page

Table S4 continued

Study	Dataset/cohort	Unit	Split/partition	CV	Validation	Reference standard	Comparator	Principal reported performance	Main methodological limitation; data/code
(Wu et al., 2021d)	500 subjects	Event, Segment or epoch, Participant	Allocation unit NR; internal holdout	NR	Internal hold-out; partition unit unclear	Expert or manual scoring	NR	Reported metrics: Accuracy; Sensitivity. See source study for task-specific values.	Allocation unit NR; No strict external test identified; Data/code: NR
(Wu et al., 2021b)	20 recordings; 10 recordings; 60 recordings	Segment or epoch, Participant	Allocation unit NR	NR	Validation strategy not clearly reported or not applicable	NR	NR	Reported metrics: Accuracy; Sensitivity; Specificity. See source study for task-specific values.	Allocation unit NR; No strict external test identified; Data/code: NR
(Wu et al., 2021a)	29 subjects; 39 subjects; 24 subjects	Segment or epoch, Participant	Allocation unit NR	NR	Validation strategy not clearly reported or not applicable	PSG/AHI	NR	Reported metrics: Correlation/R2. See source study for task-specific values.	Allocation unit NR; No strict external test identified; Data/code: NR
(Wu et al., 2021c)	Cohort size NR	Segment or epoch, Image or region	Allocation unit NR; internal holdout	NR	Internal hold-out; partition unit unclear	Expert or manual scoring	NR	Reported metrics: Precision; Recall. See source study for task-specific values.	Allocation unit NR; No strict external test identified; Data/code: NR
(Ye et al., 2021)	PhysioNet Apnea-ECG; 243 recordings	Event, Segment or epoch, Participant	Allocation unit NR; internal holdout	NR	Internal hold-out; partition unit unclear	Expert or manual scoring	CNN	Reported metrics: Accuracy; Recall. See source study for task-specific values.	Allocation unit NR; No strict external test identified; Data/code: NR
(Yue et al., 2021)	PhysioNet Apnea-ECG; MESA Sleep; MIT-BIH; 45 patients	Event, Segment or epoch, Participant	Allocation unit NR; internal holdout	NR	Independent-institution external test	PSG/AHI; Expert or manual scoring	SVM; CNN	In the primary test set, sensitivity, specificity, and accuracy were 90.8%, 90.5%, and 91.2%, respectively.	Allocation unit NR; Data/code: NR
(Balci et al., 2022)	19 patients; 15 patients	Event, Segment or epoch, Participant	Segment/epoch split; subject separation NR; internal holdout	10-fold cross validation	Internal segment/subject-overlap evaluation	PSG/AHI	NR	Reported metrics: Accuracy; AUC/AUROC. See source study for task-specific values.	Participant separation not established; No strict external test identified; Data/code: NR
(Benedetti et al., 2022)	78 participants; 2 participants; 76 participants; 20 participants	Participant	Allocation unit NR	Leave-One-Out	Cross-validation; partition unit unclear	Expert or manual scoring	NR	Reported metrics: Sensitivity; Specificity; Correlation/R2. See source study for task-specific values.	Allocation unit NR; No strict external test identified; Data/code: NR
(Bhalerao and Pachori, 2022)	St. Vincent's University Hospital dataset; MIT-BIH; 5 subjects	Event, Segment or epoch, Participant	Subject-dependent/overlap	cross-validation	Internal segment/subject-overlap evaluation	NR	CNN	Subject-independent classification accuracies were 96.24% and 95.86% for the two evaluated settings.	Subject overlap/dependence; No strict external test identified; Data/code: NR
(Blanchard et al., 2022)	383 patients; 2,008 patients; 230 patients; 916 patients; 1,446 patients; 5,506 patients	Event, Segment or epoch	Allocation unit NR; internal holdout	NR	Internal hold-out; partition unit unclear	PSG/AHI; Expert or manual scoring	CNN	Reported metrics: Sensitivity; AUC/AUROC. See source study for task-specific values.	Allocation unit NR; No strict external test identified; Data/code: NR

Continued on next page

Table S4 continued

Study	Dataset/cohort	Unit	Split/partition	CV	Validation	Reference standard	Comparator	Principal reported performance	Main methodological limitation; data/code
(Chen et al., 2022)	PhysioNet Apnea-ECG; 35 recordings; 57 recordings	Event, Segment or epoch, Recording or night	Allocation unit NR; internal holdout	NR	Internal hold-out; partition unit unclear	Expert or manual scoring	NR	Reported metrics: F1; AUC/AUROC. See source study for task-specific values.	Allocation unit NR; No strict external test identified; Code available
(Cheng et al., 2022)	33 patients	Event, Segment or epoch, Recording or night	Allocation unit NR; internal holdout	NR	Internal hold-out; partition unit unclear	PSG/AHI	LSTM	Reported metrics: Accuracy; Sensitivity; Specificity; Precision; F1. See source study for task-specific values.	Allocation unit NR; No strict external test identified; Data/code: NR
(Jimenez-Garcia et al., 2022)	PhysioNet Apnea-ECG; 1,638 recordings; 974 recordings	Event, Segment or epoch, Participant	Subject or recording separated; internal holdout	NR	Independent-site test with external-site data also used for tuning	Clinical/AHI-derived labels	CNN	Reported metrics: Accuracy; Sensitivity; Specificity; Correlation/R2; Bland-Altman agreement. See source study for task-specific values.	Data/code: NR
(Holfinger et al., 2022)	Sleep Heart Health Study (SHHS); National Sleep Research Resource; 1,618 subjects	Event, Participant	Segment/epoch split; subject separation NR; internal holdout	NR	Prospective clinical validation	PSG/AHI; Expert or manual scoring	Logistic regression; Random forest; Neural network; STOP-BANG	Prospective SAGIC ANN/RF AUCs were 0.70 versus 0.71 for STOP-BANG; in SHHS, the ANN and STOP-BANG both had AUC 0.72.	Participant separation not established; Data/code: NR
(Kwon et al., 2022)	4 patients	Event, Segment or epoch, Recording or night	Segment/epoch split; subject separation NR; internal holdout	6-fold cross-validation	Internal segment/subject-overlap evaluation	PSG/AHI; Expert or manual scoring	CNN; LSTM	Therefore, the proposed method showed a Cohen's kappa coefficient of 0.728, sensitivity of 0.781, specificity of 0.956, and an accuracy of 0.930.	Participant separation not established; No strict external test identified; Data/code: NR
(Lin et al., 2022b)	16 subjects; 58 subjects	Event, Segment or epoch, Participant	Segment/epoch split; subject separation NR; internal holdout	NR	Internal segment/subject-overlap evaluation	Expert or manual scoring	NR	Reported metrics: Accuracy; Kappa. See source study for task-specific values.	Participant separation not established; No strict external test identified; Data/code: NR
(Lin et al., 2022a)	32 patients	Event, Segment or epoch, Participant	Segment/epoch split; subject separation NR	NR	Validation strategy not clearly reported or not applicable	PSG/AHI	LSTM	Results showed that through the model pruning, with the tradeoff of losing 0.35% accuracy, the number of neural network parameters was reduced by 40.27%.	Participant separation not established; No strict external test identified; Data/code: NR
(Liu et al., 2022)	Sleep Heart Health Study (SHHS); MASS; 5793 participants; 2651 participants	Event, Segment or epoch, Recording or night	Segment/epoch split; subject separation NR; internal holdout	NR	Cross-dataset and cross-institution evaluation	PSG/AHI	CNN; LSTM	Segment-level accuracy was 84.3%; mean accuracy for SDB at AHI $\geq$15 was 88.95%.	Participant separation not established; Data/code: NR

Continued on next page

Table S4 continued

Study	Dataset/cohort	Unit	Split/partition	CV	Validation	Reference standard	Comparator	Principal reported performance	Main methodological limitation; data/code
(Monna et al., 2022)	2022 participants	Participant, Image or region	Allocation unit NR; internal holdout	Leave-One-Out; LOOCV	Internal hold-out; partition unit unclear	PSG/AHI	NR	Reported metrics: Accuracy; AUC/AUROC. See source study for task-specific values.	Allocation unit NR; No strict external test identified; Data/code: NR
(Nassi et al., 2022)	96 patients	Event, Segment or epoch	Segment/epoch split; subject separation NR; internal holdout	5-fold cross-validation	Cross-dataset external validation	Expert or manual scoring	NR	AUROC/AUPRC were 0.93/0.71 in MGH and 0.92/0.56 in the external SHHS cohort.	Participant separation not established; Data/code: NR
(Padovano et al., 2022)	PhysioNet Apnea-ECG; MIT-BIH; 35 recordings	Segment or epoch, Participant	Segment/epoch split; subject separation NR; internal holdout	cross-validation	Cross-database external validation	PSG/AHI; Expert or manual scoring	NR	External-validation accuracy, sensitivity, and specificity were 20–40% lower than epoch-wise cross-validation results.	Participant separation not established; Data/code: NR
(Qin and Liu, 2022)	PhysioNet Apnea-ECG; 23 recordings; 12 recordings; 74 recordings	Segment or epoch, Recording or night	Segment/epoch split; subject separation NR; internal holdout	cross-validation	Internal segment/subject-overlap evaluation	Expert or manual scoring	NR	Reported metrics: Accuracy; Sensitivity; Precision; Recall; F1; AUC/AUROC. See source study for task-specific values.	Participant separation not established; No strict external test identified; Data/code: NR
(Romero et al., 2022)	17 participants; 8 participants; 5 participants	Segment or epoch, Recording or night, Participant	Allocation unit NR; internal holdout	NR	Internal hold-out; partition unit unclear	HSAT or polygraphy	NR	Reported metrics: Sensitivity. See source study for task-specific values.	Allocation unit NR; No strict external test identified; Data/code: NR
(Shao et al., 2022)	PhysioNet Apnea-ECG; Cohort size NR	Segment or epoch, Participant	Allocation unit NR; internal holdout	cross-validation	Internal hold-out; partition unit unclear	PSG/AHI	NR	Reported metrics: Accuracy; Sensitivity. See source study for task-specific values.	Allocation unit NR; No strict external test identified; Data/code: NR
(Sharma et al., 2022)	Sleep Heart Health Study (SHHS); National Sleep Research Resource; 988 recordings; 8,000 recordings	Event, Segment or epoch, Participant	Subject-dependent/overlap; internal holdout	10-fold cross-validation	Internal segment/subject-overlap evaluation	Expert or manual scoring	NR	Reported metrics: Accuracy. See source study for task-specific values.	Subject overlap/dependence; No strict external test identified; Data/code: NR
(Sinha and Das, 2022)	PhysioNet Apnea-ECG; St. Vincent's University Hospital dataset; 25 subjects	Segment or epoch, Recording or night	Subject or recording separated	NR	Validation strategy not clearly reported or not applicable	PSG/AHI	NR	Reported metrics: Accuracy; Correlation/R2. See source study for task-specific values.	No strict external test identified; Data/code: NR
(Wang et al., 2022)	162 participants; additional reported cohorts of 135 and 59 participants	Event, Segment or epoch, Participant	Allocation unit NR; internal holdout	NR	Temporal/later-enrolled validation	PSG/AHI	NR	Reported metrics: Accuracy; Sensitivity; Specificity. See source study for task-specific values.	Allocation unit NR; Data/code: NR

Continued on next page

Table S4 continued

Study	Dataset/cohort	Unit	Split/partition	CV	Validation	Reference standard	Comparator	Principal reported performance	Main methodological limitation; data/code
(Weng et al., 2022)	UCDDB; 81 patients; 83 patients	Event, Segment or epoch, Participant	Allocation unit NR	cross-validation	Cross-validation; partition unit unclear	PSG/AHI; Expert or manual scoring	Random forest	Reported metrics: Accuracy; Sensitivity; AUC/AUROC. See source study for task-specific values.	Allocation unit NR; No strict external test identified; Data/code: NR
(Yang et al., 2022)	PhysioNet Apnea-ECG; Cohort size NR	Segment or epoch, Recording or night	Allocation unit NR; internal holdout	NR	Additional-dataset retraining (not strict external)	PSG/AHI	CNN	Reported metrics: Accuracy; Sensitivity; Specificity; F1; AUC/AUROC. See source study for task-specific values.	Allocation unit NR; No strict external test identified; Data/code: NR
(Yeo et al., 2022)	MESA Sleep; UCDDB; 70 subjects; 25 subjects; 94 subjects	Event, Segment or epoch, Participant	Segment/epoch split; subject separation NR; internal holdout	NR	Cross-dataset out-of-domain testing	PSG/AHI; Expert or manual scoring	NR	Reported metrics: Kappa; Correlation/R2. See source study for task-specific values.	Participant separation not established; Data/code: NR
(Yu et al., 2022)	N = 6	Event, Segment or epoch, Recording or night	Allocation unit NR	NR	Validation strategy not clearly reported or not applicable	Expert or manual scoring	LSTM; Neural network	Reported metrics: Accuracy; Sensitivity. See source study for task-specific values.	Allocation unit NR; No strict external test identified; Data/code: NR
(Zarei et al., 2022)	PhysioNet Apnea-ECG; UCDDB; 35 recordings	Event, Segment or epoch, Recording or night	Subject or recording separated	NR	Validation strategy not clearly reported or not applicable	NR	CNN; LSTM	Reported metrics: Accuracy; Sensitivity; Specificity. See source study for task-specific values.	No strict external test identified; Data/code: NR
(Aswath et al., 2023)	PhysioNet Apnea-ECG; UCDDB; 859 subjects	Segment or epoch, Participant	Allocation unit NR; internal holdout	NR	Internal hold-out; partition unit unclear	PSG/AHI	LSTM; Neural network	Reported metrics: Accuracy; Sensitivity; Precision. See source study for task-specific values.	Allocation unit NR; No strict external test identified; Data/code: NR
(Bark et al., 2023)	1,983 patients; 994 subjects	Segment or epoch, Participant	Allocation unit NR; internal holdout	NR	Internal hold-out; partition unit unclear	PSG/AHI	NR	Reported metrics: Accuracy; Sensitivity; Specificity; F1. See source study for task-specific values.	Allocation unit NR; No strict external test identified; Code available
(Bokov et al., 2023)	234 children; 156 children; 78 children	Event, Participant	Allocation unit NR; internal holdout	5-fold cross-validation	Temporal/later-enrolled validation	Expert or manual scoring	Logistic regression	Reported metrics: Accuracy; Sensitivity; AUC/AUROC. See source study for task-specific values.	Allocation unit NR; Data/code: NR
(Chen et al., 2023b)	106 subjects (63 training, 16 validation, 27 testing)	Event, Segment or epoch, Recording or night	Segment/epoch split; subject separation NR; internal holdout	NR	Internal segment/subject-overlap evaluation	Expert or manual scoring	Neural network	Reported metrics: Accuracy; Sensitivity; MAE; Correlation/R2. See source study for task-specific values.	Participant separation not established; No strict external test identified; Data/code: NR

Continued on next page

Table S4 continued

Study	Dataset/cohort	Unit	Split/partition	CV	Validation	Reference standard	Comparator	Principal reported performance	Main methodological limitation; data/code
(Chen et al., 2023a)	Sleep Heart Health Study (SHHS); MESA Sleep; MrOS Sleep; 5,989 recordings; 8,444 recordings	Event, Segment or epoch, Participant	Allocation unit NR; internal holdout	NR	Cross-dataset generalization	PSG/AHI	NR	Reported metrics: Accuracy; Sensitivity; Specificity; Precision. See source study for task-specific values.	Allocation unit NR; Data/code: NR
(Cheng et al., 2023)	UCDDB; ISRUC-Sleep; 61 subjects	Event, Segment or epoch, Recording or night	Subject or recording separated; internal holdout	cross validation	Cross-database validation	Expert or manual scoring	NR	Reported metrics: Accuracy; Sensitivity; Specificity. See source study for task-specific values.	Data/code: NR
(Bahr-Hamm et al., 2023)	27 patients; 21 patients; 23 patients; 15 patients	Event, Segment or epoch, Recording or night	Segment/epoch split; subject separation NR	NR	Validation strategy not clearly reported or not applicable	PSG/AHI; Expert or manual scoring	SVM	Reported metrics: AUC/AUROC. See source study for task-specific values.	Participant separation not established; No strict external test identified; Data/code: NR
(Hanif et al., 2023)	Cohort size NR	Segment or epoch, Participant, Image or region	Segment/epoch split; subject separation NR	NR	Validation strategy not clearly reported or not applicable	Expert or manual scoring	NR	Reported metrics: Accuracy; Sensitivity; F1. See source study for task-specific values.	Participant separation not established; No strict external test identified; Data/code: NR
(Hassan et al., 2023)	PhysioNet Apnea-ECG; 25 subjects	Segment or epoch, Participant	Allocation unit NR; internal holdout	cross-validation	Internal hold-out; partition unit unclear	PSG/AHI	NR	Reported metrics: Accuracy; Precision. See source study for task-specific values.	Allocation unit NR; No strict external test identified; Data/code: NR
(He et al., 2023)	MASS; N = 371; N = 53; N = 106; 1 Participant; 78 Participants; 71 Participants	Event, Segment or epoch, Participant	Allocation unit NR; internal holdout	NR	Internal hold-out; partition unit unclear	PSG/AHI	NR	Reported metrics: Accuracy; Sensitivity; Specificity; AUC/AUROC. See source study for task-specific values.	Allocation unit NR; No strict external test identified; Data/code: NR
(Huttunen et al., 2023)	n = 710; n = 79; n = 88	Event, Segment or epoch, Recording or night	Subject or recording separated; internal holdout	NR	Internal subject-separated hold-out	PSG/AHI; Expert or manual scoring	NR	Reported metrics: Accuracy; Precision; Recall; MAE. See source study for task-specific values.	No strict external test identified; Code available
(Javeed et al., 2023)	Cohort size NR	Participant	Allocation unit NR; internal holdout	cross-validation	Internal hold-out; partition unit unclear	PSG/AHI	LSTM	Reported metrics: Accuracy; Sensitivity; F1; Correlation/R2. See source study for task-specific values.	Allocation unit NR; No strict external test identified; Data/code: NR
(Kumar et al., 2023)	PhysioNet Apnea-ECG; UCDDB; 35 recordings	Event, Segment or epoch, Recording or night	Segment/epoch split; subject separation NR; internal holdout	ten-fold cross-validation	Internal segment/subject-overlap evaluation	Expert or manual scoring	NR	Reported metrics: Accuracy; Sensitivity; Recall; F1. See source study for task-specific values.	Participant separation not established; No strict external test identified; Data/code: NR

Continued on next page

Table S4 continued

Study	Dataset/cohort	Unit	Split/partition	CV	Validation	Reference standard	Comparator	Principal reported performance	Main methodological limitation; data/code
(Li et al., 2023b)	124 subjects	Event, Segment or epoch, Recording or night	Allocation unit NR; internal holdout	NR	Internal hold-out; partition unit unclear	PSG/AHI	NR	Reported metrics: Accuracy; Sensitivity; Specificity. See source study for task-specific values.	Allocation unit NR; No strict external test identified; Data/code: NR
(Li et al., 2023a)	104,689 patients	Event, Segment or epoch, Participant	Subject-dependent/overlap	five-fold cross-validation	Internal segment/subject-overlap evaluation	PSG/AHI; Expert or manual scoring	NR	Reported metrics: Accuracy; Sensitivity; Specificity; F1; Correlation/R2. See source study for task-specific values.	Subject overlap/dependence; No strict external test identified; Data/code: NR
(Lin et al., 2023)	UCDDB; Sleep-EDF; 104 patients; 70 subjects; 34 patients; 77 patients	Segment or epoch, Recording or night	Segment/epoch split; subject separation NR; internal holdout	Leave-One-Out; LOOCV	Internal segment/subject-overlap evaluation	Expert or manual scoring	NR	Reported metrics: Accuracy; Precision; Recall. See source study for task-specific values.	Participant separation not established; No strict external test identified; Data/code: NR
(Liu et al., 2023)	PhysioNet Apnea-ECG; cohort size NR	Segment or epoch, Recording or night	Segment/epoch split; subject separation NR; internal holdout	NR	Internal segment/subject-overlap evaluation	PSG/AHI; Expert or manual scoring	CNN; LSTM; Transformer	Reported metrics: Accuracy; Sensitivity; Specificity; Precision. See source study for task-specific values.	Participant separation not established; No strict external test identified; Code available
(Midelet et al., 2023)	Sleep Heart Health Study (SHHS); 23 patients	Event, Segment or epoch	Allocation unit NR	NR	Validation strategy not clearly reported or not applicable	NR	NR	Performance values NR in the extracted methodological description.	Allocation unit NR; No strict external test identified; Data/code: NR
(Moradhasel et al., 2023)	100 patients	Event, Segment or epoch, Participant	Allocation unit NR; internal holdout	NR	Internal hold-out; partition unit unclear	PSG/AHI	NR	Reported metrics: Accuracy; Specificity; Precision; Recall; F1; AUC/AUROC. See source study for task-specific values.	Allocation unit NR; No strict external test identified; Data/code: NR
(Seo et al., 2023)	PhysioNet Apnea-ECG; 216 patients; 105 patients; 111 patients	Event, Segment or epoch, Participant	Allocation unit NR; internal holdout	NR	Internal hold-out; partition unit unclear	Expert or manual scoring	CNN; LSTM	Reported metrics: Accuracy; Sensitivity; Specificity; AUC/AUROC; MAE; RMSE. See source study for task-specific values.	Allocation unit NR; No strict external test identified; Code available
(Shi et al., 2023)	MASS; 1,656 subjects	Event, Participant	Allocation unit NR; internal holdout	10-fold cross-validation	Internal hold-out; partition unit unclear	PSG/AHI	Logistic regression	Reported metrics: Accuracy; Sensitivity; Specificity; AUC/AUROC. See source study for task-specific values.	Allocation unit NR; No strict external test identified; Code available
(Srivastava et al., 2023)	PhysioNet Apnea-ECG; 35 Recordings; 28 Recordings; 7 Recordings	Event, Segment or epoch, Recording or night	Segment/epoch split; subject separation NR; internal holdout	NR	Internal segment/subject-overlap evaluation	PSG/AHI	CNN; LSTM	Reported metrics: Accuracy; Sensitivity; Specificity; F1. See source study for task-specific values.	Participant separation not established; No strict external test identified; Public data source reported

Continued on next page

Table S4 continued

Study	Dataset/cohort	Unit	Split/partition	CV	Validation	Reference standard	Comparator	Principal reported performance	Main methodological limitation; data/code
(Tyagi and Agrawal, 2023)	PhysioNet Apnea-ECG; Cohort size NR	Event, Segment or epoch, Recording or night	Allocation unit NR	NR	Validation strategy not clearly reported or not applicable	NR	NR	The proposed method shows significant improvements in performance when compared to other SLA detection methods for per-segment detection and achieved the highest accuracy of 89.11%, with specificity, sensitivity, and F1-score of 92.28%, 83.89%, and 0.913, respectively.	Allocation unit NR; No strict external test identified; Data/code: NR
(Ullah et al., 2023)	PhysioNet Apnea-ECG; 70 recordings; 35 subjects	Segment or epoch, Participant	Subject or recording separated; internal holdout	10-fold cross-validation	Internal subject-separated hold-out	PSG/AHI; Expert or manual scoring	LSTM; Neural network	Reported metrics: Accuracy; F1. See source study for task-specific values.	No strict external test identified; Data/code: NR
(Garcia-Vicente et al., 2023)	21 recordings; 1,610 recordings	Event, Segment or epoch, Recording or night	Segment/epoch split; subject separation NR; internal holdout	NR	Internal segment/subject-overlap evaluation	Clinical/AHI-derived labels	CNN	Reported metrics: Kappa. See source study for task-specific values.	Participant separation not established; No strict external test identified; Data/code: NR
(Wei et al., 2023)	PhysioNet Apnea-ECG; 110 recordings; 10 recordings; 100 recordings	Segment or epoch, Recording or night, Participant	Segment/epoch split; subject separation NR; internal holdout	5-fold cross validation	Internal segment/subject-overlap evaluation	Expert or manual scoring	NR	Our method achieved the result of 82.0% average ac curacy, 74.4% average sensitivity and 85.1% average specificity for per-segment SA detection, and reached 93.6% average accuracy for per-recording SA detection after 5-fold cross validation.	Participant separation not established; No strict external test identified; Data/code: NR
(Xie et al., 2023)	172 subjects	Event, Segment or epoch, Recording or night	Allocation unit NR	cross-validation	Cross-validation; partition unit unclear	PSG/AHI	NR	Reported metrics: Accuracy; MAE; Correlation/R2; Bland-Altman agreement. See source study for task-specific values.	Allocation unit NR; No strict external test identified; Data not publicly shareable; Public data source reported
(Xu et al., 2023)	196 participants; 168 patients; 39 patients	Participant	Allocation unit NR; internal holdout	NR	Internal hold-out; partition unit unclear	PSG/AHI	NR	Reported metrics: Sensitivity; Specificity; AUC/AUROC. See source study for task-specific values.	Allocation unit NR; No strict external test identified; Data on request
(Ylmaz et al., 2023)	MASS; 32 subjects; 8 subjects; 40 subjects	Segment or epoch, Recording or night, Participant	Subject-dependent/overlap	5-fold cross validation	Internal segment/subject-overlap evaluation	PSG/AHI	NR	Reported metrics: Accuracy; Sensitivity; Specificity; Precision; Recall; F1. See source study for task-specific values.	Subject overlap/dependence; No strict external test identified; Data/code: NR

Continued on next page

Table S4 continued

Study	Dataset/cohort	Unit	Split/partition	CV	Validation	Reference standard	Comparator	Principal reported performance	Main methodological limitation; data/code
(Zhang et al., 2023b)	27 patients	Event, Segment or epoch	Segment/epoch split; subject separation NR; internal holdout	NR	Internal segment/subject-overlap evaluation	PSG/AHI; Expert or manual scoring	SVM; Random forest; KNN; Decision tree	Reported metrics: Accuracy; Sensitivity. See source study for task-specific values.	Participant separation not established; No strict external test identified; Data/code: NR
(Zhang et al., 2023a)	Cohort size NR	Recording or night, Participant	Allocation unit NR; internal holdout	NR	Internal hold-out; partition unit unclear	PSG/AHI	NR	Reported metrics: Accuracy. See source study for task-specific values.	Allocation unit NR; No strict external test identified; Data/code: NR
(Zhou et al., 2023)	Sleep Heart Health Study (SHHS); MASS; 91 subjects	Event, Segment or epoch, Participant	Allocation unit NR	NR	Validation strategy not clearly reported or not applicable	PSG/AHI	NR	Reported metrics: Accuracy; Sensitivity; Specificity. See source study for task-specific values.	Allocation unit NR; No strict external test identified; Data/code: NR
(Cai et al., 2024)	1500 participants	Event, Segment or epoch, Participant	Allocation unit NR; internal holdout	NR	Internal hold-out; partition unit unclear	Expert or manual scoring	NR	Reported metrics: Precision; Recall. See source study for task-specific values.	Allocation unit NR; No strict external test identified; Code available
(Chen et al., 2024)	92 subjects; 29 subjects; 39 subjects	Event, Segment or epoch, Recording or night	Segment/epoch split; subject separation NR; internal holdout	cross-validation	Internal segment/subject-overlap evaluation	PSG/AHI; Expert or manual scoring	Logistic regression; SVM	Per-segment sleep-apnea detection accuracy was 70.17%.	Participant separation not established; No strict external test identified; Data/code: NR
(Choksatchawathi et al., 2024)	MESA Sleep; Cohort size NR	Event, Segment or epoch, Participant	Segment/epoch split; subject separation NR; internal holdout	cross-validation	Internal segment/subject-overlap evaluation	Expert or manual scoring	NR	Reported metrics: Accuracy; Sensitivity; F1; AUC/AUROC. See source study for task-specific values.	Participant separation not established; No strict external test identified; Code available
(Chu et al., 2024)	25 subjects; 12 patients	Event, Segment or epoch, Participant	Segment/epoch split; subject separation NR	ten-fold cross-validation	Internal segment/subject-overlap evaluation	Expert or manual scoring	NR	Reported metrics: Accuracy; Specificity. See source study for task-specific values.	Participant separation not established; No strict external test identified; Data/code: NR
(Jimenez-Garcia et al., 2024)	n = 1,638; n = 974	Event, Segment or epoch, Participant	Segment/epoch split; subject separation NR; internal holdout	NR	Independent clinical-database external test	Clinical/AHI-derived labels	CNN	Reported metrics: Accuracy; Sensitivity; Specificity. See source study for task-specific values.	Participant separation not established; Data not publicly shareable

Continued on next page

Table S4 continued

Study	Dataset/cohort	Unit	Split/partition	CV	Validation	Reference standard	Comparator	Principal reported performance	Main methodological limitation; data/code
(Ge et al., 2024)	MASS; 2,166 patients; 102 patients	Segment or epoch, Participant	Allocation unit NR; internal holdout	cross validation	Internal hold-out; partition unit unclear	PSG/AHI	NR	We compared the accuracy, Precision, Recall rate, F1-score and AUC indexes of the model, and finally obtained that MLP was the optimal model with an accuracy of 85.80%, Precision of 0.89, Recall of 0.75, F1-score of 0.82, and AUC of 0.938.	Allocation unit NR; No strict external test identified; Data on request
(Gupta et al., 2024)	PhysioNet Apnea-ECG; cohort size NR	Event, Segment or epoch, Participant	Allocation unit NR; internal holdout	NR	Internal hold-out; partition unit unclear	PSG/AHI	NR	Reported metrics: Accuracy. See source study for task-specific values.	Allocation unit NR; No strict external test identified; Data/code: NR
(Huang et al., 2024)	892 patients; 695 patients; 197 patients	Event, Participant	Allocation unit NR; internal holdout	NR	Site-separated testing cohort	PSG/AHI	NR	Site-test logistic regression achieved AUC 0.732, F1 0.788, and accuracy 0.701.	Allocation unit NR; Data/code: NR
(Ji et al., 2024)	MESA Sleep; 25 patients; 5 patients	Event, Segment or epoch, Participant	Allocation unit NR; internal holdout	cross validation; cross-validation	Internal hold-out; partition unit unclear	PSG/AHI; Expert or manual scoring	NR	Reported metrics: Precision; Recall; F1; MAE. See source study for task-specific values.	Allocation unit NR; No strict external test identified; Data not publicly shareable
(Jiao et al., 2024)	PhysioNet Apnea-ECG; 16 recordings; 2 recordings	Event, Segment or epoch, Recording or night	Subject or recording separated; internal holdout	NR	Internal subject-separated hold-out	PSG/AHI	CNN; LSTM	Reported metrics: Accuracy; Sensitivity; Precision; F1. See source study for task-specific values.	No strict external test identified; Data/code: NR
(Jirakittayakorn et al., 2024)	Sleep Heart Health Study (SHHS); MESA Sleep; MrOS Sleep; UCDDB; 6,814 participants; 2,056 PSG	Event, Segment or epoch, Participant	Segment/epoch split; subject separation NR; internal holdout	NR	Cross-dataset external validation	PSG/AHI	NR	External validation with the UCDDB dataset demonstrated 84.00% accuracy with 0.7743 Kappa, a macro-averaged F1-score of 86.69%, and an R-square of 0.7755, showing the model's robustness.	Participant separation not established; Data/code: NR
(Khan et al., 2024)	Sleep Heart Health Study (SHHS); National Sleep Research Resource; 2,434 patients	Segment or epoch, Recording or night, Participant	Allocation unit NR	NR	Validation strategy not clearly reported or not applicable	PSG/AHI; Expert or manual scoring	SVM; Random forest; Gradient boosting; KNN	Reported metrics: Accuracy; Precision; Recall; F1; AUC/AUROC. See source study for task-specific values.	Allocation unit NR; No strict external test identified; Data/code: NR
(Kim et al., 2024)	48 participants	Event, Segment or epoch, Participant	Allocation unit NR	10-fold cross validation	Cross-validation; partition unit unclear	PSG/AHI; Expert or manual scoring	NR	Reported metrics: Correlation/R2. See source study for task-specific values.	Allocation unit NR; No strict external test identified; Data on request

Continued on next page

Table S4 continued

Study	Dataset/cohort	Unit	Split/partition	CV	Validation	Reference standard	Comparator	Principal reported performance	Main methodological limitation; data/code
(Koda et al., 2024)	Cohort size NR	Event, Segment or epoch, Participant	Allocation unit NR	NR	Validation strategy not clearly reported or not applicable	Clinical/AHI-derived labels	NR	Reported metrics: Accuracy. See source study for task-specific values.	Allocation unit NR; No strict external test identified; Data/code: NR
(Leger and Elbaz, 2024)	MASS; N=67; N=72; N=139	Event, Segment or epoch, Participant	Subject or recording separated; internal holdout	NR	Internal subject-separated hold-out	PSG/AHI; Expert or manual scoring	Neural network	Reported metrics: Correlation/R2. See source study for task-specific values.	No strict external test identified; Data/code: NR
(Liu et al., 2024c)	N=71; 32 patients	Event	Allocation unit NR; internal holdout	leave-one-out	Internal hold-out; partition unit unclear	PSG/AHI	SVM	Reported metrics: Accuracy; Sensitivity; Specificity; Recall; AUC/AUROC. See source study for task-specific values.	Allocation unit NR; No strict external test identified; Data/code: NR
(Liu et al., 2024b)	367 participants (256 training, 111 validation)	Participant	Allocation unit NR; internal holdout	NR	Internal hold-out; partition unit unclear	PSG/AHI	NR	Reported metrics: Accuracy; Sensitivity; Specificity; AUC/AUROC. See source study for task-specific values.	Allocation unit NR; No strict external test identified; Data/code: NR
(Liu et al., 2024a)	PhysioNet Apnea-ECG; cohort size NR	Event, Segment or epoch, Recording or night	Segment/epoch split; subject separation NR; internal holdout	NR	Internal segment/subject-overlap evaluation	PSG/AHI	NR	Reported metrics: AUC/AUROC. See source study for task-specific values.	Participant separation not established; No strict external test identified; Data not publicly shareable
(Mandeville et al., 2024)	Cohort size NR	Event, Segment or epoch, Participant	Subject or recording separated; internal holdout	NR	Internal subject-separated hold-out	PSG/AHI	NR	Reported metrics: Accuracy; Sensitivity; Specificity. See source study for task-specific values.	No strict external test identified; Data/code: NR
(Nassehi et al., 2024)	25 participants; 15 Participants	Segment or epoch, Recording or night, Participant	Allocation unit NR; internal holdout	5-fold cross validation	Internal hold-out; partition unit unclear	PSG/AHI; Expert or manual scoring	SVM; KNN	Reported metrics: Accuracy; Sensitivity; Specificity; AUC/AUROC. See source study for task-specific values.	Allocation unit NR; No strict external test identified; Data on request
(Nguyen et al., 2024)	PhysioNet Apnea-ECG; UCDDB; 70 participants	Event, Segment or epoch, Recording or night	Subject or recording separated; internal holdout	NR	Internal subject-separated hold-out	Expert or manual scoring	CNN	Reported metrics: Accuracy; Sensitivity. See source study for task-specific values.	No strict external test identified; Code available
(Olsen et al., 2024)	MESA Sleep; Cohort size NR	Event, Segment or epoch, Recording or night	Segment/epoch split; subject separation NR; internal holdout	NR	External wearable-cohort validation	Clinical/AHI-derived labels	NR	Reported metrics: F1; Correlation/R2. See source study for task-specific values.	Participant separation not established; Code available

Continued on next page

Table S4 continued

Study	Dataset/cohort	Unit	Split/partition	CV	Validation	Reference standard	Comparator	Principal reported performance	Main methodological limitation; data/code
(Parbat and Chakraborty, 2024)	PhysioNet Apnea-ECG; Cohort size NR	Event, Segment or epoch	Allocation unit NR; internal holdout	NR	Internal hold-out; partition unit unclear	PSG/AHI	NR	Reported metrics: Accuracy; Sensitivity; Specificity; AUC/AUROC. See source study for task-specific values.	Allocation unit NR; No strict external test identified; Data/code: NR
(Pataka et al., 2024)	N = 2791; N = 1678; N = 1181; N = 1638	Event	Allocation unit NR	NR	Validation strategy not clearly reported or not applicable	PSG/AHI	NR	Performance values NR in the extracted methodological description.	Allocation unit NR; No strict external test identified; Data/code: NR
(Peng et al., 2024)	450 subjects	Event, Segment or epoch, Recording or night	Segment/epoch split; subject separation NR; internal holdout	5-fold cross-validation	Internal hold-out; partition unit unclear	Clinical/AHI-derived labels	CNN	Reported metrics: Accuracy; Sensitivity; Specificity; Precision; F1. See source study for task-specific values.	Participant separation not established; No strict external test identified; Data on request
(Retamales et al., 2024)	Sleep Heart Health Study (SHHS); MESA Sleep; MrOS Sleep; MASS; 14,370 recordings	Event, Segment or epoch, Participant	Segment/epoch split; subject separation NR; internal holdout	NR	Cross-dataset out-of-domain testing	PSG/AHI	NR	Abdominal-movement plus SpO_2 event detection achieved AUROC 0.87 and AUPRC 0.90; AHI-severity classification had approximately 0.72 F1.	Participant separation not established; Public data source reported
(Su et al., 2024)	MASS; n=1845; n=218; n=420; n=1207; n=300; n=360; n=12; n=36; n=52; n=747	Participant, Image or region	Allocation unit NR; internal holdout	NR	Independent-institution external validation	PSG/AHI	NR	Overall AUC was 0.812 in development and 0.763 externally; age-specific external AUCs were 0.714 and 0.846.	Allocation unit NR; Data on request
(Talukder et al., 2024)	Sleep Heart Health Study (SHHS); National Sleep Research Resource; N = 7075; N = 2261; N = 2260; N = 302; N = 1534; 20,802 studies	Event, Participant	Segment/epoch split; subject separation NR; internal holdout	five-fold cross validation	External validation in two independent datasets	PSG/AHI; Expert or manual scoring	XGBoost	AUROC was 0.741 internally, 0.697 in SHHS, and 0.819 in CFS; internal accuracy was 0.660.	Participant separation not established; Data/code: NR
(Thompson et al., 2024)	20 subject; 5 subject; 10 subject	Event, Segment or epoch	Allocation unit NR; internal holdout	NR	Internal hold-out; partition unit unclear	HSAT or polygraphy	SVM	Reported metrics: Accuracy; Sensitivity; Specificity; Precision; Recall. See source study for task-specific values.	Allocation unit NR; No strict external test identified; Data/code: NR

Continued on next page

Table S4 continued

Study	Dataset/cohort	Unit	Split/partition	CV	Validation	Reference standard	Comparator	Principal reported performance	Main methodological limitation; data/code
(Tyagi and Agrawal, 2024)	PhysioNet Apnea-ECG; 23 recordings; 12 recordings	Event, Segment or epoch, Recording or night	Allocation unit NR; internal holdout	10-fold cross-validation	Internal hold-out; partition unit unclear	NR	NR	The proposed method demonstrated substantial improvements compared to other SLA detection techniques, achieving a significant accuracy of 94.63 % for per-segment detection, along with specificity, sensitivity, F1-score and AUC values of 96.21 %, 92.04 %, 0.9285, and 0.9851 respectively.	Allocation unit NR; No strict external test identified; Code available
(Wang et al., 2024)	PhysioNet Apnea-ECG; 70 recordings (35 training, 35 testing)	Event, Segment or epoch, Recording or night	Allocation unit NR; internal holdout	NR	Cross-database transfer testing	Expert or manual scoring	CNN; LSTM	Reported metrics: Accuracy; Sensitivity; F1; RMSE. See source study for task-specific values.	Allocation unit NR; Data/code: NR
(Xie et al., 2024)	MASS; 198 patients	Event, Segment or epoch, Participant	Subject-dependent/overlap; internal holdout	cross-validation	Internal segment/subject-overlap evaluation	PSG/AHI; Expert or manual scoring	NR	Reported metrics: Sensitivity; Specificity; F1; Kappa; Correlation/R2. See source study for task-specific values.	Subject overlap/dependence; No strict external test identified; Data on request
(Yook et al., 2024)	Sleep Heart Health Study (SHHS); n = 179; n = 14,555	Event, Segment or epoch, Participant	Allocation unit NR; internal holdout	5-fold cross-validation	External public-dataset validation	PSG/AHI; Expert or manual scoring	Logistic regression; SVM; Random forest; KNN	Using RF and SpO2 feature sets, our model achieved an accuracy of 94 % in detecting apnea/hypopnea events.	Allocation unit NR; Data/code: NR
(Zhang et al., 2024)	PhysioNet Apnea-ECG; MASS; 798 patients (558 training, 240 testing)	Event, Participant	Allocation unit NR; internal holdout	10-fold cross validation	Internal hold-out; partition unit unclear	PSG/AHI; Expert or manual scoring	Logistic regression; Random forest	Reported metrics: Accuracy; Sensitivity; F1; AUC/AUROC. See source study for task-specific values.	Allocation unit NR; No strict external test identified; Data/code: NR
(Zhao et al., 2024c)	PhysioNet Apnea-ECG; Cohort size NR	Segment or epoch, Recording or night, Participant	Segment/epoch split; subject separation NR; internal holdout	NR	Internal segment/subject-overlap evaluation	PSG/AHI	NR	Reported metrics: Accuracy; Sensitivity; Specificity; F1. See source study for task-specific values.	Participant separation not established; No strict external test identified; Data/code: NR
(Zhao et al., 2024a)	n=307; n=25; n=178; n=18; n=8; 1,544 patients; 536 patients; 1,008 patients	Participant	Allocation unit NR; internal holdout	NR	Internal hold-out; partition unit unclear	PSG/AHI	NR	Reported metrics: Accuracy; Sensitivity; Specificity; AUC/AUROC. See source study for task-specific values.	Allocation unit NR; No strict external test identified; Data/code: NR
(Zhao et al., 2024b)	51 patients	Event, Segment or epoch, Recording or night	Segment/epoch split; subject separation NR; internal holdout	NR	Internal segment/subject-overlap evaluation	Clinical/AHI-derived labels	NR	Reported metrics: Accuracy; Sensitivity; Specificity; Correlation/R2. See source study for task-specific values.	Participant separation not established; No strict external test identified; Data/code: NR

Continued on next page

Table S4 continued

Study	Dataset/cohort	Unit	Split/partition	CV	Validation	Reference standard	Comparator	Principal reported performance	Main methodological limitation; data/code
(Zhou et al., 2024)	MASS; 357 participants; 350 participants	Event, Segment or epoch, Recording or night	Allocation unit NR	NR	Validation strategy not clearly reported or not applicable	PSG/AHI	NR	Reported metrics: Accuracy; Sensitivity; Specificity; AUC/AUROC; Kappa; Correlation/R2. See source study for task-specific values.	Allocation unit NR; No strict external test identified; Data/code: NR
(Zou and Liu, 2024)	PhysioNet Apnea-ECG; cohort size NR	Event, Segment or epoch, Recording or night	Subject or recording separated; internal holdout	cross-validation	Internal subject-separated hold-out	Expert or manual scoring	NR	Reported metrics: Accuracy; Sensitivity; Precision; F1; AUC/AUROC; MAE. See source study for task-specific values.	No strict external test identified; Data/code: NR
(Attar, 2025)	PhysioNet Apnea-ECG; 18 participants	Segment or epoch, Participant	Allocation unit NR; internal holdout	NR	Internal hold-out; partition unit unclear	PSG/AHI	NR	Reported metrics: Accuracy; Sensitivity; Specificity; Precision; Recall; F1. See source study for task-specific values.	Allocation unit NR; No strict external test identified; Data/code: NR
(Barroso-García et al., 2025)	Cohort size NR	Event, Segment or epoch, Participant	Subject or recording separated; internal holdout	NR	Independent multi-dataset testing	PSG/AHI; Expert or manual scoring	NR	In the test group, actual versus estimated AHI had ICC 0.69–0.87; four-class Cohen's kappa was 0.37–0.43.	Data/code: NR
(Bhongade et al., 2025)	70 recordings; 35 recordings	Segment or epoch	Segment/epoch split; subject separation NR; internal holdout	cross-validation	Internal segment/subject-overlap evaluation	PSG/AHI	SVM	Reported metrics: Accuracy; Sensitivity; Specificity; Precision; F1; Kappa. See source study for task-specific values.	Participant separation not established; No strict external test identified; Data/code: NR
(Cai et al., 2025)	PhysioNet Apnea-ECG; UCDDB; St. Vincent's University Hospital dataset; 32 subjects; 4 recordings; 70 recordings; 35 recordings	Segment or epoch, Recording or night, Participant	Subject-dependent/overlap; internal holdout	5-fold cross-validation	Internal segment/subject-overlap evaluation	PSG/AHI; Expert or manual scoring	NR	Reported metrics: Sensitivity. See source study for task-specific values.	Subject overlap/dependence; No strict external test identified; Data/code: NR
(Chen et al., 2025)	106 patients; 48 patients; 58 patients	Segment or epoch, Participant, Image or region	Allocation unit NR	cross-validation	Cross-validation; partition unit unclear	PSG/AHI	Logistic regression; SVM; Random forest; Gradient boosting	Reported metrics: Accuracy; F1; AUC/AUROC. See source study for task-specific values.	Allocation unit NR; No strict external test identified; Data/code: NR
(Choudhury et al., 2025)	PhysioNet Apnea-ECG; UCDDB; MIT-BIH; 16 subjects	Segment or epoch, Participant	Segment/epoch split; subject separation NR; internal holdout	5-fold cross validation	Internal segment/subject-overlap evaluation	NR	NR	Reported metrics: Accuracy; F1. See source study for task-specific values.	Participant separation not established; No strict external test identified; Data/code: NR

Continued on next page

Table S4 continued

Study	Dataset/cohort	Unit	Split/partition	CV	Validation	Reference standard	Comparator	Principal reported performance	Main methodological limitation; data/code
(Chu et al., 2025)	UCDDB; 39 subjects	Event, Segment or epoch, Participant	Segment/epoch split; subject separation NR; internal holdout	cross-validation	Internal segment/subject-overlap evaluation	PSG/AHI; Expert or manual scoring	NR	Reported metrics: Accuracy; Sensitivity; Precision; F1; Correlation/R2. See source study for task-specific values.	Participant separation not established; No strict external test identified; Data not publicly shareable
(Trang et al., 2025)	201 participants	Event, Segment or epoch	Allocation unit NR; internal holdout	NR	Internal hold-out; partition unit unclear	PSG/AHI; Expert or manual scoring	NR	Reported metrics: Accuracy; Sensitivity; Specificity; F1. See source study for task-specific values.	Allocation unit NR; No strict external test identified; Data/code: NR
(Dong et al., 2025)	PhysioNet Apnea-ECG; MIT-BIH; Cohort size NR	Segment or epoch, Recording or night, Participant	Segment/epoch split; subject separation NR; internal holdout	leave-one-subject-out; 10-fold cross-validation	Internal segment/subject-overlap evaluation	PSG/AHI; Expert or manual scoring	CNN	The proposed method achieved an accuracy of 94.08%, a macro F1 score of 93.02%, and a Cohen's kappa coefficient of 89.91% on the test set.	Participant separation not established; No strict external test identified; Code available
(Eris et al., 2025)	633 patients	Event, Participant	Allocation unit NR; internal holdout	NR	Internal hold-out; partition unit unclear	PSG/AHI; Expert or manual scoring	NR	Reported metrics: Accuracy; Sensitivity; Specificity; Precision; AUC/AUROC; Kappa. See source study for task-specific values.	Allocation unit NR; No strict external test identified; Data/code: NR
(Fang et al., 2025)	n = 40; n = 72; n = 313	Segment or epoch, Participant	Subject or recording separated; internal holdout	NR	Internal subject-separated hold-out	PSG/AHI	NR	Reported metrics: Sensitivity; Specificity. See source study for task-specific values.	No strict external test identified; Data/code: NR
(Frija et al., 2025)	50 patients; 46 patients; 44 patients	Event, Segment or epoch, Recording or night	Subject or recording separated	NR	Validation strategy not clearly reported or not applicable	PSG/AHI; Expert or manual scoring	NR	Reported metrics: Sensitivity; AUC/AUROC; Correlation/R2. See source study for task-specific values.	No strict external test identified; Data/code: NR
(Ganguly et al., 2025)	PhysioNet Apnea-ECG; 35 recordings	Segment or epoch, Participant	Allocation unit NR; internal holdout	NR	Internal hold-out; partition unit unclear	PSG/AHI	CNN	Reported metrics: Accuracy; Sensitivity; Specificity. See source study for task-specific values.	Allocation unit NR; No strict external test identified; Data/code: NR
(Garcia-Vicente et al., 2025)	n = 64; n = 981	Event, Segment or epoch, Participant	Subject or recording separated; internal holdout	NR	External validation in two independent pediatric cohorts	PSG/AHI; Expert or manual scoring	CNN	Reported metrics: Kappa. See source study for task-specific values.	Data/code: NR
(Gayen et al., 2025)	PhysioNet Apnea-ECG; 70 recordings (35 training, 35 testing)	Segment or epoch	Segment/epoch split; subject separation NR; internal holdout	NR	Internal segment/subject-overlap evaluation	Expert or manual scoring	SVM	Reported metrics: Accuracy. See source study for task-specific values.	Participant separation not established; No strict external test identified; Data/code: NR

Continued on next page

Table S4 continued

Study	Dataset/cohort	Unit	Split/partition	CV	Validation	Reference standard	Comparator	Principal reported performance	Main methodological limitation; data/code
(Gu and Fan, 2025)	Sleep-EDF; MASS; 15 participants; 32 participants	Event, Segment or epoch, Participant	Subject or recording separated; internal holdout	NR	Internal subject-separated hold-out	PSG/AHI	NR	Reported metrics: Accuracy; Recall; F1; AUC/AUROC. See source study for task-specific values.	No strict external test identified; Data/code: NR
(Hayano et al., 2025)	MASS; 41 subjects; 20-subject reported subset	Event, Segment or epoch, Recording or night	Allocation unit NR; internal holdout	NR	Internal hold-out; partition unit unclear	Expert or manual scoring	NR	Reported metrics: Accuracy; Sensitivity; Specificity; AUC/AUROC; Correlation/R2. See source study for task-specific values.	Allocation unit NR; No strict external test identified; Data on request
(Hossan et al., 2025)	PhysioNet Apnea-ECG; 70 recordings (35 training, 35 testing)	Segment or epoch, Recording or night	Allocation unit NR; internal holdout	NR	Internal hold-out; partition unit unclear	Expert or manual scoring	NR	Reported metrics: Accuracy; Sensitivity. See source study for task-specific values.	Allocation unit NR; No strict external test identified; Data/code: NR
(Hou et al., 2025b)	PhysioNet Apnea-ECG; Cohort size NR	Event, Segment or epoch, Recording or night	Subject or recording separated; internal holdout	NR	Internal subject-separated hold-out	Expert or manual scoring	CNN; LSTM; Transformer	Reported metrics: F1. See source study for task-specific values.	No strict external test identified; Data/code: NR
(Hou et al., 2025a)	PhysioNet Apnea-ECG; 70 recordings (35 training, 35 testing)	Segment or epoch, Participant	Allocation unit NR; internal holdout	5-fold cross-validation	Internal hold-out; partition unit unclear	PSG/AHI; Expert or manual scoring	Random forest; KNN	Reported metrics: Accuracy. See source study for task-specific values.	Allocation unit NR; No strict external test identified; Data/code: NR
(Hu et al., 2025)	PhysioNet Apnea-ECG; MESA Sleep; MASS; 5,793 subjects; 2,651 subjects	Event, Segment or epoch	Subject or recording separated; internal holdout	Five-fold subject-independent evaluation on target cohorts	Cross-database generalization	Clinical/AHI-derived labels	CNN; Transformer	Reported metrics: F1; AUC/AUROC; AUPRC. See source study for task-specific values.	Code available
(Jiang et al., 2025a)	36 children; 3 children; 30 children	Event, Segment or epoch, Participant	Allocation unit NR; internal holdout	NR	Internal hold-out; partition unit unclear	Expert or manual scoring	CNN	Reported metrics: Accuracy; AUC/AUROC; Kappa. See source study for task-specific values.	Allocation unit NR; No strict external test identified; Data/code: NR
(Jiang et al., 2025b)	PhysioNet Apnea-ECG; cohort size NR	Event, Segment or epoch, Participant	Allocation unit NR; internal holdout	5-fold cross-validation	Internal hold-out; partition unit unclear	PSG/AHI	NR	Reported metrics: Accuracy; Sensitivity; Specificity; F1. See source study for task-specific values.	Allocation unit NR; No strict external test identified; Data/code: NR
(Tirachaimongkol et al., 2025)	MASS; 40 participants; 35 participants	Event, Segment or epoch, Participant	Allocation unit NR; internal holdout	NR	Internal hold-out; partition unit unclear	PSG/AHI; Expert or manual scoring	NR	Reported metrics: Accuracy; Sensitivity; Specificity; Correlation/R2. See source study for task-specific values.	Allocation unit NR; No strict external test identified; Data on request

Continued on next page

Table S4 continued

Study	Dataset/cohort	Unit	Split/partition	CV	Validation	Reference standard	Comparator	Principal reported performance	Main methodological limitation; data/code
(Khan et al., 2025)	PhysioNet Apnea-ECG; Sleep Heart Health Study (SHHS); Cohort size NR	Segment or epoch, Recording or night, Participant	Allocation unit NR; internal holdout	NR	Internal hold-out; partition unit unclear	PSG/AHI	SVM	Reported metrics: Accuracy; Precision; Recall; F1; AUC/AUROC. See source study for task-specific values.	Allocation unit NR; No strict external test identified; Data/code: NR
(Kim et al., 2025a)	MASS; n=14; n=3; n=10; 61 patients; 37 patients; 33 patients; 53 participants; 38 subjects	Event, Segment or epoch, Recording or night	Allocation unit NR	NR	Prospective clinical validation	PSG/AHI; Expert or manual scoring	NR	Against physician-scored PSG at AHI $\geq$15, AUC was 0.931 (95% CI 0.862–0.999), with 78.6% sensitivity and 100% specificity.	Allocation unit NR; Data/code: NR
(Kim et al., 2025c)	Sleep Heart Health Study (SHHS); 284 patients	Event, Segment or epoch, Participant	Subject or recording separated; internal holdout	10-fold cross-validation	Internal subject-separated hold-out	PSG/AHI	LSTM; Neural network	Reported metrics: Accuracy; F1. See source study for task-specific values.	No strict external test identified; Data/code: NR
(Kim et al., 2025b)	25 subjects; 4 subjects; 20 subjects	Segment or epoch, Participant	Segment/epoch split; subject separation NR; internal holdout	NR	Unseen external-dataset generalization test	PSG/AHI; Expert or manual scoring	CNN	Reported metrics: Accuracy; Sensitivity; Specificity. See source study for task-specific values.	Participant separation not established; Data not publicly shareable
(Lai et al., 2025)	233 patients	Segment or epoch, Participant, Image or region	Subject or recording separated	NR	Validation strategy not clearly reported or not applicable	PSG/AHI; Expert or manual scoring	NR	Reported metrics: Accuracy; Sensitivity; Specificity; AUC/AUROC; Correlation/R2. See source study for task-specific values.	No strict external test identified; Data/code: NR
(Lee et al., 2025a)	Sleep Heart Health Study (SHHS); Cohort size NR	Segment or epoch	Allocation unit NR; internal holdout	NR	Internal hold-out; partition unit unclear	PSG/AHI; Expert or manual scoring	NR	Reported metrics: Accuracy; F1; Kappa. See source study for task-specific values.	Allocation unit NR; No strict external test identified; Code available
(Lee et al., 2025b)	80 participants	Event, Segment or epoch, Participant	Allocation unit NR; internal holdout	NR	Internal hold-out; partition unit unclear	Expert or manual scoring	NR	Reported metrics: Accuracy; AUC/AUROC. See source study for task-specific values.	Allocation unit NR; No strict external test identified; Data/code: NR
(Li et al., 2025b)	60 patients	Segment or epoch, Participant	Allocation unit NR	LOOCV	Cross-validation; partition unit unclear	PSG/AHI	SVM	Reported metrics: Accuracy; AUC/AUROC. See source study for task-specific values.	Allocation unit NR; No strict external test identified; Data/code: NR
(Li et al., 2025a)	166 subjects	Event, Participant	Allocation unit NR; internal holdout	Ten-fold cross-validation	Internal hold-out; partition unit unclear	PSG/AHI	NR	Reported metrics: Accuracy; Sensitivity; Specificity; Precision; F1; AUC/AUROC. See source study for task-specific values.	Allocation unit NR; No strict external test identified; Data/code: NR

Continued on next page

Table S4 continued

Study	Dataset/cohort	Unit	Split/partition	CV	Validation	Reference standard	Comparator	Principal reported performance	Main methodological limitation; data/code
(Yamane et al., 2025)	PhysioNet Apnea-ECG; N = 256	Segment or epoch, Recording or night, Participant	Allocation unit NR; internal holdout	5-fold cross-validation	Internal hold-out; partition unit unclear	PSG/AHI; Expert or manual scoring	Logistic regression; SVM; Random forest; XGBoost	Reported metrics: Accuracy; Sensitivity; Specificity; Precision; F1; AUC/AUROC. See source study for task-specific values.	Allocation unit NR; No strict external test identified; Public data source reported
(Mosteiro-Añón et al., 2025)	n = 1242; n = 908; n = 933; n = 1202; n = 1100	Event, Segment or epoch, Participant	Allocation unit NR; internal holdout	NR	Internal hold-out; partition unit unclear	Expert or manual scoring	NR	Reported metrics: AUC/AUROC. See source study for task-specific values.	Allocation unit NR; No strict external test identified; Data not publicly shareable
(Pham and Moucek, 2025)	PhysioNet Apnea-ECG; UCDDB; St. Vincent's University Hospital dataset; 25 patients	Segment or epoch, Recording or night, Participant	Allocation unit NR; internal holdout	five-fold cross-validation	Additional-dataset retraining (not strict external)	PSG/AHI; Expert or manual scoring	SVM; CNN; LSTM; Transformer; Neural network	Our model achieved an accuracy of 91.6% for per-segment classification on the Physionet Apnea-ECG dataset using hold-out validation and the highest accuracy of 94.1% using five-fold cross-validation.	Allocation unit NR; No strict external test identified; Data/code: NR
(Qiu et al., 2025)	PhysioNet Apnea-ECG; 92 subjects	Event, Segment or epoch, Recording or night	Allocation unit NR; internal holdout	five-fold cross-validation	Internal hold-out; partition unit unclear	PSG/AHI	LSTM	Reported metrics: Accuracy; Sensitivity; Specificity; F1; AUC/AUROC; MAE. See source study for task-specific values.	Allocation unit NR; No strict external test identified; Code available
(Rehman et al., 2025)	ISRUC-Sleep; 12 subjects; 4 subjects	Event, Segment or epoch, Participant	Segment/epoch split; subject separation NR; internal holdout	ten-fold cross-validation	Internal segment/subject-overlap evaluation	PSG/AHI	SVM; Decision tree	Reported metrics: Accuracy; Sensitivity; Specificity; Precision; F1; AUC/AUROC. See source study for task-specific values.	Participant separation not established; No strict external test identified; Data not publicly shareable
(Rehman and Saleh, 2025)	ISRUC-Sleep; 100 subjects; 10-subject reported subset	Event, Segment or epoch, Participant	Segment/epoch split; subject separation NR; internal holdout	10-fold cross-validation	Internal segment/subject-overlap evaluation	PSG/AHI; Expert or manual scoring	NR	Reported metrics: Precision; F1; AUC/AUROC. See source study for task-specific values.	Participant separation not established; No strict external test identified; Data/code: NR
(Saha et al., 2025)	Sleep Heart Health Study (SHHS); 50 participants	Event, Segment or epoch	Segment/epoch split; subject separation NR; internal holdout	NR	Internal segment/subject-overlap evaluation	Clinical/AHI-derived labels	NR	Results showed high accuracy in differentiating central from obstructive respiratory events, with the combined CNN architecture achieving an overall accuracy of 83.66 % and a sensitivity and specificity above 83 %.	Participant separation not established; No strict external test identified; Data/code: NR

Continued on next page

Table S4 continued

Study	Dataset/cohort	Unit	Split/partition	CV	Validation	Reference standard	Comparator	Principal reported performance	Main methodological limitation; data/code
(Chao et al., 2025)	MASS; 20 participants; 40 participants	Event, Segment or epoch, Participant	Allocation unit NR; internal holdout	NR	Internal hold-out; partition unit unclear	PSG/AHI	NR	Reported metrics: Accuracy; Sensitivity; Specificity; Precision; Recall; F1. See source study for task-specific values.	Allocation unit NR; No strict external test identified; Data/code: NR
(Shen et al., 2025)	PhysioNet Apnea-ECG; Cohort size NR	Segment or epoch, Recording or night, Participant	Segment/epoch split; subject separation NR; internal holdout	10-fold cross-validation	Internal segment/subject-overlap evaluation	PSG/AHI; Expert or manual scoring	CNN	Reported metrics: Accuracy; Sensitivity; Specificity; F1. See source study for task-specific values.	Participant separation not established; No strict external test identified; Data/code: NR
(Sillaparaya et al., 2025)	7 patient	Event, Segment or epoch, Participant	Subject-dependent/overlap; internal holdout	NR	Internal segment/subject-overlap evaluation	PSG/AHI; Expert or manual scoring	NR	Reported metrics: Accuracy; Sensitivity; Specificity; Precision; F1. See source study for task-specific values.	Subject overlap/dependence; No strict external test identified; Data/code: NR
(Song et al., 2025)	305 subjects	Event, Segment or epoch, Recording or night	Subject-dependent/overlap; internal holdout	NR	Internal segment/subject-overlap evaluation	PSG/AHI; Expert or manual scoring	LSTM	Accuracy/F1 were 88.32%/86.28% with a subject-dependent split and 68.55%/60.29% with a subject-independent split.	Subject overlap/dependence; No strict external test identified; Data/code: NR
(Tan et al., 2025)	MASS; 1492 participants	Participant	Allocation unit NR; internal holdout	NR	Internal hold-out; partition unit unclear	PSG/AHI	NR	Reported metrics: Accuracy; Sensitivity; Specificity; AUC/AUROC. See source study for task-specific values.	Allocation unit NR; No strict external test identified; Data on request
(Ullah et al., 2025)	PhysioNet Apnea-ECG; Sleep Heart Health Study (SHHS); cohort size NR	Segment or epoch, Participant	Allocation unit NR; internal holdout	cross-validation	Cross-dataset generalization	Expert or manual scoring	CNN	Reported metrics: AUC/AUROC. See source study for task-specific values.	Allocation unit NR; Code available
(Wan et al., 2025)	Sleep Heart Health Study (SHHS); 329 SUBJECTS	Segment or epoch	Allocation unit NR; internal holdout	NR	Internal hold-out; partition unit unclear	Clinical/AHI-derived labels	NR	Reported metrics: Accuracy; Sensitivity; Precision; F1. See source study for task-specific values.	Allocation unit NR; No strict external test identified; Data/code: NR
(Wang et al., 2025)	Sleep Heart Health Study (SHHS); 6,441 recordings	Event, Segment or epoch, Participant	Segment/epoch split; subject separation NR; internal holdout	NR	Cross-dataset generalization	Expert or manual scoring	CNN; LSTM	Reported metrics: F1; AUC/AUROC. See source study for task-specific values.	Participant separation not established; Data/code: NR
(Werthen-Brabants et al., 2025)	N = 33	Event, Segment or epoch, Recording or night	Allocation unit NR; internal holdout	NR	Internal hold-out; partition unit unclear	Clinical/AHI-derived labels	CNN	Reported metrics: Accuracy; Sensitivity; Precision; AUC/AUROC. See source study for task-specific values.	Allocation unit NR; No strict external test identified; Data/code: NR

Continued on next page

Table S4 continued

Study	Dataset/cohort	Unit	Split/partition	CV	Validation	Reference standard	Comparator	Principal reported performance	Main methodological limitation; data/code
(Xue et al., 2025)	Cohort size NR	Segment or epoch, Participant	Allocation unit NR; internal holdout	NR	Internal hold-out; partition unit unclear	Expert or manual scoring	NR	Reported metrics: AUC/AUROC. See source study for task-specific values.	Allocation unit NR; No strict external test identified; Data/code: NR
(Zan et al., 2025)	Cohort size NR	Event, Segment or epoch, Recording or night	Subject or recording separated; internal holdout	NR	Internal subject-separated hold-out	Clinical/AHI-derived labels	NR	Reported metrics: Accuracy. See source study for task-specific values.	No strict external test identified; Data/code: NR
(Zhang et al., 2025b)	n = 26; 26 patients	Event, Segment or epoch, Participant	Subject or recording separated	leave-one-patient-out	Internal subject-separated hold-out	PSG/AHI; Expert or manual scoring	NR	Reported metrics: Accuracy; Sensitivity; Specificity; Correlation/R2. See source study for task-specific values.	No strict external test identified; Data/code: NR
(Zhang et al., 2025c)	PhysioNet Apnea-ECG; UCDDB; 510 patients; 8 recordings; 21 recordings	Event, Segment or epoch, Recording or night	Segment/epoch split; subject separation NR; internal holdout	cross-validation	Internal segment/subject-overlap evaluation	PSG/AHI; Expert or manual scoring	NR	Reported metrics: F1; AUC/AUROC. See source study for task-specific values.	Participant separation not established; No strict external test identified; Data/code: NR
(Zhang et al., 2025d)	MESA Sleep; UCDDB; St. Vincent's University Hospital dataset; 317 subjects	Event, Segment or epoch, Participant	Allocation unit NR; internal holdout	NR	Internal hold-out; partition unit unclear	PSG/AHI	NR	Reported metrics: Accuracy; Specificity; AUC/AUROC. See source study for task-specific values.	Allocation unit NR; No strict external test identified; Data/code: NR
(Zhang et al., 2025a)	n = 17; 31 patients; 26 participants	Event, Segment or epoch, Participant	Allocation unit NR; internal holdout	NR	Internal hold-out; partition unit unclear	PSG/AHI	NR	Reported metrics: Accuracy; Sensitivity; Specificity; AUC/AUROC; Kappa; Correlation/R2. See source study for task-specific values.	Allocation unit NR; No strict external test identified; Data/code: NR
(Zhao et al., 2025)	PhysioNet Apnea-ECG; 12 recordings	Segment or epoch, Recording or night, Participant	Segment/epoch split; subject separation NR; internal holdout	NR	Internal segment/subject-overlap evaluation	PSG/AHI; Expert or manual scoring	CNN; LSTM	Reported metrics: Accuracy; Sensitivity; Specificity; F1. See source study for task-specific values.	Participant separation not established; No strict external test identified; Data/code: NR
(Akter et al., 2026b)	PhysioNet Apnea-ECG; 70 recordings; 70 subjects; 35 subjects	Segment or epoch, Participant	Segment/epoch split; subject separation NR; internal holdout	5-fold cross-validation	Internal segment/subject-overlap evaluation	PSG/AHI	LSTM	Reported metrics: Accuracy. See source study for task-specific values.	Participant separation not established; No strict external test identified; Code available
(Akter et al., 2026a)	PhysioNet Apnea-ECG; 70 recordings	Event, Segment or epoch, Participant	Segment/epoch split; subject separation NR	Three-fold inter-patient cross-validation	Internal segment/subject-overlap evaluation	Expert or manual scoring	CNN	Reported metrics: Precision; Recall; F1. See source study for task-specific values.	Participant separation not established; No strict external test identified; Public data source reported

Continued on next page

Table S4 continued

Study	Dataset/cohort	Unit	Split/partition	CV	Validation	Reference standard	Comparator	Principal reported performance	Main methodological limitation; data/code
(Chi et al., 2026)	Sleep Heart Health Study (SHHS); cohort size NR	Event, Participant	Allocation unit NR; internal holdout	5-fold cross-validation	Internal hold-out; partition unit unclear	PSG/AHI	CNN	Reported metrics: F1. See source study for task-specific values.	Allocation unit NR; No strict external test identified; Code available
(Faridsoltani et al., 2026)	20 subjects; 19 subjects	Event, Segment or epoch, Recording or night	Subject or recording separated; internal holdout	leave-one-out; LOOCV	Internal subject-separated hold-out	Clinical/AHI-derived labels	CNN; LSTM; Transformer	LOOCV per-segment accuracy was 93.85% for two-class and 88.28% for three-class classification; corresponding F1 values were 90.6% and 82.62%.	No strict external test identified; Data/code: NR
(Gayen et al., 2026)	PhysioNet Apnea-ECG; MIT-BIH; Cohort size NR	Segment or epoch	Allocation unit NR; internal holdout	NR	Internal hold-out; partition unit unclear	PSG/AHI; Expert or manual scoring	NR	Reported metrics: F1. See source study for task-specific values.	Allocation unit NR; No strict external test identified; Public data source reported
(Han et al., 2026)	103 participants; 120 participants; 13 participants	Event, Segment or epoch, Recording or night	Subject or recording separated; internal holdout	cross-validation	Internal subject-separated hold-out	PSG/AHI	Neural network	Reported metrics: Accuracy; Sensitivity; Specificity; Correlation/R2. See source study for task-specific values.	No strict external test identified; Data/code: NR
(Hsu et al., 2026)	277 adults; 86 subjects	Event, Segment or epoch, Participant	Allocation unit NR	leave-one-out; LOOCV	Cross-validation; partition unit unclear	PSG/AHI	NR	Reported metrics: Precision; Recall; AUC/AUROC; AUPRC. See source study for task-specific values.	Allocation unit NR; No strict external test identified; Data/code: NR
(Hu et al., 2026)	128 subjects	Event, Segment or epoch, Participant	Subject or recording separated; internal holdout	LOSO	Internal subject-separated hold-out	Clinical/AHI-derived labels	LSTM	Reported metrics: Accuracy; Precision; Recall; F1. See source study for task-specific values.	No strict external test identified; Data/code: NR
(Kamal et al., 2026)	ISRUC-Sleep; MASS; Cohort size NR	Segment or epoch, Recording or night, Participant	Subject or recording separated; internal holdout	cross-validation	Internal subject-separated hold-out	PSG/AHI; Expert or manual scoring	NR	Reported metrics: Accuracy; Precision; Recall; F1. See source study for task-specific values.	No strict external test identified; Data/code: NR
(Kuhn et al., 2026)	1,485 recordings; reported participant subgroups: n=64, 33, 6, and 17	Event, Participant	Allocation unit NR	NR	Validation strategy not clearly reported or not applicable	PSG/AHI; Expert or manual scoring	NR	Reported metrics: Sensitivity. See source study for task-specific values.	Allocation unit NR; No strict external test identified; Data not publicly shareable; Public data source reported
(Kwon et al., 2026)	n = 6; n = 41; n = 68; n = 15; n = 48	Event, Segment or epoch, Participant	Subject or recording separated; internal holdout	NR	Internal subject-separated hold-out	PSG/AHI; Expert or manual scoring	CNN; Transformer	Reported metrics: Accuracy; Sensitivity; Specificity; Kappa. See source study for task-specific values.	No strict external test identified; Data/code: NR

Continued on next page

Table S4 continued

Study	Dataset/cohort	Unit	Split/partition	CV	Validation	Reference standard	Comparator	Principal reported performance	Main methodological limitation; data/code
(Li et al., 2026)	6,263 subjects; 9,748 patients; 7,634 subjects	Event, Segment or epoch, Participant	Subject-dependent/overlap; internal holdout	NR	Internal segment/subject-overlap evaluation	Clinical/AHI-derived labels	NR	Reported metrics: Accuracy; Sensitivity; F1; AUC/AUROC. See source study for task-specific values.	Subject overlap/dependence; No strict external test identified; Data/code: NR
(Liu et al., 2026c)	Sleep Heart Health Study (SHHS); MESA Sleep; 62 patients	Event, Segment or epoch, Participant	Subject or recording separated; internal holdout	cross-validation	Internal subject-separated hold-out	Clinical/AHI-derived labels	NR	Reported metrics: Accuracy; Precision; Recall; F1. See source study for task-specific values.	No strict external test identified; Data/code: NR
(Liu et al., 2026a)	100 children	Event, Segment or epoch, Participant	Allocation unit NR; internal holdout	NR	Internal hold-out; partition unit unclear	PSG/AHI; Expert or manual scoring	NR	Reported metrics: AUC/AUROC. See source study for task-specific values.	Allocation unit NR; No strict external test identified; Data/code: NR
(Liu et al., 2026b)	n = 201; n = 89; n = 67; n = 36; n = 734; n = 1,001; n = 1,263; n = 1,038	Participant	Allocation unit NR; internal holdout	10-fold cross-validation	Prospective clinical validation	Expert or manual scoring	XGBoost; STOP-BANG	Prospective moderate-to-severe OSA AUC was 0.91 (95% CI 0.88–0.94), 0.03 below the derivation result.	Allocation unit NR; Data/code: NR
(Ma et al., 2026)	61 patients; 29 patients	Event, Segment or epoch, Participant	Allocation unit NR; internal holdout	NR	Internal hold-out; partition unit unclear	PSG/AHI; Expert or manual scoring	NR	Reported metrics: Accuracy; Sensitivity; Specificity; AUC/AUROC. See source study for task-specific values.	Allocation unit NR; No strict external test identified; Data on request
(Niemi et al., 2026)	Sleep Heart Health Study (SHHS); n = 3206; n = 2476	Event, Segment or epoch, Participant	Segment/epoch split; subject separation NR	NR	Independent large-cohort replication of a previously evaluated method	PSG/AHI	NR	Reported metrics: AUC/AUROC. See source study for task-specific values.	Participant separation not established; Data/code: NR
(Qi et al., 2026)	PhysioNet Apnea-ECG; Sleep Heart Health Study (SHHS); ISRUC-Sleep; 6441 participants	Event, Segment or epoch	Subject or recording separated; internal holdout	cross-validation	Cross-dataset subject-independent validation	PSG/AHI; Expert or manual scoring	NR	Reported metrics: F1. See source study for task-specific values.	Public data source reported
(Rohrscheib et al., 2026)	n = 44; n = 139; 44 patients	Event, Participant	Allocation unit NR; internal holdout	NR	Internal hold-out; partition unit unclear	PSG/AHI; Expert or manual scoring	NR	Reported metrics: Correlation/R2; Bland-Altman agreement. See source study for task-specific values.	Allocation unit NR; No strict external test identified; Data/code: NR
(Strumpf et al., 2026)	MESA Sleep; 122 participants	Event, Segment or epoch, Participant	Subject or recording separated; internal holdout	10-fold cross validation	Internal subject-separated hold-out	PSG/AHI; Expert or manual scoring	SVM	Reported metrics: Accuracy; Sensitivity; Specificity; F1. See source study for task-specific values.	No strict external test identified; Data on request
(TaghiBeyglou et al., 2026)	MASS; 151 participants; 40 participants; 37 participants; 74 participants	Segment or epoch, Participant	Allocation unit NR; internal holdout	NR	Internal hold-out; partition unit unclear	PSG/AHI	NR	Reported metrics: F1; AUC/AUROC; MAE; Correlation/R2. See source study for task-specific values.	Allocation unit NR; No strict external test identified; Data/code: NR

Continued on next page

Table S4 continued

Study	Dataset/cohort	Unit	Split/partition	CV	Validation	Reference standard	Comparator	Principal reported performance	Main methodological limitation; data/code
(Tang et al., 2026)	PhysioNet Apnea-ECG; 32 subjects	Event, Segment or epoch, Participant	Allocation unit NR; internal holdout	Five-fold cross-validation	Internal hold-out; partition unit unclear	NR	NR	Reported metrics: Accuracy; Sensitivity; F1. See source study for task-specific values.	Allocation unit NR; No strict external test identified; Data/code: NR
(Tani et al., 2026)	100 participants	Event, Segment or epoch, Recording or night	Allocation unit NR	NR	Prospective clinical validation	PSG/AHI; Expert or manual scoring	Transformer	Reported metrics: Accuracy; Sensitivity; Specificity; Correlation/R2. See source study for task-specific values.	Allocation unit NR; Data/code: NR
(Thuptimdang et al., 2026)	45 subjects	Event, Segment or epoch, Participant	Subject or recording separated; internal holdout	5-fold cross-validation	Internal subject-separated hold-out	PSG/AHI; Expert or manual scoring	NR	Reported metrics: Accuracy; Sensitivity; Specificity; Precision; AUC/AUROC. See source study for task-specific values.	No strict external test identified; Data on request
(Tian et al., 2026)	MESA Sleep; Cohort size NR	Event, Segment or epoch, Recording or night	Segment/epoch split; subject separation NR; internal holdout	five-fold cross-validation	Internal segment/subject-overlap evaluation	PSG/AHI; Expert or manual scoring	CNN; LSTM; Transformer	Reported metrics: Accuracy; F1. See source study for task-specific values.	Participant separation not established; No strict external test identified; Data/code: NR
(Gutierrez-Tobal et al., 2026)	MASS; 464 children; 407 subjects	Event, Participant	Subject or recording separated	leave-one-out	Internal subject-separated hold-out	PSG/AHI	NR	Reported metrics: Sensitivity. See source study for task-specific values.	No strict external test identified; Data/code: NR
(Wang et al., 2026a)	MASS; 73 participants	Event, Segment or epoch, Participant	Subject or recording separated; internal holdout	NR	Internal subject-separated hold-out	Expert or manual scoring	CNN	Reported metrics: Accuracy; Precision; Recall; F1. See source study for task-specific values.	No strict external test identified; Code available
(Wang et al., 2026b)	Sleep Heart Health Study (SHHS); MESA Sleep; 6,400 adults; 5,793 recordings; 2,651 recordings; 5,519 recordings	Event, Segment or epoch, Participant	Subject-dependent/overlap; internal holdout	cross-validation	Cross-dataset transfer/fine-tuning (not strict external)	PSG/AHI; Expert or manual scoring	NR	Reported metrics: Sensitivity; MAE. See source study for task-specific values.	Subject overlap/dependence; No strict external test identified; Code available
(Wu et al., 2026)	114,541 patients	Event, Segment or epoch, Participant	Allocation unit NR; internal holdout	NR	Internal hold-out; partition unit unclear	Expert or manual scoring	LSTM	Reported metrics: MAE. See source study for task-specific values.	Allocation unit NR; No strict external test identified; Data/code: NR
(Yang et al., 2026)	162 patients; 157 subjects; 5 subjects	Event, Participant	Segment/epoch split; subject separation NR; internal holdout	cross-validation	Internal segment/subject-overlap evaluation	Clinical/AHI-derived labels	NR	Reported metrics: Sensitivity. See source study for task-specific values.	Participant separation not established; No strict external test identified; Data on request

Continued on next page

Table S4 continued

Study	Dataset/cohort	Unit	Split/partition	CV	Validation	Reference standard	Comparator	Principal reported performance	Main methodological limitation; data/code
(Ye et al., 2026)	PhysioNet Apnea-ECG; MIT-BIH; 70 recordings	Event, Segment or epoch, Participant	Allocation unit NR; internal holdout	NR	Internal hold-out; partition unit unclear	PSG/AHI; Expert or manual scoring	NR	Reported metrics: Accuracy; Precision; Recall; F1. See source study for task-specific values.	Allocation unit NR; No strict external test identified; Public data source reported
(Yoo et al., 2026)	MASS; n = 543; 879 patients; 20 patients	Segment or epoch, Participant, Image or region	Allocation unit NR; internal holdout	five-fold cross-validation	External validation with very small independent sample	PSG/AHI	NR	The trimodal model had internal AUC 0.859; three of four external participants were correctly classified.	Allocation unit NR; External $n = 4$; Data/code: NR
(Zhang et al., 2026a)	PhysioNet Apnea-ECG; UCDDB; cohort size NR	Event, Segment or epoch	Allocation unit NR; internal holdout	10-fold cross-validation	Cross-dataset robustness testing	Expert or manual scoring	NR	Reported metrics: Accuracy; Sensitivity; Specificity; AUC/AUROC. See source study for task-specific values.	Allocation unit NR; Data/code: NR
(Zhang et al., 2026b)	Sleep Heart Health Study (SHHS); National Sleep Research Resource; 800 PSG	Event, Segment or epoch, Participant	Subject or recording separated; internal holdout	NR	Internal subject-separated hold-out	PSG/AHI	NR	Reported metrics: Accuracy. See source study for task-specific values.	No strict external test identified; Data/code: NR
(Zhang et al., 2026c)	MESA Sleep; UCDDB; St. Vincent's University Hospital dataset; 317 subjects	Event, Segment or epoch, Recording or night	Segment/epoch split; subject separation NR; internal holdout	five-fold cross-validation; leave-one-out	Internal segment/subject-overlap evaluation	PSG/AHI	NR	Reported metrics: Sensitivity; Specificity. See source study for task-specific values.	Participant separation not established; No strict external test identified; Data/code: NR

Note: AI, artificial intelligence; AHI, apnea–hypopnea index; AUC/AUROC, area under the receiver-operating-characteristic curve; AUPRC, area under the precision–recall curve; CV, cross-validation; HSAT, home sleep apnea testing; NR, not reported or not identifiable unambiguously from the available article; OSA, obstructive sleep apnea; PSG, polysomnography; SDB, sleep-disordered breathing. Validation and limitation labels provide a structured descriptive appraisal and do not constitute a formal risk-of-bias rating. Cross-dataset fine-tuning and target-dataset retraining were not classified as strict external validation. Performance values retain the task definitions and thresholds of the source studies and should not be compared as if they arose from a common benchmark.

REFERENCES

Akter, M., Yousuf, M. A., Ahmed, S., Kaiser, M. S., and Uddin, M. Z. (2026a). Mvic-net: Multi-view interactive convolutional networks for ecg-based sleep apnea detection with explainable ai. *Knowledge-Based Systems* 340, 115660. doi:10.1016/j.knosys.2026.115660

Akter, S., Masud, M. A., Promi, M. S. I., Sultana, N., Ahmed, M., Rahman, M. M., et al. (2026b). Dream: A novel explainable neural network for detecting sleep apnea using single-lead ecg signals. *Biomedical Signal Processing and Control* 114, 109291. doi:10.1016/j.bspc.2025.109291

Almutairi, H. and Hassan, G. M. (2021). Classification of obstructive sleep apnoea from single-lead ecg signals using convolutional neural and long short term memory networks. *Biomedical Signal Processing and Control* 69, 102906. doi:10.1016/j.bspc.2021.102906

Aswath, S., Shanmuga Sundaram, V. R., and Mahdal, M. (2023). An adaptive sleep apnea detection model using multi cascaded atrous-based deep learning schemes with hybrid artificial humming bird pity beetle algorithm. *IEEE Access* 11, 113114–113133. doi:10.1109/ACCESS.2023.3319452

Attar, E. T. (2025). Detailed evaluation of sleep apnea using heart rate variability: a machine learning and statistical method using ecg data. *Frontiers in Neurology* 16, 1636983. doi:10.3389/fneur.2025.1636983

Bahr-Hamm, K., Abriani, A., Anwar, A. R., Ding, H., Muthuraman, M., and Gouveris, H. (2023). Using entropy of snoring, respiratory effort and electrocardiography signals during sleep for osa detection and severity classification. *Sleep Medicine* 111, 21–27. doi:10.1016/j.sleep.2023.09.005

Balci, M., Tasdemir, S., Ozmen, G., and Golcuk, A. (2022). Machine learning-based detection of sleep-disordered breathing type using time and time-frequency features. *Biomedical Signal Processing and Control* 73, 103402. doi:10.1016/j.bspc.2021.103402

Bark, B., Nam, B., and Kim, I. Y. (2023). SelANet: decision-assisting selective sleep apnea detection based on confidence score. *BMC Medical Informatics and Decision Making* 23, 190. doi:10.1186/s12911-023-02292-3

Barroso-García, V., Vaquerizo-Villar, F., Gutiérrez-Tobal, G. C., Dayyat, E., Gozal, D., Leppänen, T., et al. (2025). An explainable deep-learning approach to detect pediatric sleep apnea from single-channel airflow. *IEEE Journal of Translational Engineering in Health and Medicine* 13, 517–531. doi:10.1109/JTEHM.2025.3625388

Benedetti, D., Olcese, U., Bruno, S., Barsotti, M., Maestri Tassoni, M., Bonanni, E., et al. (2022). Obstructive sleep apnoea syndrome screening through wrist-worn smartbands: A machine-learning approach. *Nature and Science of Sleep* 14, 941–956. doi:10.2147/NSS.S352335

Bernardini, A., Brunello, A., Gigli, G. L., Montanari, A., and Saccomanno, N. (2021). Aiosa: An approach to the automatic identification of obstructive sleep apnea events based on deep learning. *Artificial Intelligence In Medicine* 118, 102133. doi:10.1016/j.artmed.2021.102133

Bhalerao, S. V. and Pachori, R. B. (2022). Sparse spectrum based swarm decomposition for robust nonstationary signal analysis with application to sleep apnea detection from eeg. *Biomedical Signal Processing and Control* 77, 103792. doi:10.1016/j.bspc.2022.103792

Bhongade, A., Gandhi, T. K., et al. (2025). Automatic identification of obstructive sleep apnea using multimodal features. *Biomedical Signal Processing and Control* 105, 107609

Blanchard, M., Feuilloy, M., Sabil, A., Gervès-Pinquié, C., Gagnadoux, F., and Girault, J.-M. (2022). A deep survival learning approach for cardiovascular risk estimation in patients with sleep apnea. *IEEE Access* 10, 133468–133478. doi:10.1109/ACCESS.2022.3231743

Bokov, P., Dudoignon, B., Boujemla, I., Dahan, J., Spruyt, K., and Delclaux, C. (2023). Development and validation of moderate to severe obstructive sleep apnea screening test (colton) in a pediatric population. *Sleep Medicine* 104, 11–17. doi:10.1016/j.sleep.2023.02.016

Bricout, A., Fontecave-Jallon, J., Pepin, J.-L., and Gumery, P.-Y. (2021). Accelerometry-derived respiratory index estimating apnea-hypopnea index for sleep apnea screening. *Computer Methods and Programs in Biomedicine* 207, 106209. doi:10.1016/j.cmpb.2021.106209

Cai, F., Siddiquee, M. M. R., Wu, T., Lubecke, V. M., and Boric-Lubecke, O. (2025). Tp-cl: A novel temporal proximity contrastive learning approach for obstructive sleep apnea detection using single-lead electrocardiograms. *Biomedical Signal Processing and Control* 100, 106993. doi:10.1016/j.bspc.2024.106993

Cai, J., Xiu, T., Song, Y., Fan, X., Wu, J., Tuohuti, A., et al. (2024). Deep learning-based quantification of adenoid hypertrophy and its correlation with apnea-hypopnea index in pediatric obstructive sleep apnea. *Nature and Science of Sleep* 16, 2243–2256. doi:10.2147/NSS.S492146

Chao, Y.-P., Chuang, H.-H., Lee, Z.-H., Huang, S.-Y., Zhan, W.-T., Shyu, L.-Y., et al. (2025). Distinguishing severe sleep apnea from habitual snoring using a neck-wearable piezoelectric sensor and deep learning: A pilot study. *Computers in Biology and Medicine* 190, 110070. doi:10.1016/j.compbiomed.2025.110070

Chen, J.-W., Liu, C.-M., Wang, C.-Y., Lin, C.-C., Qiu, K.-Y., Yeh, C.-Y., et al. (2023a). A deep neural network-based model for osa severity classification using unsegmented peripheral oxygen saturation signals. *Engineering Applications of Artificial Intelligence* 122, 106161. doi:10.1016/j.engappai.2023.106161

Chen, T., Zhang, J., Xu, Z., Redmond, S. J., Lovell, N. H., Liu, G., et al. (2024). Energy-efficient sleep apnea detection using a hyperdimensional computing framework based on wearable bracelet photoplethysmography. *IEEE Transactions on Biomedical Engineering* 71, 2483–2494. doi:10.1109/TBME.2024.3377270

Chen, X., Chen, Y., Ma, W., Fan, X., and Li, Y. (2022). Toward sleep-apnea detection with lightweight multi-scaled fusion network based on single-lead ecg. *Knowledge-Based Systems* 247, 108783. doi:10.1016/j.knosys.2022.108783

Chen, Y., Ma, G., Zhang, M., Yang, S., Yan, J., Zhang, Z., et al. (2023b). Contactless screening for sleep apnea with breathing vibration signals based on modified u-net. *Sleep Medicine* 107, 187–195. doi:10.1016/j.sleep.2023.04.030

Chen, Y., Wang, W., Guo, Y., Zhang, H., Chen, Y., and Xie, L. (2021). A single-center validation of the accuracy of a photoplethysmography-based smartwatch for screening obstructive sleep apnea. *Nature and Science of Sleep* 13, 1533–1544. doi:10.2147/NSS.S323286

Chen, Y., Xiao, H., Huang, M., Zheng, Y., Dong, X., and Chen, G. (2025). Predictive modeling of obstructive sleep apnea using pharyngeal magnetic resonance imaging radiomics and clinical data. *Journal of Clinical Sleep Medicine* 21, 1363–1369. doi:10.5664/jcsm.11706

Cheng, L., Luo, S., Li, B., Liu, R., Zhang, Y., and Zhang, H. (2023). Multiple-instance learning for eeg based osa event detection. *Biomedical Signal Processing and Control* 80, 104358. doi:10.1016/j.bspc.2022.104358

Cheng, S., Wang, C., Yue, K., Li, R., Shen, F., Shuai, W., et al. (2022). Automated sleep apnea detection in snoring signal using long short-term memory neural networks. *Biomedical Signal Processing and Control* 71, 103238. doi:10.1016/j.bspc.2021.103238

Chi, H. T. K., Dabbs-Brown, A., Jurek-Loughrey, A., Mulhall, J., Pham, T. D., Doan, N. P., et al. (2026). Obstructive sleep apnea prediction: A comprehensive review and comparative study. *Machine Learning* 115, 29. doi:10.1007/s10994-025-06937-4

Choksatchawathi, T., Sawadwuthikul, G., Thuwajit, P., Kaewlee, T., Mateepithaktham, T., Saisaard, S., et al. (2024). Apsense: Data-driven algorithm in ppg-based sleep apnea sensing. *IEEE Internet of Things Journal* 11, 33915–33926. doi:10.1109/JIOT.2024.3433500

Choudhury, M., Tanvir, M., Yousuf, M. A., Islam, N., and Uddin, M. Z. (2025). Explainable ai-driven scalogram analysis and optimized transfer learning for sleep apnea detection with single-lead electrocardiograms. *Computers in Biology and Medicine* 187, 109769. doi:10.1016/j.compbiomed.2025.109769

Chu, R., Wei, J., Lu, W., and Chen, Y. (2025). Fine-grained respiratory event detection for sahs screening. *Biomedical Signal Processing and Control* 101, 107147. doi:10.1016/j.bspc.2024.107147

Chu, R., Wei, J., Lu, W., Dong, C., and Chen, Y. (2024). Mfs-dbf: A trustworthy multichannel feature sieve and decision boundary formulation system for obstructive sleep apnea detection. *Computers in Biology and Medicine* 179, 108842. doi:10.1016/j.compbiomed.2024.108842

Dietz-Terjung, S., Martin, A. R., Finnsson, E., Ágústsson, J. S., Helgason, S., Helgadóttir, H., et al. (2021). Proof of principle study: diagnostic accuracy of a novel algorithm for the estimation of sleep stages and disease severity in patients with sleep-disordered breathing based on actigraphy and respiratory inductance plethysmography. *Sleep and Breathing* 25, 1945–1952. doi:10.1007/s11325-021-02316-0

Dong, H., Wu, H., Yang, G., and Zhang, J. (2025). A convolutional neural network for automatic detection of sleep-breathing events using single-channel ecg signals. *Biomedical Signal Processing and Control* 109, 107943. doi:10.1016/j.bspc.2025.107943

Eris, S. B., Bozkurt, M. R., Eris, O., and Bilgin, C. (2025). A huge innovation in diagnosis of obstructive sleep apnea syndrome: With an artificial intelligence-based algorithm, obstructive sleep apnea syndrome can now be diagnosed with pulmonary function test. *IEEE Access* 13, 15376–15389. doi:10.1109/ACCESS.2025.3531501

Fang, L., Cai, J., Huang, Z., Tuohuti, A., and Chen, X. (2025). Assessment of simulated snoring sounds with artificial intelligence for the diagnosis of obstructive sleep apnea. *Sleep Medicine* 125, 100–107. doi:10.1016/j.sleep.2024.11.018

Faridsoltani, S., Rahmani, Z., Chamaani, S., Kashaninasab, F., Khoozan, M., Ghalebandi, M. F., et al. (2026). Noncontact sleep apnea–hypopnea syndrome detection using variational mode decomposition-based respiratory signals and deep neural networks. *IEEE Sensors Journal* 26, 2359–2372. doi:10.1109/JSEN.2025.3635051

Faust, O., Barika, R., Shenfield, A., Ciaccio, E. J., and Acharya, U. R. (2021). Accurate detection of sleep apnea with long short-term memory network based on rr interval signals. *Knowledge-Based Systems* 212, 106591. doi:10.1016/j.knosys.2020.106591

Feng, K., Qin, H., Wu, S., Pan, W., and Liu, G. (2021). A sleep apnea detection method based on unsupervised feature learning and single-lead electrocardiogram. *IEEE Transactions on Instrumentation and Measurement* 70, 1–12. doi:10.1109/TIM.2020.3017246

Frija, J., Millet, J., Béquignon, E., Covali, A., Cathelain, G., Houenou, J., et al. (2025). Proposition of a new, minimally-invasive, software smartphone device to predict sleep apnea and its severity. *Sleep and Breathing* 29, 282. doi:10.1007/s11325-025-03441-w

Ganguly, B., Dasgupta, R., and Dey, D. (2025). A residual deep learning framework for sleep apnea diagnosis from single lead electrocardiogram signals: An explainable artificial intelligence approach. *Engineering Applications of Artificial Intelligence* 148, 110481. doi:10.1016/j.engappai.2025.110481

Garcia-Vicente, C., Gutierrez-Tobal, G. C., Jimenez-Garcia, J., Martin-Montero, A., Gozal, D., and Hornero, R. (2023). Ecg-based convolutional neural network in pediatric obstructive sleep apnea diagnosis. *Computers in Biology and Medicine* 167, 107628. doi:10.1016/j.compbiomed.2023.107628

Garcia-Vicente, C., Gutierrez-Tobal, G. C., Vaquerizo-Villar, F., Martin-Montero, A., Gozal, D., and Hornero, R. (2025). Sleepecg-net: Explainable deep learning approach with ecg for pediatric sleep apnea diagnosis. *IEEE Journal of Biomedical and Health Informatics* 29, 1021–1034. doi:10.1109/JBHI.2024.3495975

Gayen, S., Rao, N. M., Sahu, D. K., Bhardwaj, A., Kumar, P., Sivaraman, J., et al. (2026). Sphe-match: Semi-supervised prototype-aware hierarchical embedding matching approach for classifying sleep apnea events using single-lead electrocardiogram. *Engineering Applications of Artificial Intelligence* 170, 114207. doi:10.1016/j.engappai.2026.114207

Gayen, S., rao, N. M., Sahu, D. K., Sivaraman, J., Pal, K., Vasamsetti, S., et al. (2025). Smartmatch: A semi-supervised framework for obstructive sleep apnea classification using single-lead electrocardiogram signals with limited annotations. *Engineering Applications of Artificial Intelligence* 157, 111226. doi:10.1016/j.engappai.2025.111226

Ge, S., Wu, K., Li, S., Li, R., and Yang, C. (2024). Machine learning methods for adult OSAHS risk prediction. *BMC Health Services Research* 24, 706. doi:10.1186/s12913-024-11081-1

Gu, Z. and Fan, Y. (2025). Ai-enabled osa screening using eeg data analysis and english listening comprehension insights. *Frontiers in Medicine* 12, 1534623. doi:10.3389/fmed.2025.1534623

Gupta, K., Bajaj, V., and Jain, S. (2024). Multiresolution assessment of ecg sensor data for sleep apnea detection using wide neural network. *IEEE Sensors Journal* 24, 12624–12631. doi:10.1109/JSEN.2024.3367776

Gutierrez-Tobal, G. C., Gomez-Pilar, J., Ferreira-Santos, D., Pereira-Rodrigues, P., Alvarez, D., del Campo, F., et al. (2026). A subject-based association network defines new pediatric sleep apnea phenotypes with different odds of recovery after treatment. *Computer Methods and Programs in Biomedicine* 275, 109209. doi:10.1016/j.cmpb.2025.109209

Han, J., Xia, T., and Mascolo, C. (2026). Continuous mobile audio monitoring for sleep apnea detection. *IEEE Journal of Biomedical and Health Informatics* PP, 1–14. doi:10.1109/JBHI.2026.3685448

Hanif, U., Kiaer, E. K., Capasso, R., Liu, S. Y., Mignot, E. J. M., Sorensen, H. B. D., et al. (2023). Automatic scoring of drug-induced sleep endoscopy for obstructive sleep apnea using deep learning. *Sleep Medicine* 102, 19–29. doi:10.1016/j.sleep.2022.12.015

Hanif, U., Leary, E., Schneider, L., Paulsen, R., Morse, A. M., Blackman, A., et al. (2021). Estimation of apnea-hypopnea index using deep learning on 3-d craniofacial scans. *IEEE Journal of Biomedical and Health Informatics* 25, 4185–4194. doi:10.1109/JBHI.2021.3078127

Hassan, O., Paul, T., Amin, N., Titirsha, T., Thakker, R., Parvin, D., et al. (2023). An optimized hardware inference of sabinn: Shift-accumulate binarized neural network for sleep apnea detection. *IEEE Transactions on Instrumentation and Measurement* 72, 1–11. doi:10.1109/TIM.2023.3279880

Hayano, J., Adachi, M., Murakami, Y., Sasaki, F., and Yuda, E. (2025). Detection of sleep apnea using only inertial measurement unit signals from Apple Watch: a pilot-study with machine learning approach. *Sleep and Breathing* 29, 91. doi:10.1007/s11325-025-03255-w

He, S., Li, Y., Zhang, C., Li, Z., Ren, Y., Li, T., et al. (2023). Deep learning technique to detect craniofacial anatomical abnormalities concentrated on middle and anterior of face in patients with sleep apnea. *Sleep Medicine* 112, 12–20. doi:10.1016/j.sleep.2023.09.025

Holfinger, S. J., Lyons, M. M., Keenan, B. T., Mazzotti, D. R., Mindel, J., Maislin, G., et al. (2022). Diagnostic performance of machine learning-derived osa prediction tools in large clinical and community-based samples. *Chest* 161, 807–817

Hossan, I., Dip, M. S. S., Kabir, S., and Motin, M. A. (2025). Deepapneanet: A multistage cnn-bi-lstm hybrid model for sleep apnea detection from single-lead ecg signal. *IEEE Sensors Letters* 9, 1–4. doi:10.1109/LSENS.2025.3558675

Hou, L., Zhuang, Y., Zhang, H., Yang, G., Hua, Z., Chen, K., et al. (2025a). Time-hybrid osaformer (tho): A hybrid temporal sequence transformer for accurate detection of obstructive sleep apnea via single-lead ecg signals. *Computer Methods and Programs in Biomedicine* 260, 108558. doi:10.1016/j.cmpb.2024.108558

Hou, Y., Wang, B., Zhang, C., Wang, Q., Li, J., Meng, P., et al. (2025b). Osasformer: A transformer-based model for osas screening via multi-source representation fusion. *Knowledge-Based Systems* 316, 113365. doi:10.1016/j.knosys.2025.113365

Hsu, Y.-S., Lin, Y.-C., Kuo, Y.-E., Chou, C.-H., Chou, M.-C., Chang, Y., et al. (2026). Patch-type heart rate variability analysis with artificial intelligence for detection of obstructive sleep apnea. *Nature and Science of Sleep* doi:10.2147/NSS.S568569

Hu, S., Wang, Y., Liu, J., and Yang, C. (2025). Xsleepfusion: A dual-stage information bottleneck fusion framework for interpretable multimodal sleep analysis. *Information Fusion* 123, 103275. doi:10.1016/j.inffus.2025.103275

Hu, X., Fang, R., Luo, H., Luo, J., Chen, C., and Chen, W. (2026). A 1d snoring waveform and 2d composite acoustic feature graph-based multi-modal fusion network for obstructive sites recognition. *IEEE Journal of Biomedical and Health Informatics* PP, 1–12. doi:10.1109/JBHI.2026.3670208

Huang, J., Zhuang, J., Zheng, H., Yao, L., Chen, Q., Wang, J., et al. (2024). A machine learning prediction model of adult obstructive sleep apnea based on systematically evaluated common clinical biochemical indicators. *Nature and Science of Sleep* 16, 413–428. doi:10.2147/NSS.S453794

Huttunen, R., Leppänen, T., Duce, B., Arnardottir, E. S., Nikkonen, S., Myllymaa, S., et al. (2023). A comparison of signal combinations for deep learning-based simultaneous sleep staging and respiratory event detection. *IEEE Transactions on Biomedical Engineering* 70, 1704–1714. doi:10.1109/TBME.2022.3225268

Ivanovska, T., Daboul, A., Kalentev, O., Hosten, N., Biffar, R., Völzke, H., et al. (2021). A deep cascaded segmentation of obstructive sleep apnea-relevant organs from sagittal spine MRI. *International Journal of Computer Assisted Radiology and Surgery* 16, 579–588. doi:10.1007/s11548-021-02333-0

Iwasaki, A., Nakayama, C., Fujiwara, K., Sumi, Y., Matsuo, M., Kano, M., et al. (2021). Screening of sleep apnea based on heart rate variability and long short-term memory. *Sleep and Breathing* 25, 1821–1829. doi:10.1007/s11325-020-02249-0

Javeed, A., Berglund, J. S., Dallora, A. L., Saleem, M. A., and Anderberg, P. (2023). Predictive power of XGBoost_BiLSTM model: A machine-learning approach for accurate sleep apnea detection using electronic health data. *International Journal of Computational Intelligence Systems* 16, 188. doi:10.1007/s44196-023-00362-y

Ji, Y., Chen, D., Zuo, Y., Gao, T., and Tang, Y. (2024). Accurate apnea and hypopnea localization in psg with multi-scale object detection via dual-modal feature learning. *Biomedical Signal Processing and Control* 89, 105717. doi:10.1016/j.bspc.2023.105717

Jiang, L., Li, M., and Jiang, J. (2025a). Optimizing apnea–hypopnea event detection in pediatric osahs diagnosis: An enhanced genetic algorithm approach. *Biomedical Signal Processing and Control* 103, 107428. doi:10.1016/j.bspc.2024.107428

Jiang, M., Lou, L., Zhang, W., Yang, X., Wang, Z., Wu, Y., et al. (2025b). Sleep apnea syndrome classification based om temporal ecg and spo2 by using multimodal multichannel transfer module with squeeze and excitation. *Biomedical Signal Processing and Control* 104, 107589. doi:10.1016/j.bspc.2025.107589

Jiang, Y., Peng, J., and Song, L. (2021). An osahs evaluation method based on multi-features acoustic analysis of snoring sounds. *Sleep Medicine* 84, 317–323. doi:10.1016/j.sleep.2021.06.012

Jiao, M., Song, C., Xian, X., Yang, S., and Liu, F. (2024). Deep attention networks with multi-temporal information fusion for sleep apnea detection. *IEEE Open Journal of Engineering in Medicine and Biology* 5, 792–802. doi:10.1109/OJEMB.2024.3405666

Jimenez-Garcia, J., Garcia, M., Gutierrez-Tobal, G. C., Kheirandish-Gozal, L., Vaquerizo-Villar, F., Alvarez, D., et al. (2022). A 2d convolutional neural network to detect sleep apnea in children using airflow and oximetry. *Computers in Biology and Medicine* 147, 105784. doi:10.1016/j.compbiomed.2022.105784

Jimenez-Garcia, J., Garcia, M., Gutierrez-Tobal, G. C., Kheirandish-Gozal, L., Vaquerizo-Villar, F., Alvarez, D., et al. (2024). An explainable deep-learning architecture for pediatric sleep apnea identification from overnight airflow and oximetry signals. *Biomedical Signal Processing and Control* 87, 105490. doi:10.1016/j.bspc.2023.105490

Jirakittayakorn, N., Manupibul, U., Wongsawat, Y., and Mitrirattanakul, S. (2024). Respnet: A dual-network approach for automated osa severity classification utilizing psg type iii signals. *IEEE Access* 12, 148538–148552. doi:10.1109/ACCESS.2024.3477266

Juang, C.-F., Wen, C.-Y., Chang, K.-M., Chen, Y.-H., Wu, M.-F., and Huang, W.-C. (2021). Explainable fuzzy neural network with easy-to-obtain physiological features for screening obstructive sleep apnea-hypopnea syndrome. *Sleep Medicine* 85, 280–290. doi:10.1016/j.sleep.2021.07.012

Kamal, A., Wu, Y., Aljuaid, H., Najam, S., Alnowaiser, K., Jalal, A., et al. (2026). Sleepcode: A novel attention-driven multimodal framework for sleep staging and obstructive apnea screening. *IEEE Transactions on Consumer Electronics* doi:10.1109/TCE.2026.3674648. Early access / accepted version

Khan, A., Biswas, S. K., Chunka, C., and Baruah, B. (2025). Intelligent sleep apnea detection by advanced ml using single-lead eeg signal data. *IEEE Sensors Journal* 25, 3859–3866. doi:10.1109/JSEN.2024.3506057

Khan, A., Biswas, S. K., Chunka, C., and Das, A. K. (2024). A machine learning model for obstructive sleep apnea detection using ensemble learning and single-lead eeg signal data. *IEEE Sensors Journal* 24, 20266–20273. doi:10.1109/JSEN.2024.3397395

Kim, D., Han, J. Y., Jung, H., Song, D. Y., Lee, C., Ryu, G., et al. (2025a). AI-enhanced smartwatch AHI estimation and AI-scored polysomnography for obstructive sleep apnea: Real-world validation. *Nature and Science of Sleep* 17, 2297–2307. doi:10.2147/NSS.S540460

Kim, D., Park, J. Y., Song, Y. W., Kim, E., Kim, S., and Joo, E. Y. (2024). Machine-learning-based classification of obstructive sleep apnea using 19-channel sleep eeg data. *Sleep Medicine* 124, 323–330. doi:10.1016/j.sleep.2024.09.041

Kim, H., Lee, H., and Kim, M. (2025b). Sleepwatcher: Detecting sleep apnea/hypopnea syndrome from wearable devices using deep learning. *Biomedical Signal Processing and Control* 103, 107460. doi:10.1016/j.bspc.2024.107460

Kim, Y., Kim, M., Shin, J., and Ko, M. (2025c). Apneawhisper: Transformer-based audio segmentation for fine-grained non-invasive sleep apnea detection. *Nature and Science of Sleep* 17, 2455–2468. doi:10.2147/NSS.S553774

Koda, T., Okumura, S., Taki, H., Hamada, S., Sunadome, H., Sato, S., et al. (2024). Noncontact detection of sleep apnea using radar and expectation–maximization algorithm. *IEEE Sensors Journal* 24, 32748–32756. doi:10.1109/JSEN.2024.3450890

Kuhn, J., Schiphorst, L. R. B., Wulterkens, B. M., Asin, J., Overeem, S., van Gilst, M. M., et al. (2026). Sleep apnea phenotypes based on multi-night assessment of sleep perception. *Behavioral Sleep Medicine* doi:10.1080/15402002.2026.2630936

Kumar, C. B., Mondal, A. K., Bhatia, M., Panigrahi, B. K., and Gandhi, T. K. (2023). Self-supervised representation learning-based osa detection method using single-channel ecg signals. *IEEE Transactions on Instrumentation and Measurement* 72, 1–15. doi:10.1109/TIM.2023.3261931

Kwon, H. B., Jun, K. H., Yoon, H., Joo, E. Y., and Choi, S. H. (2026). Unconstrained sleep apnea detection with conv-vit network: Lora tuning for personalized monitoring. *IEEE Sensors Journal* 26, 6331–6343. doi:10.1109/JSEN.2025.3650450

Kwon, H. B., Son, D., Lee, D., Yoon, H., Lee, M. H., Lee, Y. J., et al. (2022). Hybrid cnn-lstm network for real-time apnea-hypopnea event detection based on ir-uwb radar. *IEEE Access* 10, 17556–17564. doi:10.1109/ACCESS.2021.3081747

Lai, W.-S., Li, T.-W., Kuo, C.-F. J., and Liu, S.-C. (2025). AI-assisted awake endoscopic video analysis for obstructive sleep apnea detection. *Nature and Science of Sleep* 17, 2807–2818. doi:10.2147/NSS.S559788

Lee, C.-H., Kim, H., Yoon, B. C., and Kim, D.-J. (2025a). Toward foundational model for sleep analysis using a multimodal hybrid-self-supervised learning framework. *IEEE Transactions on Cybernetics* 55, 5619–5632. doi:10.1109/TCYB.2025.3603608

Lee, K.-T., Liu, W.-T., Lin, Y.-C., Chen, Z., Ho, Y.-H., Huang, Y.-W., et al. (2025b). Utilizing a wireless radar framework in combination with deep learning approaches to evaluate obstructive sleep apnea severity in home-setting environments. *Journal of Multidisciplinary Healthcare* 18, 381–393. doi:10.2147/JMDH.S486261

Lee, M.-H., Lee, S. K., Thomas, R. J., Yoon, J.-E., Yun, C.-H., and Shin, C. (2021). Deep learning-based assessment of brain connectivity related to obstructive sleep apnea and daytime sleepiness. *Nature and Science of Sleep* 13, 1561–1572. doi:10.2147/NSS.S327110

Leger, D. and Elbaz, M. (2024). Diagnosing OSA and insomnia at home based only on an actigraphy total sleep time and RIP belts an algorithm "nox body sleep". *Nature and Science of Sleep* 16, 833–845. doi:10.2147/NSS.S431650

Leino, A., Nikkonen, S., Kainulainen, S., Korkalainen, H., Toyras, J., Myllymaa, S., et al. (2021). Neural network analysis of nocturnal spo2 signal enables easy screening of sleep apnea in patients with acute cerebrovascular disease. *Sleep Medicine* 79, 71–78. doi:10.1016/j.sleep.2020.12.032

Li, F., Xu, Y., Chen, J., Lu, P., Zhang, B., and Cong, F. (2023a). A deep learning model developed for sleep apnea detection: A multi-center study. *Biomedical Signal Processing and Control* 85, 104689. doi:10.1016/j.bspc.2023.104689

Li, H., Hong, J., Zhang, Y., Li, L., Long, T., Huang, L., et al. (2025a). Machine learning classification based on individual whole-brain functional connectivity in male OSA patients. *Nature and Science of Sleep* 17, 959–973. doi:10.2147/NSS.S504512

Li, H., Lu, Y., Qian, Y., Zhou, L., Wang, M., and Cheng, H. (2026). Learning generalizable representations with adversarial domain adaptation for snoring-based sleep apnea detection. *Biomedical Signal Processing and Control* 113, 108937. doi:10.1016/j.bspc.2025.108937

Li, L., Song, L., Liu, Y., Ayoub, M., Song, Y., Shu, Y., et al. (2025b). Combining static and dynamic functional connectivity analyses to identify male patients with obstructive sleep apnea and predict clinical symptoms. *Sleep Medicine* 126, 136–147. doi:10.1016/j.sleep.2024.12.013

Li, R., Li, W., and Yue, K. (2023b). Convolutional neural network for screening of obstructive sleep apnea using snoring sounds. *Biomedical Signal Processing and Control* 86, 104966. doi:10.1016/j.bspc.2023.104966

Lin, X., Cheng, H., Lu, Y., Luo, H., Li, H., Qian, Y., et al. (2022a). Contactless sleep apnea detection in snoring signals using hybrid deep neural networks targeted for embedded hardware platform with real-time applications. *Biomedical Signal Processing and Control* 77, 103765. doi:10.1016/j.bspc.2022.103765

Lin, X.-X., Lin, P., Yeh, E.-H., Liu, G.-R., Lien, W.-C., and Fang, Y. (2023). Rapidest: A framework for obstructive sleep apnea detection. *IEEE Transactions on Neural Systems and Rehabilitation Engineering* 31, 387–397. doi:10.1109/TNSRE.2022.3224474

Lin, Y.-S., Wu, Y.-P., Wu, Y.-C., Lee, P.-L., and Yang, C.-H. (2022b). Achieving accurate automatic sleep apnea/hypopnea syndrome assessment using nasal pressure signal. *IEEE Journal of Biomedical and Health Informatics* 26, 5473–5481. doi:10.1109/JBHI.2022.3199454

Liu, A., Lu, Q., Huang, X., Li, H., Hou, Z., Lin, W., et al. (2026a). Integrative assessment of cephalometric and polysomnographic features in paediatric obstructive sleep apnea: A cross-sectional study. *Nature and Science of Sleep* doi:10.2147/NSS.S574045

Liu, H., Cui, S., Zhao, X., and Cong, F. (2023). Detection of obstructive sleep apnea from single-channel ecg signals using a cnn-transformer architecture. *Biomedical Signal Processing and Control* 82, 104581. doi:10.1016/j.bspc.2023.104581

Liu, M.-H., Chien, S.-Y., Wu, Y.-L., Sun, T.-H., Huang, C.-S., Hsu, K.-C., et al. (2024a). EfficientNet-based machine learning architecture for sleep apnea identification in clinical single-lead ECG signal data sets. *BioMedical Engineering OnLine* 23, 57. doi:10.1186/s12938-024-01252-w

Liu, R., Li, C., Xu, H., Wu, K., Li, X., Liu, Y., et al. (2022). Fusion of whole night features and desaturation segments combined with feature extraction for event-level screening of sleep-disordered breathing. *Nature and Science of Sleep* 14, 927–940. doi:10.2147/NSS.S355369

Liu, T., Que, L., Bai, W., and Yao, H. (2026b). Machine learning optimization of obstructive sleep apnea screening: development and validation of a gradient boosting prediction model with a clinical implementation framework. *Frontiers in Medicine* 13, 1775766. doi:10.3389/fmed.2026.1775766

Liu, Y., Li, Y., Zhao, M., Xiong, N., Guan, M., Li, H.-C., et al. (2026c). Dfcnet: A precise detection approach for obstructive sleep apnea-hypopnea events using airflow and respiratory effort signals. *IEEE Journal of Biomedical and Health Informatics* PP, 1–11. doi:10.1109/JBHI.2026.3688540

Liu, Y., Xie, S. Q., Yang, X., Chen, J. L., and Zhou, J. R. (2024b). Development and validation of a nomogram for predicting obstructive sleep apnea severity in children. *Nature and Science of Sleep* 16, 193–206. doi:10.2147/NSS.S445469

Liu, Y., Xin, H., Shu, Y., Li, L., Long, T., Zeng, L., et al. (2024c). Altered density of resting-state long- and short-range functional connectivity in patients with moderate-to-severe obstructive sleep apnea. *Nature and Science of Sleep* 16, 1891–1904. doi:10.2147/NSS.S483030

Ma, H., Wang, H., Zhu, Y., Liang, L., Yang, X., Chen, S., et al. (2026). Utility of gait biomarkers in screening for obstructive sleep apnea among patients with parkinson's disease: a cross-sectional study. *BMC Neurology* 26, 169. doi:10.1186/s12883-026-04700-3

Mahmud, T., Khan, I. A., Mahmud, T. I., Fattah, S. A., Zhu, W.-P., and Ahmad, M. O. (2021). Sleep apnea detection from variational mode decomposed eeg signal using a hybrid cnn-bilstm. *IEEE Access* 9, 102355–102367. doi:10.1109/ACCESS.2021.3097090

Mandeville, R., Sedghamiz, H., Mansfield, P., Sheean, G., Studer, C., Cordice, D., et al. (2024). Deep learning enhanced transmembranous electromyography in the diagnosis of sleep apnea. *BMC Neuroscience* 25, 80. doi:10.1186/s12868-024-00913-9

Mashrur, F. R., Islam, M. S., Saha, D. K., Islam, S. M. R., and Moni, M. A. (2021). Scnn: Scalogram-based convolutional neural network to detect obstructive sleep apnea using single-lead electrocardiogram signals. *Computers in Biology and Medicine* 134, 104532. doi:10.1016/j.compbiomed.2021.104532

Midelet, A., Prigent, A., Schaeffer, M.-C., Hy, R. L., Pepin, J.-L., and Bailly, S. (2023). Features of cheyne-stokes respiration automatically extracted from cpap airflow signal raw data: Identification of discriminating features to detect heart failure. *Biomedical Signal Processing and Control* 86, 105227. doi:10.1016/j.bspc.2023.105227

Monna, F., Messaoud, R. B., Navarro, N., Baillieul, S., Sanchez, L., Loiodice, C., et al. (2022). Machine learning and geometric morphometrics to predict obstructive sleep apnea from 3d craniofacial scans. *Sleep Medicine* 95, 76–83. doi:10.1016/j.sleep.2022.04.019

Moradhasel, B., Sheikhani, A., Aloosh, O., and Dabanloo, N. J. (2023). Spectrogram classification of patient chin electromyography based on deep learning: A novel method for accurate diagnosis obstructive sleep apnea. *Biomedical Signal Processing and Control* 79, 104215. doi:10.1016/j.bspc.2022.104215

Mosteiro-Añón, M., Casal-Guisande, M., Fernández-Villar, A., and Torres-Durán, M. (2025). AI-driven clinical decision support for early diagnosis and treatment planning in patients with suspected sleep apnea using clinical and demographic data before sleep studies. *npj Primary Care Respiratory Medicine* 35, 51. doi:10.1038/s41533-025-00455-5

Nassehi, F., Eken, A., Atalay, N. B., Firat, H., and Erogul, O. (2024). Machine learning based severity classification of obstructive sleep apnea patients using awake eeg. *Biomedical Signal Processing and Control* 96, 106566. doi:10.1016/j.bspc.2024.106566

Nassi, T. E., Ganglberger, W., Sun, H., Bucklin, A. A., Biswal, S., Putten, M. J. A. M. V., et al. (2022). Automated scoring of respiratory events in sleep with a single effort belt and deep neural networks. *IEEE Transactions on Biomedical Engineering* 69, 2094–2104. doi:10.1109/TBME.2021.3136753

Nguyen, H. X., Nguyen, D. V., Pham, H. H., and Do, C. D. (2024). Mpcnn: A novel matrix profile approach for cnn-based single lead sleep apnea in classification problem. *IEEE Journal of Biomedical and Health Informatics* 28, 4878–4890. doi:10.1109/JBHI.2024.3397653

Niemi, T., Kanniainen, M., Nurmo, M., Pukkila, T., Solhjoo, S., and Räsänen, E. (2026). Detecting sleep apnea using non-linear measures of heart rate variability. *Respiratory Research* 27, 185. doi:10.1186/s12931-026-03621-6

Nikkonen, S., Korkalainen, H., Leino, A., Myllymaa, S., Duce, B., Leppänen, T., et al. (2021). Automatic respiratory event scoring in obstructive sleep apnea using a long short-term memory neural network. *IEEE Journal of Biomedical and Health Informatics* 25, 2917–2927. doi:10.1109/JBHI.2021.3064694

Olsen, M., Zeitzer, J. M., Nakase-Richardson, R., Musgrave, V. H., Sorensen, H. B. D., Mignot, E., et al. (2024). A deep transfer learning approach for sleep stage classification and sleep apnea detection using wrist-worn consumer sleep technologies. *IEEE Transactions on Biomedical Engineering* 71, 2506–2517. doi:10.1109/TBME.2024.3378480

Padovano, D., Martinez-Rodrigo, A., Pastor, J. M., Rieta, J. J., and Alcaraz, R. (2022). On the generalization of sleep apnea detection methods based on heart rate variability and machine learning. *IEEE Access* 10, 92710–92725. doi:10.1109/ACCESS.2022.3201911

Pan, Y., Yang, J., Zhang, T., Wen, J., Pang, F., and Luo, Y. (2021). Characterization of the abnormal cortical effective connectivity in patients with sleep apnea hypopnea syndrome during sleep. *Computer Methods and Programs in Biomedicine* 204, 106060. doi:10.1016/j.cmpb.2021.106060

Parbat, D. and Chakraborty, M. (2024). Multiscale entropy analysis of single lead ecg and ecg derived respiration for ai based prediction of sleep apnea events. *Biomedical Signal Processing and Control* 87, 105444. doi:10.1016/j.bspc.2023.105444

Pataka, A., Pepin, J., Bonsignore, M., Schiza, S., Saaresranta, T., Bouloukaki, I., et al. (2024). Sleep apnoea phenotypes in women: A cluster analysis from the esada cohort. *Sleep Medicine* 124, 494–501. doi:10.1016/j.sleep.2024.10.015

Peng, D., Yue, H., Tan, W., Lei, W., Chen, G., Shi, W., et al. (2024). A bimodal feature fusion convolutional neural network for detecting obstructive sleep apnea/hypopnea from nasal airflow and oximetry signals. *Artificial Intelligence In Medicine* 150, 102808. doi:10.1016/j.artmed.2024.102808

Pham, D. T. and Moucek, R. (2025). Efficient sleep apnea detection using single-lead ecg: A cnn-transformer-lstm approach. *Computers in Biology and Medicine* 196, 110655. doi:10.1016/j.compbiomed.2025.110655

Qi, X., Chen, L., Liu, H., Tang, S., He, Z., Dai, Y., et al. (2026). Sleepecgfusion: A cross-modal deep-learning framework for automated sleep staging and sleep-apnea detection. *Knowledge-Based Systems* 334, 115132. doi:10.1016/j.knosys.2025.115132

Qin, H. and Liu, G. (2022). A dual-model deep learning method for sleep apnea detection based on representation learning and temporal dependence. *Neurocomputing* 473, 24–36. doi:10.1016/j.neucom.2021.12.001

Qiu, W., Chen, Z., Lu, Y., Wang, C., and Liu, G. (2025). An ordinal pattern similarity-guided supervised prototype contrastive learning framework with enhanced token selection module for sleep apnea detection based on wearable ppg bracelet. *IEEE Transactions on Consumer Electronics* 71, 9820–9831. doi:10.1109/TCE.2025.3627222

Rajesh, K., Dhuli, R., and Kumar, T. S. (2021). Obstructive sleep apnea detection using discrete wavelet transform-based statistical features. *Computers in Biology and Medicine* 130, 104199. doi:10.1016/j.compbiomed.2020.104199

Rehman, A., Moussa, M., Saleh, H., Khraibi, A., and Khandoker, A. H. (2025). Chin electromyography-based motor unit decomposition for alternative screening of obstructive sleep apnea events: A comprehensive analysis. *Engineering Applications of Artificial Intelligence* 139, 109534. doi:10.1016/j.engappai.2024.109534

Rehman, A. and Saleh, H. (2025). Ultra-low-power system-on-chip for automated screening of central apnea and hypopnea via chin electromyography. *Computers in Biology and Medicine* 193, 110349. doi:10.1016/j.compbiomed.2025.110349

Retamales, G., Gavidia, M. E., Bausch, B., Montanari, A. N., Husch, A., and Goncalves, J. (2024). Towards automatic home-based sleep apnea estimation using deep learning. *npj Digital Medicine* 7, 144. doi:10.1038/s41746-024-01139-z

Rohrscheib, C., Moura, A. A., Raphelson, J., Orr, J. E., Patel, R. P., and Malhotra, A. (2026). Evaluation of an automated sleep apnea scoring algorithm via the wesper lab home sleep apnea test. *Sleep Medicine* 141, 108828. doi:10.1016/j.sleep.2026.108828

Romero, H. E., Ma, N., Brown, G. J., and Hill, E. A. (2022). Acoustic screening for obstructive sleep apnea in home environments based on deep neural networks. *IEEE Journal of Biomedical and Health Informatics* 26, 2941–2950. doi:10.1109/JBHI.2022.3154719

Ryu, S., Kim, J. H., Yu, H., Jung, H.-D., Chang, S. W., Park, J. J., et al. (2021). Diagnosis of obstructive sleep apnea with prediction of flow characteristics according to airway morphology automatically extracted from medical images: Computational fluid dynamics and artificial intelligence approach. *Computer Methods and Programs in Biomedicine* 208, 106243. doi:10.1016/j.cmpb.2021.106243

Saha, S., Ghahjaverestan, N. M., and Yadollahi, A. (2025). Separating obstructive and central respiratory events during sleep using breathing sounds: Utilizing transfer learning on deep convolutional networks. *Sleep Medicine* 131, 106485. doi:10.1016/j.sleep.2025.106485

Seo, D.-W., Kim, J., Lee, H.-W., and Suh, Y.-K. (2023). A deep neural network based wake-after-sleep-onset time aware sleep apnea severity estimation scheme using single-lead ecg data. *IEEE Access* 11, 43720–43732. doi:10.1109/ACCESS.2023.3272373

Shao, S., Han, G., Wang, T., Song, C., Yao, C., and Hou, J. (2022). Obstructive sleep apnea detection scheme based on manually generated features and parallel heterogeneous deep learning model under iomt. *IEEE Journal of Biomedical and Health Informatics* 26, 5841–5850. doi:10.1109/JBHI.2022.3166859

Sharma, M., Kumbhani, D., Tiwari, J., Kumar, T. S., and Acharya, U. R. (2022). Automated detection of obstructive sleep apnea in more than 8000 subjects using frequency optimized orthogonal wavelet filter bank with respiratory and oximetry signals. *Computers in Biology and Medicine* 144, 105364. doi:10.1016/j.compbiomed.2022.105364

Shen, Q., Liu, G., Wang, Z., Liu, X., and Xin, J. (2025). Multi-task self-supervised learning framework via contrastive restoration for sleep apnea syndrome detection using single-lead ecg. *Biomedical Signal Processing and Control* 105, 107631. doi:10.1016/j.bspc.2025.107631

Shi, Y., Zhang, Y., Cao, Z., Ma, L., Yuan, Y., Niu, X., et al. (2023). Application and interpretation of machine learning models in predicting the risk of severe obstructive sleep apnea in adults. *BMC Medical Informatics and Decision Making* 23, 230. doi:10.1186/s12911-023-02331-z

Sillaparaya, A., Jiraraksopakun, Y., Chamnongthai, K., and Bhatranand, A. (2025). Attention-based transfer learning for efficient obstructive sleep apnea (osa) classification on snore sound. *IEEE Access* 13, 113879–113889. doi:10.1109/ACCESS.2025.3575203

Sinha, N. and Das, A. (2022). Detection of obstructive sleep apnea using non-negative matrix factorization-based feature extraction approach in eigen spectrum domain. *IEEE Transactions on Instrumentation and Measurement* 71, 1–9. doi:10.1109/TIM.2022.3151167

Song, Y., Ding, L., Peng, J., Song, L., and Zhang, X. (2025). Screening for obstructive sleep apnea hypopnea using sleep breathing sounds based on the psg-audio dataset. *Biomedical Signal Processing and Control* 103, 107472. doi:10.1016/j.bspc.2024.107472

Srivastava, G., Chauhan, A., Kargeti, N., Pradhan, N., and Dhaka, V. S. (2023). Apneanet: A hybrid 1dcnn-lstm architecture for detection of obstructive sleep apnea using digitized ecg signals. *Biomedical Signal Processing and Control* 84, 104754. doi:10.1016/j.bspc.2023.104754

Strumpf, Z. B., Jacono, F. J., Kaffashi, F., Patil, S. P., Strohl, K. P., and Loparo, K. A. (2026). Spectral analysis of ECG and SpO_2 for machine learning classification of sleep-disordered breathing. *Sleep and Breathing* 30, 41. doi:10.1007/s11325-026-03602-5

Su, Y., Wang, Z., Chang, H., Zhu, S., Zhou, Y., Cao, Z., et al. (2024). Craniofacial development characteristics in children with obstructive sleep apnea for establishment and external validation of the prediction model. *Nature and Science of Sleep* 16, 2151–2170. doi:10.2147/NSS.S492714

TaghiBeyglou, B., Geng, J., McDaulid, D., Gnaneswaran, P., Yasokaran, O., Chow, A., et al. (2026). Self-supervised speech representations for sleep apnea severity prediction. *IEEE Transactions on Biomedical Engineering* PP, 1–11. doi:10.1109/TBME.2026.3656326

Taghizadegan, Y., Dabanloo, N. J., and Maghooli, K. (2021). Obstructive sleep apnea event prediction using recurrence plots and convolutional neural networks (rp-cnns) from polysomnographic signals. *Biomedical Signal Processing and Control* 69, 102928. doi:10.1016/j.bspc.2021.102928

Talukder, A., Li, Y., Yeung, D., Shi, M., Umbach, D. M., Fan, Z., et al. (2024). Osapredictor: A tool for prediction of moderate to severe obstructive sleep apnea-hypopnea using readily available patient characteristics. *Computers in Biology and Medicine* 178, 108777. doi:10.1016/j.compbiomed.2024.108777

Tan, J., Chen, W., Yu, D., Peng, T., Li, C., and Lv, K. (2025). Artificial intelligence screening tool for obstructive sleep apnoea: A study based on outpatients at a sleep medical centre. *Nature and Science of Sleep* 17, 425–434. doi:10.2147/NSS.S503124

Tang, L. and Liu, G. (2021). The novel approach of temporal dependency complexity analysis of heart rate variability in obstructive sleep apnea. *Computers in Biology and Medicine* 135, 104632. doi:10.1016/j.compbiomed.2021.104632

Tang, X., Zhang, B., Li, Y., Guan, M., Xiong, N., Li, H.-C., et al. (2026). Hybrid residual neural network for obstructive sleep apnea detection using ecg scalogram. *IEEE Transactions on Biomedical Engineering* 73, 1015–1023. doi:10.1109/TBME.2025.3597083

Tani, J., Huang, C.-S., Aurora, Y., Chung, Y.-C., Ho, S.-J., Lee, P.-H., et al. (2026). Validation of an artificial intelligence-driven novel microphone-based wireless jaw movement and respiratory event detection system for home sleep apnea testing. *Biomedical Signal Processing and Control* 120, 110008. doi:10.1016/j.bspc.2026.110008

Thompson, S., Reilly, D., Fergus, P., and Chalmers, C. (2024). Detection of obstructive sleep apnoea using features extracted from segmented time-series ecg signals with a one dimensional convolutional neural network. *IEEE Access* 12, 1076–1091. doi:10.1109/ACCESS.2023.3346689

Thuptimdang, W., Tawaranurak, K., Panyarath, P., Rakim, W., Wae-asae, K., and Khoo, M. C. K. (2026). Towards automatic home screening of obstructive sleep apnea using combined features from pulse wave amplitude, pulse-to-pulse interval and oxygen desaturation. *Sleep and Breathing* 30, 57. doi:10.1007/s11325-026-03592-4

Tian, S., Xia, Y., and Zhang, Y. (2026). Hamce-net: Heterogeneity-aware multimodal cross-epoch network for sleep apnea detection. *IEEE Access* 14, 62075–62086. doi:10.1109/ACCESS.2026.3686169

Tirachaimongkol, V., Banhiran, W., Chotinaiwattarakul, W., Pimolsri, C., Srikajon, J., Kasemsuk, N., et al. (2025). Belun sleep platform versus in-lab polysomnography for obstructive sleep apnea diagnosis. *Sleep and Breathing* 29, 266. doi:10.1007/s11325-025-03433-w

Trang, N. T. H., Linh, T. T. D., Vu, D. Q., Loan, B. T. H., Vinh, N. N., and Dang, T. N. (2025). Artificial intelligence-based approaches for sleep-related breathing events identification using EEG and ECG signals. *Sleep and Breathing* 29, 276. doi:10.1007/s11325-025-03442-9

Tsuiki, S., Nagaoka, T., Fukuda, T., Sakamoto, Y., Almeida, F. R., Nakayama, H., et al. (2021). Machine learning for image-based detection of patients with obstructive sleep apnea: an exploratory study. *Sleep and Breathing* 25, 2297–2305. doi:10.1007/s11325-021-02301-7

Tyagi, P. K. and Agrawal, D. (2023). Automatic detection of sleep apnea from single-lead ecg signal using enhanced-deep belief network model. *Biomedical Signal Processing and Control* 80, 104401

Tyagi, P. K. and Agrawal, D. (2024). Automatic detection of sleep apnea from a single-lead ecg signal based on spiking neural network model. *Computers in biology and medicine* 179, 108877

Ullah, N., Mahmood, T., Kim, S. G., Nam, S. H., Sultan, H., and Park, K. R. (2023). Dcda-net: Dual-convolutional dual-attention network for obstructive sleep apnea diagnosis from single-lead electrocardiograms. *Engineering Applications of Artificial Intelligence* 123, 106451

Ullah, N., Sultan, H., Hong, J. S., Kim, S. G., Akram, R., and Park, K. R. (2025). Convolutional self-attention with adaptive channel-attention network for obstructive sleep apnea detection using limited training data. *Engineering Applications of Artificial Intelligence* 156, 111154. doi:10.1016/j.engappai.2025.111154

Wan, C., Nnamdi, M. C., Shi, W., Smith, B., Purnell, C., and Wang, M. D. (2025). Advancing sleep disorder diagnostics: A transformer-based eeg model for sleep stage classification and osa prediction. *IEEE Journal of Biomedical and Health Informatics* 29, 878–886. doi:10.1109/JBHI.2024.3512616

Wang, B., Tang, X., Ai, H., Li, Y., Xu, W., Wang, X., et al. (2022). Obstructive sleep apnea detection based on sleep sounds via deep learning. *Nature and Science of Sleep* 14, 2033–2045. doi:10.2147/NSS.S373367

Wang, P., Lin, Y.-C., Liu, W.-T., Ma, E., Majumdar, A., Kang, J.-H., et al. (2026a). Deep representation learning with cross attention-based multi-feature fusion for sleep apnea detection using sleep respiratory sound. *Biomedical Signal Processing and Control* 113, 109093. doi:10.1016/j.bspc.2025.109093

Wang, Y., Ji, S., Yang, T., Wang, X., Wang, H., and Zhao, X. (2021). An efficient method to detect sleep hypopnea–apnea events based on eeg signals. *IEEE Access* 9, 641–650. doi:10.1109/ACCESS.2020.3038486

Wang, Y., Liu, Q., Min, F., and Wang, H. (2026b). Psg-mae: Robust multitask sleep event monitoring using multichannel psg reconstruction and inter-channel contrastive learning. *IEEE Transactions on Neural Systems and Rehabilitation Engineering* 34, 274–286. doi:10.1109/TNSRE.2025.3645353

Wang, Z., Pan, X., Mei, Z., Xu, Z., Lv, Y., Zhang, Y., et al. (2024). Ecgan-assisted rest-net based on fuzziness for osa detection. *IEEE Transactions on Biomedical Engineering* 71, 2518–2527. doi:10.1109/TBME.2024.3378508

Wang, Z., Yang, T., Zhu, Y., Liu, J., Cistulli, P. A., and Chen, W. (2025). Multi-task learning for osa detection and sleep staging via multi-scale modeling. *IEEE Journal of Biomedical and Health Informatics* doi:10.1109/JBHI.2025.3647317. Early access

Wei, K., Zou, L., Liu, G., and Wang, C. (2023). Ms-net: Sleep apnea detection in ppg using multi-scale block and shadow module one-dimensional convolutional neural network. *Computers in Biology and Medicine* 155, 106469. doi:10.1016/j.compbiomed.2022.106469

Weng, P., Wei, K., Chen, T., Chen, M., and Liu, G. (2022). Fuzzy approximate entropy of extrema based on multiple moving averages as a novel approach in obstructive sleep apnea screening. *IEEE Journal of Translational Engineering in Health and Medicine* 10, 4901211. doi:10.1109/JTEHM.2022.3197084

Werthen-Brabants, L., Castillo-Escario, Y., Groenendaal, W., Jane, R., Dhaene, T., and Deschrijver, D. (2025). Deep learning-based event counting for apnea-hypopnea index estimation using recursive spiking neural networks. *IEEE Transactions on Biomedical Engineering* 72, 1306–1315. doi:10.1109/TBME.2024.3498097

Wu, S., Chen, M., and Wei, K. (2021a). Sleep apnea screening based on photoplethysmography data from wearable bracelets using an information-based similarity approach. *Computer Methods and Programs in Biomedicine* 211, 106442. doi:10.1016/j.cmpb.2021.106442

Wu, S., Liang, D., and Yang, Q. (2021b). Regularity of heart rate fluctuations analysis in obstructive sleep apnea patients using information-based similarity. *Biomedical Signal Processing and Control* 65, 102370. doi:10.1016/j.bspc.2020.102370

Wu, W., Yu, Y., Wang, Q., Liu, D., and Yuan, X. (2021c). Upper airway segmentation based on the attention mechanism of weak feature regions. *IEEE Access* 9, 95372–95381. doi:10.1109/ACCESS.2021.3094032

Wu, Y., Pang, X., Zhao, G., Yue, H., Lei, W., and Wang, Y. (2021d). A novel approach to diagnose sleep apnea using enhanced frequency extraction network. *Computer Methods and Programs in Biomedicine* 206, 106119. doi:10.1016/j.cmpb.2021.106119

Wu, Z., Tan, J., Wu, X., Li, Y., Huang, S., Tan, S., et al. (2026). A unified neural network framework for synchronized pressure control in home ventilators. *Engineering Applications of Artificial Intelligence* 174, 114541. doi:10.1016/j.engappai.2026.114541

Xie, J., Fonseca, P., van Dijk, J., Overeem, S., and Long, X. (2023). Assessment of obstructive sleep apnea severity using audio-based snoring features. *Biomedical Signal Processing and Control* 86, 104942. doi:10.1016/j.bspc.2023.104942

Xie, J., Fonseca, P., van Dijk, J., Overeem, S., and Long, X. (2024). A multi-task learning model using RR intervals and respiratory effort to assess sleep disordered breathing. *BioMedical Engineering OnLine* 23, 49. doi:10.1186/s12938-024-01240-0

Xu, Y., Ou, Q., Cheng, Y., Lao, M., and Pei, G. (2023). Comparative study of a wearable intelligent sleep monitor and polysomnography monitor for the diagnosis of obstructive sleep apnea. *Sleep and Breathing* 27, 205–212. doi:10.1007/s11325-022-02599-x

Xue, B., Wang, Z., Shao, Y., Zhu, X., Zhao, H., Fu, C., et al. (2025). Knowledge guided articulatory and spectrum information fusion for obstructive sleep apnea severity estimation. *IEEE Journal of Biomedical and Health Informatics* PP, 1–12. doi:10.1109/JBHI.2025.3576788

Yamane, T., Fujii, M., and Morita, M. (2025). Clinical-level screening of sleep apnea syndrome with single-lead ECG alone is achievable using machine learning with appropriate time windows. *Sleep and Breathing* 29, 156. doi:10.1007/s11325-025-03316-0

Yang, Q., Zou, L., Wei, K., and Liu, G. (2022). Obstructive sleep apnea detection from single-lead electrocardiogram signals using one-dimensional squeeze-and-excitation residual group network. *Computers in Biology and Medicine* 140, 105124. doi:10.1016/j.compbiomed.2021.105124

Yang, Z., Jiang, M., Jiang, J., Xiong, R., Mao, C., Fang, S., et al. (2026). Co-classification network analysis reveals nodal dysfunction and dimension-specific alterations in obstructive sleep apnea-hypopnea syndrome. *Sleep Medicine* 139, 108764. doi:10.1016/j.sleep.2026.108764

Ye, G., Yin, H., Chen, T., Chen, H., Cui, L., and Zhang, X. (2021). Fenet: A frequency extraction network for obstructive sleep apnea detection. *IEEE Journal of Biomedical and Health Informatics* 25, 2848–2856. doi:10.1109/JBHI.2021.3050113

Ye, Y., Jiang, Z., Li, S., Lai, Z., Dahlweid, F.-M., Sun, H., et al. (2026). Sehpnet: Spectrally enhanced hierarchical patch network for sleep disorder evaluation via respiratory signals. *Biomedical Signal Processing and Control* 112, 108384. doi:10.1016/j.bspc.2025.108384

Yeo, M., Byun, H., Lee, J., Byun, J., Rhee, H.-Y., Shin, W., et al. (2022). Robust method for screening sleep apnea with single-lead ecg using deep residual network: Evaluation with open database and patch-type wearable device data. *IEEE Journal of Biomedical and Health Informatics* 26, 5428–5438. doi:10.1109/JBHI.2022.3203560

Ylmaz, D., Yldz, M., Toprak, Y. U., and Yetkin, S. (2023). Obstructive sleep apnea detection with nonlinear analysis of speech. *Biomedical Signal Processing and Control* 84, 104956. doi:10.1016/j.bspc.2023.104956

Yoo, H., Kim, G., Kim, T., Kim, H. Y., Jung, Y. G., Hong, S. D., et al. (2026). Development of a multimodal obstructive sleep apnea diagnostic prediction model using two-dimensional facial images and clinical data. *IEEE Journal of Biomedical and Health Informatics* doi:10.1109/JBHI.2026.3678664. Early access

Yook, S., Kim, D., Gupte, C., Joo, E. Y., and Kim, H. (2024). Deep learning of sleep apnea-hypopnea events for accurate classification of obstructive sleep apnea and determination of clinical severity. *Sleep Medicine* 114, 211–219. doi:10.1016/j.sleep.2024.01.015

Yu, H., Liu, D., Zhao, J., Chen, Z., Gou, C., Huang, X., et al. (2022). A sleep apnea-hypopnea syndrome automatic detection and subtype classification method based on lstm-cnn. *Biomedical Signal Processing and Control* 71, 103240. doi:10.1016/j.bspc.2021.103240

Yue, H., Lin, Y., Wu, Y., Wang, Y., Li, Y., Guo, X., et al. (2021). Deep learning for diagnosis and classification of obstructive sleep apnea: A nasal airflow-based multi-resolution residual network. *Nature and Science of Sleep* 13, 361–373. doi:10.2147/NSS.S297856

Zan, X., Wang, D., Song, C., Liu, F., Xian, X., and Berry, R. (2025). Weakly supervised deep learning for monitoring sleep apnea severity using coarse-grained labels. *IEEE Transactions on Automation Science and Engineering* 22, 15227–15240. doi:10.1109/TASE.2025.3566682

Zarei, A., Beheshti, H., and Asl, B. M. (2022). Detection of sleep apnea using deep neural networks and single-lead ecg signals. *Biomedical Signal Processing and Control* 71, 103125. doi:10.1016/j.bspc.2021.103125

Zhang, C., Yu, L., Li, L., Zeng, P., and Zhang, X. (2024). Screening for moderate to severe obstructive sleep apnea by using heart rate variability features based on random forest algorithm. *Sleep and Breathing* 28, 2521–2530. doi:10.1007/s11325-024-03151-9

Zhang, C., Zhang, C., Jin, Z., Yu, K., Wei, S., Zhang, M., et al. (2025a). The value of cough sound monitoring via an audio-enabled smartwatch for osa screening in copd patients: a cross-sectional exploratory study. *Frontiers in Medicine* 12, 1650014. doi:10.3389/fmed.2025.1650014

Zhang, D. H., Zhou, J., Wickens, J. D., Veale, A. G., and Hallum, L. E. (2025b). Apnea and hypopnea event detection using eeg, emg, and sleep stage labels in a cohort of patients with suspected sleep apnea. *Biomedical Signal Processing and Control* 105, 107628. doi:10.1016/j.bspc.2025.107628

Zhang, H., Fu, B., Su, K., and Yang, Z. (2023a). Long-term sleep respiratory monitoring by dual-channel flexible wearable system and deep learning-aided analysis. *IEEE Transactions on Instrumentation and Measurement* 72, 1–9. doi:10.1109/TIM.2023.3289535

Zhang, R., Zheng, K., Wang, Y., Zhou, W., Tian, L., and Liu, G. (2026a). Lightweight cosine convolution network for sleep apnea detection with single-lead ecg. *Neurocomputing* 669, 132457. doi:10.1016/j.neucom.2025.132457

Zhang, X., Li, F., Fang, Y., Zhong, L., Ding, K., Chen, H., et al. (2026b). Novel event-based peripheral oxygen saturation metrics provide complementary information for osa classification. *IEEE Transactions on Biomedical Engineering* PP, 1–9. doi:10.1109/TBME.2026.3665589

Zhang, Y., Zhou, L., Zhu, S., Zhou, Y., Wang, Z., Ma, L., et al. (2025c). Deep learning for obstructive sleep apnea detection and severity assessment: A multimodal signals fusion multiscale transformer model. *Nature and Science of Sleep* 17, 1–15. doi:10.2147/NSS.S492806

Zhang, Z., Conroy, T. B., Krieger, A. C., and Kan, E. C. (2023b). Detection and prediction of sleep disorders by covert bed-integrated rf sensors. *IEEE Transactions on Biomedical Engineering* 70, 1208–1223. doi:10.1109/TBME.2022.3212619

Zhang, Z., Wu, W., Fu, Z., Han, Z., Zhang, J., Sun, C., et al. (2025d). Real-time sleep apnea detection and prediction from single-channel airflow. *IEEE Transactions on Instrumentation and Measurement* 74, 1–9. doi:10.1109/TIM.2025.3554899

Zhang, Z., Wu, W., Wang, Q., Liu, J., Sun, Q., Ma, D., et al. (2026c). Sleep apnea prediction based on dynamical features from single-channel airflow. *Biomedical Signal Processing and Control* 117, 109608. doi:10.1016/j.bspc.2026.109608

Zhao, A., Hao, B., Liu, S., Qiu, X., Ming, X., Yang, X., et al. (2024a). A prediction nomogram of severe obstructive sleep apnea in patients with obesity based on the liver stiffness and abdominal visceral adipose tissue quantification. *Nature and Science of Sleep* 16, 1515–1527. doi:10.2147/NSS.S475534

Zhao, T., Li, Z., Liu, W., Fu, X., Zhou, Y., and Zhou, L. (2024b). Unconstrained respiratory event detection using a flexible tactile sensor. *IEEE Sensors Journal* 24, 30095–30103. doi:10.1109/JSEN.2024.3442240

Zhao, Y., He, H., Gao, W., Xu, K., and Ren, J. (2024c). Dm-iacnn: A dual-multiscale interactive attention-based convolution neural network for automated detection of sleep apnea. *IEEE Transactions on Instrumentation and Measurement* 73, 1–10. doi:10.1109/TIM.2024.3420355

Zhao, Y., He, H., Wang, Q., Yu, L., and Ren, J. (2025). Se-msresnet: A lightweight squeeze-and-excitation multi-scaled resnet with domain generalization for sleep apnea detection. *Neurocomputing* 620, 129201. doi:10.1016/j.neucom.2024.129201

Zhou, G., Zhao, W., Zhang, Y., Zhou, W., Yan, H., Wei, Y., et al. (2024). Comparison of OPPO watch sleep analyzer and polysomnography for obstructive sleep apnea screening. *Nature and Science of Sleep* 16, 125–141. doi:10.2147/NSS.S438065

Zhou, S.-J., Yang, R., Wang, L.-L., Qi, M., Yuan, X.-F., Wang, T.-T., et al. (2023). Measuring sleep stages and screening for obstructive sleep apnea with a wearable multi-sensor system in comparison to polysomnography. *Nature and Science of Sleep* 15, 353–362. doi:10.2147/NSS.S406359

Zou, L. and Liu, G. (2024). Multiscale bidirectional temporal convolutional network for sleep apnea detection based on wearable photoplethysmography bracelet. *IEEE Journal of Biomedical and Health Informatics* 28, 1331–1340. doi:10.1109/JBHI.2023.3335658
